# Linezolid in addition to standard antibiotic treatment for Staphylococcus aureus bacteraemia: study protocol for a randomised, placebo-controlled trial

**DOI:** 10.1136/bmjopen-2026-118509

**Published:** 2026-04-20

**Authors:** Natalie Rose, Nadine S Bernasconi, Michaela Schumacher, Laura Werlen, David Büchel, Maja Weisser, Severin B Vogt, Elisabeth Wehrle-Wieland, Anna Conen, Maria C Thurnheer, Yonas Martin, Michèle Birrer, Marco Bongiovanni, Werner C Albrich, Urs Karrer, Adrian Schibli, Stephan Harbarth, Matthaios Papadimitriou-Olivgeris, Matthias Briel, Barbara Hasse, Nina Khanna, Richard Kuehl, Benjamin Speich

**Affiliations:** 1 Division of Infectious Diseases, University Hospital Basel, Basel, Switzerland; 2 Department of Clinical Research, University Hospital Basel and University of Basel, Basel, Switzerland; 3 Division of Clinical Pharmacology and Toxicology, University Hospital Basel, Basel, Switzerland; 4 Division of Infectious Diseases, Claraspital Basel, Basel, Switzerland; 5 Department of Infectious Diseases and Infection Prevention, Cantonal Hospital Aarau, Aarau, Switzerland; 6 Department of Infectious Diseases, University Hospital Inselspital and University of Bern, Bern, Switzerland; 7 Department of General Internal Medicine / Infectious Diseases, Hôpital du Jura, Delémont, Switzerland; 8 Division of Infectious Diseases, Ente Ospedaliero Cantonale, Lugano, Switzerland; 9 Division of Infectious Diseases, Infection Prevention and Travel Medicine, HOCH Health Ostschweiz, Cantonal Hospital St. Gallen, St. Gallen, Switzerland; 10 Division of Infectious Diseases, Department of Medicine, Cantonal Hospital Winterthur, Winterthur, Switzerland; 11 Division of Infectious Diseases and Infection Prevention, Stadtspital Zürich, Zürich, Switzerland; 12 Infection Control Program and World Health Organization Collaborating Centre, Faculty of Medicine, Geneva University Hospitals, Geneva, Switzerland; 13 Infectious Diseases Service, Lausanne University Hospital and University of Lausanne, Lausanne, Switzerland; 14 CLEAR-Methods Center, Division of Clinical Epidemiology, Department of Clinical Research, University Hospital Basel and University of Basel, Basel, Switzerland; 15 Department of Infectious Diseases and Hospital Epidemiology, University Hospital and University of Zurich, Zürich, Switzerland

**Keywords:** Antibiotics, Clinical Trial, INFECTIOUS DISEASES

## Abstract

**Introduction:**

*Staphylococcus aureus* (*S. aureus*) bacteraemia is a common and severe infection. With mortality rates ranging from 20–30% and long-term impairments in over a third of survivors, better treatments are urgently needed. Linezolid, a well-established treatment for pneumonia and complicated skin infections, has been shown in preclinical studies to strongly suppress *S. aureus* virulence factors critical to bacterial persistence and tissue damage. Hence, we aim to investigate whether the addition of linezolid to standard therapy in patients with *S. aureus* bacteraemia leads to an overall improvement in patient-relevant outcomes.

**Methods and analysis:**

We will conduct a two-arm, parallel-group, multicentre, randomised controlled trial (Linezolid Plus Standard of Care) in 12 hospitals in Switzerland with blinded treating physicians, patients and outcome assessors. Hospitalised patients aged ≥18 years with *S. aureus* bacteraemia will be eligible. Patients will receive standard antibiotic treatment as prescribed by the treating physician. Within 72 hours of collection of the blood sample yielding the first positive blood culture, patients will be enrolled and randomised 1:1 to receive either adjunctive linezolid (600 mg orally two times per day for 5 days) or placebo. To determine patient-relevant outcomes, we implemented a comprehensive patient-representative consultation process. Consequently, we will use the desirability of outcome ranking (DOOR) established for *S. aureus* bacteraemia as the primary outcome at 90 days. The hierarchical composite DOOR outcome includes the following four components, ranked from most to least important: (1) survival, (2) return to level of function before *S. aureus* infection, (3) complications leading to treatment changes and serious adverse reactions; and (4) hospital length of stay. This approach will allow us to analyse the win ratio, that is, whether patients receiving linezolid have a better DOOR rank compared to patients in the placebo group. We calculated a target sample size of 606 patients providing 90% power at a two-sided significance level of 0.05.

**Ethics and dissemination:**

Ethical approval was received from the Ethics committee for Northern and Central Switzerland (BASEC number 2025-00655). Eligible patients will be informed about the study by the local study team and asked for written consent if they wish to participate. For patients unable to provide informed consent, an appropriate substitute (ie, a close relative or a physician not involved in the research project) may make decisions based on the presumed wishes and the best interest of the patient. The patient’s own consent will be obtained as soon as their condition permits. Results will be published in peer-reviewed journals and in laymen's terms through various channels (social media, Swiss national portal HumRes).

**Trial registration number:**

NCT06958835.

**STRENGTHS AND LIMITATIONS OF THIS STUDY**

- A randomised trial in 12 Swiss hospitals with blinded participants, physicians and outcome assessors to minimise risk of bias.
- Besides blinding, the trial will be embedded as much as possible in clinical routine care, thereby enhancing external validity of the results.
- The primary endpoint is a hierarchical composite incorporating multiple clinical measures ranked by patient representatives.
- Due to limited available resources, the trial is not powered to detect differences in mortality.

## Introduction

### Background and rationale

 Among bacterial causes of death, *Staphylococcus aureus (S. aureus*) ranks first in high-income countries and second worldwide, after tuberculosis.^1^ Bacteraemia is a frequent manifestation of *S. aureus* infection affecting 20–30 in 100 000 persons annually.^2^,^4^ Incidence rates have increased in recent years, driven by an ageing population and greater use of implantable medical devices.^24^,^6^ Despite active standard antibiotic therapy, 20–30% of patients with *S. aureus* bacteraemia die within 90 days,^7 8^ a rate that has remained unchanged for decades^9 10^ and represents the highest mortality among common bloodstream infections.^1^ In addition, *S. aureus* bacteraemia spreads to secondary body sites in 6–44% of cases,^11^,^14^ causing prolonged impairment in up to one-third of survivors^15^ and is associated with a relapse rate of 4–10%.^16^,^18^

*S. aureus* is known for its ability to survive and rapidly adapt to new environments within the human body.^19 20^ Employing a wide range of virulence factors such as toxins to attack human (immune) cells^21^ and proteases to invade human tissue,^22^ it often elicits a strong local inflammatory response. It can be inferred that most of the damage to the body results from direct and indirect effects of these virulence factors. The impact of this diffuse tissue damage and associated physiological stress extends well beyond the acute phase of infection. Notably, 40–60% of deaths in people with *S. aureus* bacteraemia occur after the initial episode, with causes of death often not clearly attributable to the infection itself.^11 23^ Similar patterns have been reported in patients with sepsis.^24^ Beyond mortality, *S. aureus* infections frequently lead to long-term functional and cognitive impairments, but the causes and magnitude of these impairments have not been fully elucidated.^25 26^

Treatment guidelines for *S. aureus* bacteraemia recommend cefazolin or anti-staphylococcal penicillins for methicillin-sensitive *S. aureus* and vancomycin, daptomycin or ceftobiprole for methicillin-resistant *S. aureus*.^27^ These antibiotics demonstrate in vitro bactericidal activity by targeting the bacterial cell membrane and wall. However, exposure to sub-inhibitory concentrations may even lead to increased toxin production in *S. aureus*.^28^ We thus hypothesise that adding an antibiotic agent which inhibits such virulence factor expression could reduce both direct and indirect organ damage, thereby improving patient outcomes.^29^ Preclinical studies have shown that linezolid significantly suppresses the expression of *S. aureus* virulence factors,^28^ making it a promising candidate for this approach. Expert opinion and some guidelines advocate the use of this treatment combination therapy for severe infections or for *S. aureus* strains with known toxin expression.^30^,^33^ However, robust evidence from randomised controlled trials (RCTs) to support this approach is still lacking.

### Objectives

Despite rapid clearance of *S. aureus* from the blood with appropriate antibiotic therapy,^12^ mortality and morbidity remain high. We thus hypothesise that direct and indirect damage is induced very early in the course of infection and expect the highest therapeutic benefit of linezolid within the first 3 days following detection of bloodstream infection with *S. aureus*. Hence, we are conducting the Linezolid Plus Standard of Care (LIPS) trial to determine whether the early addition of a 5-day treatment course of linezolid to standard of care improves outcomes at 90 days in participants with *S. aureus* bacteraemia as measured by the desirability of outcome ranking (DOOR).^34 35^

## Methods and analysis

### Trial design

**Figure 1:**
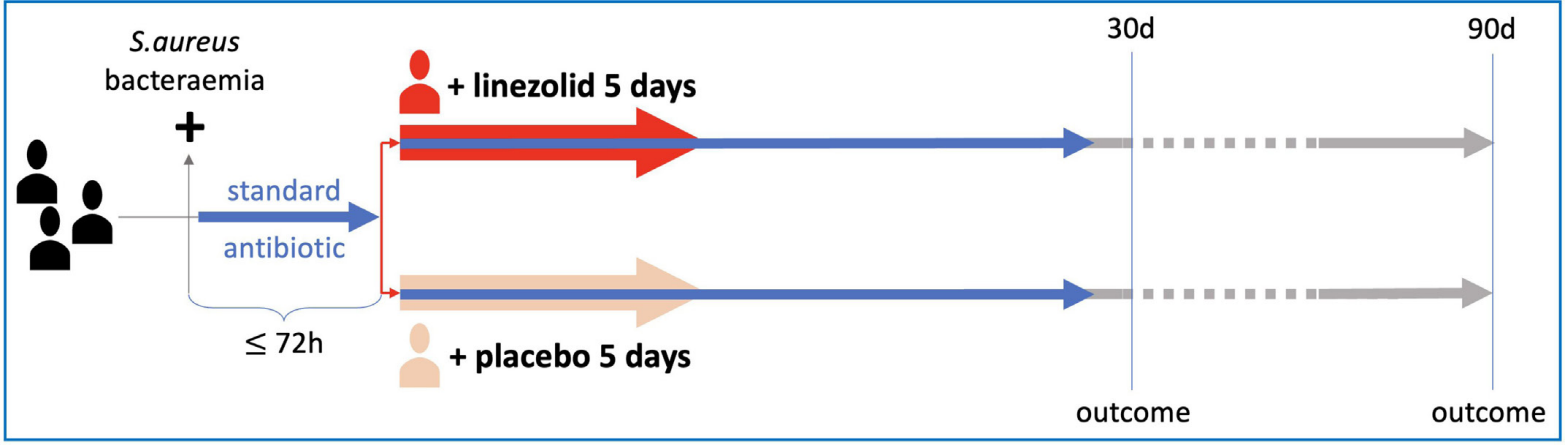
Schematic overview of the Linezolid Plus Standard of Care (LIPS) trial.

LIPS is a pragmatic, multicentre, 1:1 randomised, parallel-group, placebo-controlled, superiority trial with blinded participants, physicians and outcome assessors in the acute-care hospital setting (figure 1). We have chosen a pragmatic trial design closely following routine care (ie, most outcomes are derived from routine care medical records with only one additional follow-up consisting of a phone call and a quality-of-life (QoL) questionnaire at day 90). However, as some outcomes may be influenced by expectations from participants (ie, self-reported level of function) as well as the treating physicians (ie, hospital length of stay), we chose a blinded study design to eliminate this source of bias. The trial was registered prospectively on ClinicalTrials.gov (NCT06958835) where we also share the full study protocols which received ethical approval. Several aspects of the LIPS trial design have been aligned with the international *S. aureus* Network Adaptive Platform trial (SNAP)^36 37^ with the intention to join the SNAP network at a later time point.

### Patient and public involvement

Patient and public involvement (PPI) has been incorporated throughout the trial to enhance patient relevance and feasibility. We have been collaborating with four PPI representatives (two patient representatives who were affected by *S. aureus* bacteraemia as well as two patient experts). These PPI representatives have agreed to lend their expertise to the trial design, as well as during the conduct of the trial and the dissemination of the trial results. Before each meeting, PPI representatives receive lay-language briefings on trial progress and meeting objectives. Prior to finalising the first protocol and informed consent form drafts, three focus group sessions (2–4 hours each) were held to determine patient-relevant outcomes, minimise participant burden, broaden inclusion criteria and ensure that consent materials are clear and understandable (see details in online supplemental appendix 1 (table S1)). Future PPI activities after recruitment start will focus on guiding informed consent conversations with patients and relatives, contributing the patient perspective during interim safety assessments and helping shape dissemination strategies for the trial results (see details in online supplemental appendix 1 (table S2)).

### Setting and trial participants

We anticipate that patients will be recruited at the infectious disease departments of 12 Swiss hospitals (full list in online supplemental appendix 1 (text 1)). Hospitalised patients with blood cultures positive for *S. aureus* will be screened by the local site investigators or delegated personnel using the inclusion and exclusion criteria.

#### Inclusion criteria

- *S. aureus* grown from at least one blood culture.
- Hospitalised at a participating centre.
- Age ≥18 years.
- Written informed consent or fulfilling criteria for an emergency exemption from informed consent requirements.

#### Exclusion criteria

- Administration of the initial study drug treatment not feasible within 72 hours since the collection of the first positive blood culture with *S. aureus.*
- Documented history of positive blood cultures for *S. aureus* between 72 hours and 180 days prior to the eligibility assessment.
- Necrotising fasciitis.
- Currently receiving linezolid or clindamycin.
- Use of any monoamine oxidase A or B inhibitor (within the last 2 weeks).
- Hypersensitivity to linezolid or any other ingredients of the study drugs.
- Current severe thrombocytopenia (ie, <30×10^9^/L).
- Oral application of study drug not possible (per mouth or per gastric tube).
- Currently breastfeeding.
- The local treating team believes that death is imminent and inevitable.
- Patient is receiving end of life care and antibiotic treatment is not considered appropriate.
- The local treating team believes that participation in the study is not in the best interest of the patient.
- Any indication that the patient is unwilling to participate in the study, including an advance directive stating such unwillingness.

#### Recruitment

Eligible patients will be approached by the local study team as soon as possible. They will be informed about the LIPS trial by delegated study personnel and receive a copy of the study information in lay language including details of the intervention and potentially associated benefits and risks (prospective consent, online supplemental appendix 2). For patients unable to provide informed consent due to severe illness or other circumstances, the study team will check the patient’s chart for an advanced directive or similar statement of wishes. If no such statement is available, an appropriate substitute (ie, a close relative (next of kin consent; online supplemental appendix 3) or a physician not involved in the research project) may make decisions based on the presumed wishes and the best interest of the patient as recommended by Swissethics.^38^ Participants who were included after receiving consent from their next of kin or confirmation by an independent physician will be asked to provide retrospective consent as soon as their health status allows them to make an informed decision (retrospective consent; online supplemental appendix 4).

### Trial intervention and comparator

All patients with confirmed *S. aureus* bacteraemia will receive standard-of-care antibiotic treatment as prescribed by treating physicians. In addition, participants randomised to the intervention arm will receive oral linezolid 600 mg tablets for 5 days, two times per day. Accordingly, participants in the control arm will receive oral placebo tablets (Fagron) alongside standard of care (figure 1).

#### Criteria for discontinuing or dose modification

Study medication may be discontinued due to adverse events. The study medication is administered two times per day for 5 days (10 total doses) at approximately 12-hour intervals. If a strict 12-hour interval is impractical (eg, administration during the night), the first dosing intervals may be adjusted by up to ±6 hours according to standard practice. No further dose modifications are permitted, including for patients with renal impairment.

#### Strategies to improve adherence to intervention

The study medication will be administered by site personnel in accordance with standard routine drug administration practices. Missed doses will be documented with reasons in the electronic medical records or on a worksheet (for patients transferred to a non-participating hospital). The data will then be entered into the electronic case report form (eCRF). Participants discharged home before completing the 5-day course will receive instructions on proper storage and administration.

#### Concomitant interventions

All participants receive standard of care treatment for *S. aureus* bacteraemia as clinically indicated and according to local and international recommendations. This includes targeted antibiotic treatment for at least 2–6 weeks, mostly intravenous. The choice of standard of care antibiotic will not be influenced by the study but will be documented in the eCRF.

### Randomisation and blinding

Participants will be allocated 1:1 to either 5 days of linezolid or placebo in addition to standard of care treatment. The randomisation will be stratified by centre and intensive care unit (ICU) status at enrolment using block randomisation with varying random block sizes of 2 or 4. ICU includes all inpatient areas with the capacity for organ support, including invasive or non-invasive ventilation, vasopressors and/or inotropes and higher nursing staff-to-patient ratios than standard hospital wards. Intermediate Care Units that meet these criteria will also be considered as ICUs.

The trial statistician will generate the randomisation list and upload it into the data capture system REDCap.

The treatment allocation will not be revealed to any study staff or participants (ie, physicians, study nurses, participants and outcome assessors). Only the unique randomisation number will be visible for blinded personnel. The randomisation list will only be accessible to designated staff of the Hospital pharmacy of the University Hospital of Basel for manufacturing, labelling and blinding the study medication, and the data scientist handling the randomisation list. The study medication (10 tablets per container) with a unique randomisation number will be distributed to the study centres. For each unique randomisation number, study centres will receive a sealed, opaque envelope specifying whether the tablets contain linezolid or placebo. The treating physician will only be allowed to open an envelope if safety concerns require knowing a participant’s allocated intervention. All unblinding procedures must be documented in the eCRF. The integrity of the envelope seals will be verified during monitoring visits and at study close-out.

### Outcomes

Except for a study-specific phone call at day 90, most outcomes will be assessed from routinely collected data. Specific details about the definitions of endpoints and how they are assessed are reported in the full protocol (see online supplemental appendix 5; full trial protocol).

#### Primary outcome

The primary outcome utilises the DOOR, a hierarchical composite endpoint specifically developed as a patient-centred outcome for *S. aureus* bacteraemia.^39 40^ We adapted the original DOOR framework based on input from our patient representatives and the ongoing SNAP trial.^36^

In detail, the most important outcome for patient representatives was surviving the infection, followed by ‘being the same as before the disease’, meaning that they have the same level of function as before the *S. aureus* infection. Patient representatives with prior *S. aureus* bacteraemia considered blood culture results highly relevant during hospitalisation due to uncertainty about disease progression. This was particularly important because diagnostic evidence of persistent, relapsing or new *S. aureus* infection results in new diagnostic evaluation, interventions and/or new exposure to antibiotics with their associated toxicities. Therefore, we have implemented the third component *complications*, including *microbiological and clinical failure leading to treatment change*, *serious adverse reactions* and *adverse events leading to study drug discontinuation*. Hospital length of stay was also considered important by patient representatives and thus included as the fourth DOOR component.

**Figure 2:**
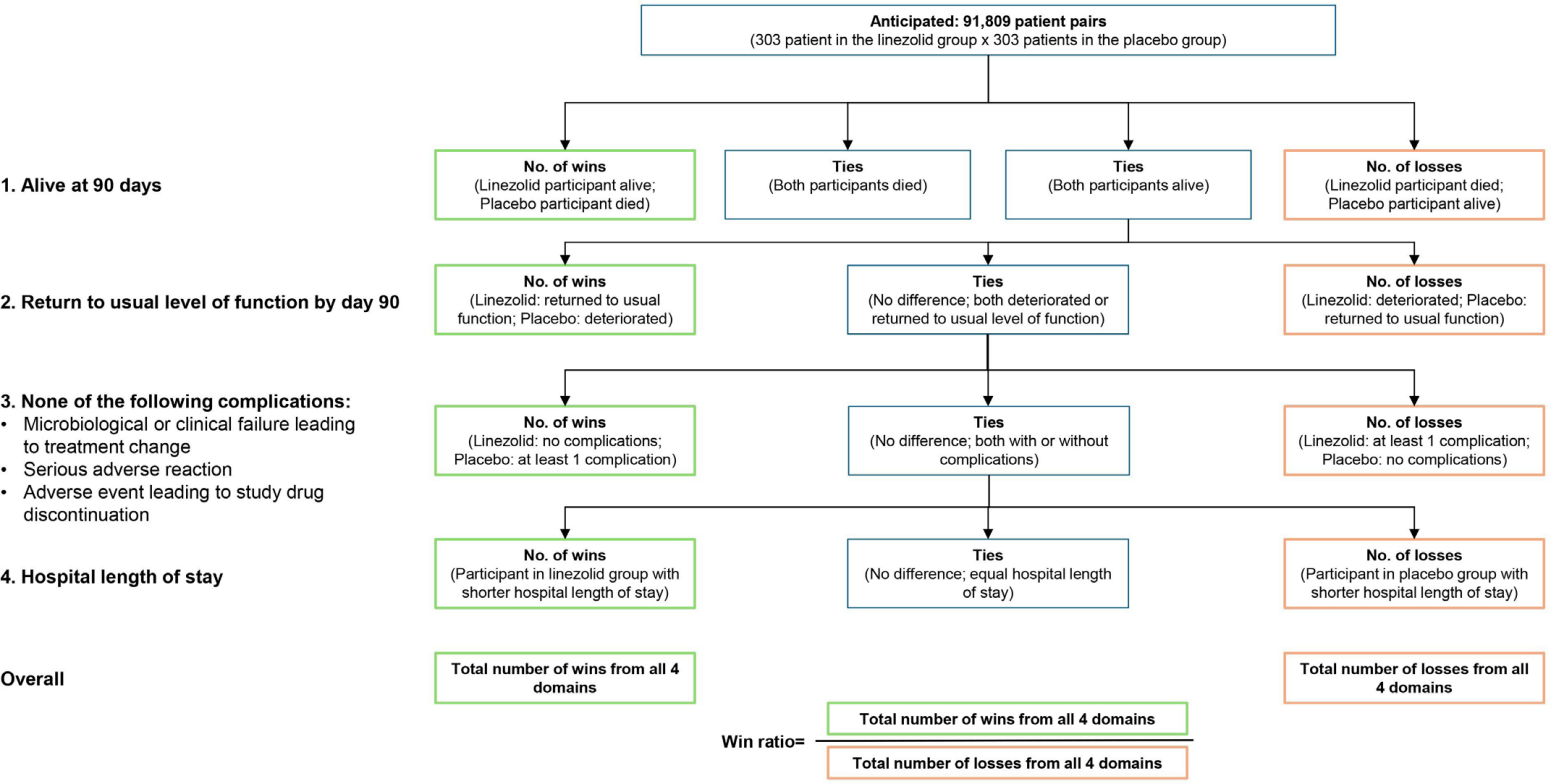
Primary hierarchical endpoint for the LIPS trial using four components. Schematic diagram of the hierarchical composite outcome used in the LIPS trial. Specific analytical details will be pre-specified in a separate statistical analysis plan.

Hence, our hierarchical composite endpoint, assessed at day 90, consists of four components (figure 2; online supplemental appendix 1 (table S3)), which are defined as follows:

1. *Alive at 90 days*: alive at 90 days after randomisation.
2. *Return to usual level of function by day 90*: binary endpoint defined as return to baseline value (or higher) according to the functional bloodstream infection score.^41^ The score was modified by the LIPS study team based on PPI input to increase resolution by adding an ‘out of hospital’ category (ie, level 5: no assistance but some limitations) and by combining the original in-hospital categories (‘hospitalised, not in ICU’; ‘hospitalised in ICU’; ‘admitted to long-term ventilator unit’) into a single in-hospital category (level 2). Baseline is defined as the best score within the 4 weeks before randomisation. The scores used will be the following:
  - 6: Out of hospital, able to complete daily activities without assistance (no limitations).
  - 5: Out of hospital, able to complete daily activities without assistance but with some limitations (eg, slow, pain).
  - 4: Out of hospital, unable to complete daily activities without assistance.
  - 3: Out of hospital; significant disability; requires a high level of care and assistance daily (this includes residential aged care and nursing homes).
  - 2: Hospitalised (or equivalent).
  - 1: On palliative care in terminal phases of life (in hospital or at home).
3. *Complications* consist of the following aspects:
  - *Microbiological failure leading to treatment change:* Any culture from a sterile site with growth of *S. aureus* between 14 and 90 days after randomisation associated with any type of adaptation of the treatment such as new intervention or surgery, or re-start, prolongation or change of antibiotic treatment initiated and documented by the treating physician. A sterile site means any site of the body where microorganisms are usually absent, that is, below the outer and inner colonised surfaces of the skin and mucous membranes. Positive sterile site cultures include growth of *S. aureus* from deep visceral and musculoskeletal abscesses obtained in a sterile manner.
  - *Clinical failure leading to treatment change:* Newly identified focus of *S. aureus* between 14 and 90 days after randomisation as determined by the site investigator associated with any type of adaptation of the treatment such as new intervention or surgery, or re-start, prolongation or change of antibiotic treatment initiated and documented by the treating physician. This includes clinical, radiological, microbiological and pathological findings.
  - *Serious adverse reaction:* A serious adverse event (SAE) determined to be definitely or probably related to the study drug by the local study physician.
  - *Any adverse event (irrespective of grade) leading to study drug discontinuation*.
4. *Hospital length of stay:* Duration of the index acute care hospital stay from randomisation to hospital discharge. A transfer to another acute care hospital is not considered a discharge but a continuation of the same hospital stay and is therefore included in the total duration. Days after transfer to rehabilitation centres or switch to outpatient parenteral ambulatory treatment will not be included.

The overall distribution of rankings will be compared between the control and intervention arms, with the final treatment effect estimate being the win ratio, that is, the probability that a participant randomly assigned to the intervention arm has a superior DOOR compared with a participant randomly assigned to the control arm.^35^

#### Secondary and safety outcomes

Secondary outcomes will be the following:

- All-cause mortality at 90 days.
- Time to death up to day 90.
- Proportion of participants back to their usual level of function at day 90.
- Microbiological failure at 14–90 days leading to treatment change.
- Early microbiological failure at 5–13 days leading to treatment change.
- Clinical failure at 14–90 days leading to treatment change.
- Early clinical failure at 5–13 days leading to treatment change.
- Hospital length of stay after randomisation.
- Time to being discharged alive (assessed by day 90).
- Number of days without being on the ICU up to day 90.
- Number of days alive and free of antibiotics up to day 90.
- Mental health at day 90 (SF-36 questionnaire).
- Physical health at day 90 (SF-36 questionnaire).
- Persistent bacteraemia: *S. aureus*-positive blood culture on day 5.
- ≥2 systemic inflammatory response syndrome criteria on day 5.
- Change in C-reactive protein on day 5.
- Development of new antibiotic drug resistance in *S. aureus.*

Adverse events and safety outcomes will be the following:

- Serious adverse reactions until day 90.
- Adverse events leading to study drug discontinuation.
- Adverse events of special interest: clinical signs of serotonin toxicity, laboratory signs of myelosuppression, hyperlactataemia, acute kidney injury, *Clostridioides difficile*-associated diarrhoea within 90 days.
- Serious adverse events.

#### Other outcomes

The following other outcomes of interest will only be collected in selected study centres and therefore only in a subset of participants.

- Linezolid trough plasma concentration at day 4 or 5.
- Linezolid minimal inhibitory concentration of the respective *S. aureus* strain from the blood.

### Participant timeline

All study participants are hospitalised; hence the LIPS trial will mainly use data generated as part of clinical routine during hospitalisation. In brief, baseline data (eg, year of birth, sex, risk factors), microbiological results (eg, blood culture results, resistance patterns), laboratory results (eg, blood count, renal function) and information on concomitant treatments (standard antibiotics, source control procedures) will be extracted from the electronic medical record. Participants will be treated and monitored according to standard clinical routine. If a treating physician suspects an adverse event (eg, serotonin toxicity, myelosuppression), they will order any necessary diagnostic assessments as per routine procedures.

The outcomes ‘alive at day 90’ and ‘return to usual level of function by day 90’ will be assessed via a standardised phone interview on day 90 (provided that the participant is still alive). In addition, participants will receive a QoL questionnaire at day 90 to assess physical health and mental health. If repeated contact attempts with the participant are unsuccessful, study personnel will reach out to the designated emergency contact, relatives and/or the general practitioner to determine the participant’s vital status and other components of the primary endpoint. Online supplemental appendix 1 (table S4) provides a detailed overview of the timing of data entry in the eCRF.

### Data collection methods and data management

Relevant clinical study data for each participant are recorded in eCRFs via the web-based electronic data capture system implemented in REDCap. The participant’s name and address will not be recorded. REDCap includes guidance for study sites on how to perform data entry and will also be used for query handling. The identity of the local investigator or delegated personnel entering data, and date and time of data entry will be recorded as meta-data in the study database.

### Monitoring

The trial team will perform risk-based centralised monitoring through REDCap by systematically reviewing key aspects of the data, including study drug administration, primary outcome measures and close-out visits for all participants. In the event of missing or inconsistent data within REDCap, the sponsor team will raise queries and contact the respective local study team for clarification.

In addition, on-site monitoring visits will be conducted by the Department of Clinical Research of the University Hospital Basel. During these visits, a proportion of source data will be verified against the study records. Source data comprise all relevant study documents, such as informed consent forms, online SAE reports and patient information recorded in the hospital information system. These documents will be accessible at all sites, either in paper or electronic form.

### Sample size

We collected retrospective data on the DOOR components from all *S. aureus* bacteraemia patients treated at the University Hospital Basel in 2022 (n=119). Our assumptions for the sample size were based on these data along with what is known from the literature to derive plausible estimates for the DOOR components. Patients who died in the first 3 days after the diagnosis of *S. aureus* bacteraemia (n=8) were excluded to prevent an overestimation of mortality in the planned trial (of note, most of these 8 patients would likely have died before they could have been included in our study). Following extensive consultations with patient representatives, we established that for mortality at 90 days, a relative difference of 10% would be a meaningful improvement to standard of care (equating to an absolute difference of 2.2%). For the remaining components, the following relative reductions were deemed acceptable: 15% for level of function and 20% for complications and length of hospital stay.

The sample size was calculated using a simulation approach, for which 1000 synthetic datasets for each combination of plausible values for the relevant parameters were generated. We calculated the p value of the win ratio for each of the simulated datasets and tested for statistical significance at α=0.05 using the unmatched win-ratio approach described in Pocock *et al*.^42^ The final sample size is based on the following specific assumptions:

- The statistical tests are conducted at a two-sided significance level of α=0.05 with a desired power of 90%.
- The two treatment arms are equal in size (1:1 allocation).
- The proportion of deaths within 90 days in the control group is expected to be 22.5% and 20.3% in the linezolid group (relative reduction of 10%; absolute reduction of 2.2%).
- The proportion of participants with worse level of function at day 90 (vs baseline) is expected to be 25.0%^15^ in the control group and 21.3% in the linezolid group (relative reduction of 15%; absolute reduction of 3.7%).
- The proportion of participants with any complications (third DOOR component) is expected to be 6.3% in the control group and 5.0% in the linezolid group (relative reduction of 20%; absolute reduction of 1.3%).
- We modelled hospital length of stay using a log-normal model with any of the complications listed in the definition above as independent variables; the parameters for this model were derived from retrospective internal data, and the expected 20% relative reduction in hospital length of stay due to the intervention was applied to the mean of the log-transformed data when generating synthetic datasets.

Missing data were accounted for in the simulations (ie, 10% for worse level of function and only 3% for all other components as these data will be available from routinely collected data). In the case of missing DOOR components, the comparison among participants was continued with the DOOR component one hierarchy level below.

Under the assumptions listed above, 550 participants are required to show a significant effect of the intervention. To account for post-randomisation withdrawal of consent and minimise the risk of being underpowered, we increased the target sample size by 10%. Hence, we plan to include 606 participants (303 participants in each study arm).

### Statistical analysis

Analyses will be performed on the full analysis set (ie, all randomised participants) and the per protocol dataset (ie, those who received at least 7 out of 10 doses of linezolid or placebo). The primary estimand to be calculated in this study is the effect (summarised as the win ratio) of being randomly assigned to treatment with linezolid in the patient population (ie, equivalent to an intention-to-treat analysis).

A detailed analysis plan will be written before closing the study database. Of note, we are currently conducting a systematic scoping review, assessing current practices for analysing hierarchical composite outcomes.^43^ These results will inform our pre-specified analysis plan. Furthermore, we will consider further developments in risk-score classifications for *S. aureus* bacteraemia (eg, for potential use of a matched win-ratio vs unmatched win-ratio). The statistical analysis plan will be made publicly available (on ClinicalTrials.gov). At present, we intend to conduct the following analyses: Treatment and placebo will be compared based on the win-ratio approach as described by Pocock and colleagues^42^: In brief, each participant in the treatment group will be compared with each participant in the placebo group. When comparing two participants, the winner will be determined by the first component of the DOOR in which the two participants differ. The following two scenarios will be considered a tie: (i) when both participants die within 90 days or (ii) when participants have the same outcomes for all DOOR components, including length of hospital stay. The win ratio will be calculated as the number of pairwise comparisons won by participants receiving linezolid divided by the number won by participants receiving placebo. We will reject the null hypothesis that the win ratio is equal to 1 if the p value is less than 0.05. Detailed information on how secondary outcomes will be analysed is provided in the full study protocol (online supplemental appendix 5).

We will conduct sub-group analyses for the primary outcome and all DOOR components separately, assessing if treatment effects differ by sex (male vs female), ICU status at baseline (participants on ICU vs participants not on ICU) and focus of infection (vascular catheter, skin and soft tissue, endocarditis, osteoarticular, pneumonia, other focus or focus not identified). All subgroup analyses will be exploratory in nature.

After 50 participants have completed their 90-day follow-up, and again after 200 participants, the independent Data and Safety Monitoring Board (DSMB), which includes external clinical experts, an external statistician, as well as patient and public representatives, will meet to discuss summary statistics about safety outcomes. In addition, adherence to treatment will be assessed in these interim analyses. If the proportion of non-adherence is above 10% (ie, more than 10% of participants received fewer than 7 of the planned 10 doses), the study team will assess the reasons why doses were missed and implement actions (eg, additional training) to increase adherence.

Missing data were incorporated in our sample size simulation for the individual DOOR components. Hence, the primary analysis will be performed on the available data, and the next DOOR component will be considered in the pairwise comparison in case of missing data. For the secondary outcomes, we will consider multiple imputation if the proportion of excluded participants is larger than 5% (the imputation strategy will be pre-specified for each outcome in the separate data analysis plan).

### Protocol amendments

An amendment is currently in preparation. Its main purpose is to define the following exemptions from SAE reporting: (1) SAE in the context of pre-existing foci; (2) SAEs in the context of pre-planned procedures; and (3) SAEs in the context of events clearly related to *S. aureus* bacteraemia and documented as study outcomes. In addition, deaths captured as part of the primary outcome will not be reported as SAEs, provided they are not related to the study drug. The SAE reporting period will be shortened from the last patient assessment to 30 days after discontinuation of the intervention (with the exception of *C. difficile*-associated diarrhoea). Further details are provided in online supplemental appendix 1 (text 2). The amended protocol will also include a more precise definition of the DSMB’s scope and outsourcing the review of SAEs to an independent physician. The amended protocol is still in preparation and has not yet been submitted to Swissethics. For transparency, future versions of the approved full trial protocol will be uploaded on ClinicalTrials.gov (NCT06958835).

## Ethics and dissemination

The LIPS trial received approval from the Ethics committee for Northern and Central Switzerland (BASEC number 2025-00655) and the Swiss Agency for Therapeutic Products (Swissmedic). Patients will be asked for prospective written consent. For patients not able to provide consent (eg, due to the emergency condition), appropriate alternative approaches (ie, a close relative or a physician not involved in the research project) may make decisions based on the presumed wishes and the best interest of the patient as recommended by Swissethics.^38^ A retrospective consent from these participants will be collected as soon as their health status allows them to make an informed decision.

The full study protocol is publicly available on ClinicalTrials.gov (NCT06958835), where the trial was prospectively registered on the 6 May 2025. The LIPS trial recruited the first patient on 6 October 2025. At the time of manuscript submission (17 February 2026), 47 patients have been recruited and 5 out of 12 planned sites were active. For liability reasons and in compliance with Swiss laws and regulations, an insurance policy is provided by the Sponsor University Hospital Basel. A detailed statistical analysis plan will be finalised before database lock. The statistical analysis plan will be made publicly available on ClinicalTrials.gov. After publication of the main results, the full dataset will be submitted to the Data Access Committee of the Faculty of Medicine (MF-DAC) at the University of Basel. Other researchers interested in using the study data may contact the independent MF-DAC of the University of Basel to request access.

The results of the LIPS trial will be published in peer-reviewed journals regardless of the study findings. In collaboration with the PPI representatives, lay summaries will be drafted for dissemination via various channels (eg, social media, Swiss national portal HumRes). The study teams may provide each study participant with the lay summary of the trial results on request and inform them where they can find the study results.

## Discussion

The LIPS trial was designed to evaluate whether early addition of a 5-day course of linezolid to standard of care improves outcomes in participants with *S. aureus* bacteraemia. The primary endpoint is a hierarchical composite endpoint, chosen to determine whether patients receiving linezolid *do better overall* compared with those in the control group.^44^ A hierarchical composite endpoint was selected for the following reasons: First, hierarchical composite outcomes allow for the integration of several clinically meaningful measures, thus capturing whether patients experience overall better outcomes with the intervention (‘Good Studies Evaluate the Disease While Great Studies Evaluate the Patient’).^39^ Second, in contrast to traditional composite outcomes, hierarchical composite endpoints avoid the limitation of weighting events of differing importance equally.^45^ Third, given the available resources, we were unable to design an RCT adequately powered for mortality. Instead, employing a hierarchical composite endpoint provides greater statistical efficiency and allows us to sufficiently power the LIPS trial within the constraints of our resources.^46^ However, it is worth mentioning that hierarchical composite endpoints also have certain limitations, such as difficulties in interpreting effect sizes and challenges in adjusting for stratification factors in the analysis.^44 47^

Importantly, the LIPS trial was designed in close collaboration with PPI representatives, who actively contributed to the selection of patient-centred endpoints for the hierarchical composite endpoint. We are therefore confident that the findings of the LIPS study will be highly relevant to patients. If linezolid proves effective for *S. aureus* bacteraemia, it has the potential to meaningfully improve clinical management and outcomes for millions of patients worldwide.

## Supplementary material

10.1136/bmjopen-2026-118509 online supplemental file 1

10.1136/bmjopen-2026-118509 online supplemental file 2

10.1136/bmjopen-2026-118509 online supplemental file 3

10.1136/bmjopen-2026-118509 online supplemental file 4

10.1136/bmjopen-2026-118509 online supplemental file 5
