# Supplementary material for: Linezolid in addition to standard antibiotic treatment for Staphylococcus aureus bacteraemia: study protocol for a randomised, placebo-controlled trial
Source: BMJ Open. 2026 Apr 20;16(4):e118509. doi: 10.1136/bmjopen-2026-118509 (PMC13110666; doi:10.1136/bmjopen-2026-118509)
Supplement: online supplemental file 1 [file bmjopen-16-4-s001.docx]

Appendix 1:

**Text S1**: The 12 original study sites that agreed to participate within the LIPS trial. Changes might be possible and will be reported on clinicaltrials.gov (NCT06958835).

● Basel, University Hospital Basel (Sponsor Investigator centre)

● Aarau, Cantonal Hospital Aarau

● Basel, St. Clara Hospital Basel

● Bern, University Hospital Bern

● Delémont, Cantonal Hospital Jura

● Lausanne, University Hospital Lausanne

● Lugano, Cantonal Hospital Lugano

● Geneva, University Hospital Geneva

● St. Gallen, HOCH Health Ostschweiz, Cantonal Hospital St. Gallen

● Winterthur, Cantonal Hospital Winterthur

● Zürich, University Hospital Zürich

● Zürich, City Hospital Zürich

**Text S2:** Intended amendment, defining exemptions for the reporting of serious adverse events.

**Reporting of SAEs**

Except for the exemptions listed below, all SAEs must be reported immediately and within a maximum of 24 hours of becoming aware of the SAE to the Sponsor-Investigator of the study. The Sponsor-Investigator or a sponsor-delegated physician will review the SAE report in a separate eCRF form and provide feedback to the site in case of discrepancies. In addition, aggregated SAEs will also be evaluated by the DSMB during the interim analyses (including external experts as well as patient and public representatives; see also chapter *11.4.4 Interim analysis* in full trial protocol).

**Exemptions from SAE reporting**

The LIPS trial includes patients who at the time of randomisation are already in a life-threatening condition due to *S. aureus* bacteraemia. Participants are included early in the course of their infection to assess whether prompt administration of linezolid improves patient outcomes. Therefore, the diagnostic work-up to determine the aetiology of *S. aureus* bacteraemia, as well as the therapeutic management, is often not completed at the time of randomisation. *S. aureus*-infection-related events are well-documented in the patient population and are thus exempted from Serious Adverse Event (SAE) reporting, as these events are considered part of the natural progression of the patients’ underlying condition. Excluding infection-related medical occurrences from SAE reporting does not pose an additional risk to patient safety or the overall risk profile of the trial. Since patients in the LIPS trial remain in the care of the treating physicians not involved in the LIPS trial, their medical care is not influenced by SAE reporting. Linezolid has a well-characterized safety profile, and the focus of SAE reporting will remain on SAEs not expected based on the natural history of *S. aureus* bacteraemia in this patient population.

1. **Not reported as SAEs in the context of pre-existing infectious foci**

An infectious focus that is clinically apparent at the time of study inclusion shall not be recorded as an SAE, irrespective of the timing of its formal diagnostic confirmation.

Examples:

- - - Clinical picture of septic arthritis of the knee at the time of study inclusion; puncture and culture of joint fluid one day later confirms the clinical diagnosis.
    - Severe back pain and fever at the time of study inclusion; MRI confirms the diagnosis of spondylodiscitis two days later.
    - High fever, prosthetic cardiac valve in place and 6/6 blood cultures positive for *S. aureus* at the time of inclusion; Echocardiography confirms the diagnosis of endocarditis one day later.

1. **Not reported as SAEs in the context of pre-planned procedures**

Any therapeutic procedure documented as planned at the time of study inclusion shall not be considered an adverse event.

Examples:

- Participant with septic arthritis of the knee, and surgical debridement is planned for one day after study inclusion.
- Participant with spondylodiscitis, and surgical debridement is planned for three days after study inclusion.
- Participant with endocarditis, and a valve replacement is planned for several days after study inclusion.

1. **Not reported as SAEs in the context of events clearly related to *S. aureus* bacteraemia and documented as study outcomes**

Several of the events that classify formally as an SAE are commonly caused directly by *S. aureus* bacteraemia and are therefore key endpoints in the LIPS trial. Hence, conditions or treatments due to new metastatic infectious foci of *S. aureus* or due to progress or relapse of the initial *S. aureus* infection should only be reported as SAEs if the event is considered definitely or probably related to the study drug, based on the judgment of the local PI or a delegated physician.

Examples:

- Participant needing surgery due to a new septic arthritis of the hip with *S. aureus* after stopping the antibiotic treatment
- Participant needing a second surgery due to uncontrolled spinal infection with *S. aureus*

**Exemption from reporting deaths as SAEs**

*S. aureus* bacteraemia has a high 90-day mortality of 20-30% and deaths are captured as part of the primary outcome. If a death occurring within 90 days from randomisation is due to *S. aureus* bacteraemia or natural causes, it should not be reported as an SAE. Thus, deaths should only be reported as SAEs if the local PI or delegated physician determines that they are definitely or probably related to the IMP.

**SAE reporting period**

The trial intervention will be administered for five days. Linezolid is rapidly metabolised with a half-life of 5-7 hours, resulting in elimination and plasma levels below the detection limit within approximately 35 hours after discontinuation of the treatment. More than 20 years of real-world experience with linezolid have shown that SAEs related to the study drug manifest during treatment or shortly thereafter, with the exception of *C. difficile*-associated diarrhoea, which can occur several months after treatment cessation.

Following a risk-adapted safety reporting strategy, SAEs will be assessed using medical records, recorded and reported from administration of the first IMP dose until 30 days after stopping the intervention (i.e. until day 35 after randomisation) – with exception of *C. difficile-* associated diarrhoea, which is recorded and reported until day 90.

If an investigator becomes aware of an SAE with a suspected causal relationship to the IMP that occurs after the end of the SAE reporting period or after the end of the clinical trial, the investigator shall nevertheless report the SAE to the sponsor.

**Table S1**: Conducted patient and public involvement activities before recruiting the first patient

| **PPI contribution** | **Outcome** |
| --- | --- |
| - Advised on the patient relevance of the proposed outcomes. - Informed the researchers about important aspects not covered by the proposed outcomes. - Discussed what the primary outcome should be and help with the exact definition. | - Trial outcomes that are relevant to patients were chosen and ranked in order of importance (DOOR outcome). - PPI representatives advised to include a patient-reported outcome measure on quality of life at day 90. |
| - Reviewed the eligibility criteria with a focus on inclusivity and making the trial results generalizable. | - Broad inclusion criteria were chosen as recommended by the PPI representatives. - Exclusion criteria focus on safety aspects. |
| - Provided feedback on how understandable the provided lay summary was. | - Parts of lay summary were flagged as needing further explanation or simplifications and edited accordingly. |
| - Advised on the chosen time points and if the study visits are useful and patient friendly. - Assessed burden for patients and identified ways to decrease burden, dropout rates and incomplete data selection. | - Outcomes may also be collected by asking the participant’s relatives or general practitioner. - Burden for participants with the proposed trial design was deemed very low. |
| - Defined the further study phases during which PPI is critical and what form such involvement should take (guided by SCTO template). | - PPI activities during and after trial conduct were planned (see chapter 6.2.2 *PPI activities after receiving approval from authorities* in full trial protocol). |
| - Provided feedback on draft patient information and informed consent form (ICF) in German. - Some PPI representatives will also assess patient information in other languages | - ICF is concise and comprehensible, giving enough information without frightening patients regarding their diagnosis. - Patient-relevant information on background of the underlying disease, study procedures, risks and adverse events in layman’s terms are included. |

**Table S2**: Planned patient and public involvement activities after recruiting the first patient

| **Planned PPI contribution** | **Objective** |
| --- | --- |
| - Advise on how to approach patients and relatives to inform them about study participation. | - Incorporating PPI feedback into trainings of study sites on how to approach patients and relatives in a sensitive manner. |
| - Participation in DSMB during prespecified interim analyses | - Including the patient perspective when serious adverse are assessed in the interim analysis. |
| - Discussing which of the results/effects are patient relevant and how to best visualise the data. | - We will report all outcomes independent of the assessed effect. The objective of this exchange is to receive feedback which effects are most relevant for patients and how data should be visualised so that they can be understood easily |
| - Advise on which form the results should be communicated. | - Assuring that the study results are disseminated to a wide audience. |
| - Support the communication of results in lay language. | - Ascertaining that the study results can be communicated to participating patients and the general public. |
| - Discussing the impact and the next steps with the researchers. | - Making sure that appropriate action is taken based on the study results taking into consideration the patient perspective. |
| - Evaluating the PPI involvement | - Learning how best to involve PPI in future collaborative research projects |

**Table S3**: Primary hierarchical endpoint for the LIPS trial using a desirability of Outcome Ranking (DOOR)

| **Rank** | **Alive at 90 days** | **Return to usual level of function by day 90** | **None of the following complications:**   - **Microbiological or clinical failure leading to treatment change** - **Serious adverse reaction** - **Adverse event leading to study drug discontinuation** | **Hospital length of stay** |
| --- | --- | --- | --- | --- |
| **1** | Yes | Yes | Yes (no complications occurred) | Ties will be resolved using the length of hospitalisation |
| **2** | Yes | Yes | No (complication occurred) |  |
| **3** | Yes | No | Yes (no complications occurred) |  |
| **4** | Yes | No | No (complication occurred) |  |
| **5** | No | Not applicable | Not applicable | Not applicable |

**Table S4**: Study schedule

|  | **Screening** | **Enrolment** | **Intervention phase** | | | | | **In-Hospital Follow-Up** (applicable only if participant remains hospitalised) | | | **Close-out** |
| --- | --- | --- | --- | --- | --- | --- | --- | --- | --- | --- | --- |
| **Time point** | **0-72h before randomisation** | | **Day 1** | **Day 2** | **Day 3** | **Day 4** | **Day 5 (+1 day)** | **Day 7** | **Day 14** | **Discharge day** | **Day 90** |
| *S. aureus* positive blood culture | X |  |  |  |  |  |  |  |  |  |  |
| Identification of patient |  | X |  |  |  |  |  |  |  |  |  |
| Eligibility checks |  | X |  |  |  |  |  |  |  |  |  |
| Informed consent |  | X |  |  |  |  |  |  |  |  |  |
| Randomisation |  |  | X |  |  |  |  |  |  |  |  |
| **IMP administration** |  |  | **X** | **X** | **X** | **X** | **X** |  |  |  |  |
| **Time point** | **0-72h before randomisation** | | **Day 1 (-1 day)** | **Day 2** | **Day 3 (±1 day)** | **Day 4** | **Day 5 (±1 day)** | **Day 7 (±1 day)** | **Day 14  (±3 days)** | **Discharge day** | **Day 90 (±7 days)** |
| **Clinical** **assessments** | | | | | | | | | | | |
| Baseline data |  |  | X^1^ |  |  |  |  |  |  |  |  |
| SIRS criteria |  |  |  |  |  |  | X |  |  |  |  |
| All-cause mortality |  |  | Assessed until day 90 | | | | | | | | |
| Clinical failure |  |  |  |  |  |  |  |  |  | X | X |
| Length of hospital stay |  |  |  |  |  |  |  |  |  | X |  |
| Length of ICU stay |  |  |  |  |  |  |  |  |  | X | X |
| Antibiotic and/or surgical treatment |  |  |  |  |  |  |  |  |  |  | X |
| **Laboratory assessments** | | | | | | | | | | | |
| Blood cultures |  |  |  | X |  |  | X^2^ |  |  |  |  |
| Creatinine |  |  | X |  |  |  |  | X | X^3^ |  |  |
| CRP |  |  | X |  |  |  |  | X |  |  |  |
| ALT and gGT |  |  | X |  |  |  |  | X | X^3^ |  |  |
| Blood count (Hb, Tc, Lc) |  |  | X |  | X |  | X | X |  |  |  |
| Linezolid trough conc. |  |  |  |  |  |  | X^4^ |  |  |  |  |
| Microbiological failure |  |  |  |  |  |  |  |  |  | X | X |
| New antibiotic resistance |  |  |  |  |  |  |  |  |  |  | X |
| **Patient-reported outcomes** | | | | | | | | | | | |
| Quality of life (SF-36) |  |  |  |  |  |  |  |  |  |  | X |
| Return to usual level of function |  |  |  |  |  |  |  |  |  |  | X |
| **Safety outcomes** | | | | | | | | | | | |
| Serotonin toxicity, myelosuppression, hyperlactatemia^5^ |  |  | X | X | X | X | X | X |  |  |  |
| Acute kidney injury^5^ |  |  |  |  |  |  |  | X | X |  |  |
| *C. difficile*-associated diarrhoea^5^ |  |  |  |  |  |  |  | X |  |  | X |
| SAE / SAR |  |  | Collected until day 90 | | | | | | | | |

^1^ Baseline data include: date of hospital admission, index blood culture, linezolid susceptibility, ethnicity, vital signs, comorbidities, standard antibiotic treatment, markers of severity (mechanical ventilation, cardiac arrest, use of vasopressors, ICU), injection drug use, mental health diagnosis.
^2^ Assessment of persistent bacteraemia. Only required if day 2 blood culture is positive or recorded if done as part of standard of care.
^3^ Choose the result that represents the highest level recorded between day 6-14. ^4^ Linezolid trough concentration is only measured in a subset of participants in participating centres.
^5^ Adverse events of special interest until day 7 include: serotonin toxicity, myelosuppression, hyperlactatemia; until day 14: acute kidney injury; until day 90: C. difficile-associated diarrhoea.

Abbreviations: S. aureus – Staphylococcus aureus; SAE – Serious Adverse Event; SAR – Serious Adverse Reaction; SIRS - Systemic Inflammatory Response Syndrome; ICU – Intensive Care Unit; CRP – C-Reactive Protein; ALT – Alanine Transaminase; gGT – Gamma-glutamyl Transferase; Hb – Haemoglobin; Tc – Thrombocytes; Lc – Leukocytes; SF-36 – Short Form-36
