# Supplementary material for: Linezolid in addition to standard antibiotic treatment for Staphylococcus aureus bacteraemia: study protocol for a randomised, placebo-controlled trial
Source: BMJ Open. 2026 Apr 20;16(4):e118509. doi: 10.1136/bmjopen-2026-118509 (PMC13110666; doi:10.1136/bmjopen-2026-118509)
Supplement: online supplemental file 3 [file bmjopen-16-4-s003.pdf]

Dear Sir or Madam

Your relative is currently receiving medical care. As your relative is at present incapable of judgement and therefore unable to communicate their will to us independently, we are providing you with this information sheet on their behalf. They can be included in our clinical study or have already been included under a regulatory exception from informed consent in an emergency setting. We would like to inform you about our study and request your consent on behalf of your relative for their participation in the study, including retrospective consent if applicable.

If your relative has already been included in the study, a doctor who is not involved in the study has confirmed that they are suitable for participation in this study and that their interests will be safeguarded. In this case, we will also inform you which examinations and treatments have already been carried out on your relative as part of the study.

The clinical study is presented below. You can learn about the most important points in the brief summary.

Request to participate in medical research:

LIPS

Linezolid plus standard antibiotic treatment for blood infections with the bacterium *Staphylococcus aureus*

Information and declaration of consent

Dear Sir or Madam

We would like to ask whether you are willing to provide consent for the patient’s participation in the study. Participation is voluntary. All data collected in this project is subject to strict data protection regulations.
The research project is being carried out by the University Hospital Basel. If you are interested, we will be happy to inform you about the results of the research project.

We will explain the most important points to you and answer your questions. To give you an idea, here are the most important points. Detailed information will follow from page 5 onwards.

Why are we conducting this study?

- The bacterium *Staphylococcus aureus* (*S. aureus*), which is very frequently found on the skin or mucous membranes, has entered the patient's bloodstream.
- These infections in the blood are dangerous. In certain cases, standard antibiotic treatment only has an inadequate effect.
- In the case of a *S. aureus* blood infection, a suitable antibiotic is prescribed as standard therapy depending on the nature of the bacterium. In this study, we are investigating whether the additional administration of the antibiotic linezolid (study drug) has a positive effect on the course of the disease and whether the combination is well tolerated.
- The antibiotic linezolid can efficiently suppress the production of virulence factors. Bacterial virulence factors are like little tools that the bacterium *S. aureus* uses to cause disease in the body. These virulence factors help the bacteria to protect themselves from the body's defence system, to multiply and to damage the body.
- We believe that this leads to less damage to the body and can therefore have a positive influence on the course of the disease.

What is required of the patient during participation? - What happens during the study?

Form of participation: If you consent to the patient’s participation in the study, they will be randomly assigned to one of two groups.

- They belong either to the **group with the study drug (treatment group)** or to the **group with a placebo (control group)**. You, your relative and the doctors treating your relative will not know which group they belong to.

- In the treatment group, they receive linezolid (one tablet twice a day for 5 days) in addition to the usual antibiotic therapy.
- In the control group, they receive a placebo (one tablet twice a day for 5 days) in addition to the usual antibiotic therapy.

Duration: The study lasts 90 days. After their discharge from the hospital, no additional doctor's visits will be required specifically for the study.

- After 90 days, we will contact your relative by telephone (approx. 5-10 minutes) to enquire about their state of health.
- In addition, we will send them a questionnaire on their quality of life at this time, which we will ask them to complete (approx. 20 minutes).

Optional: In order to better understand *Staphylococcus aureus* in the blood, we would like to take additional blood samples (maximum of 1 additional blood tube per day). For this purpose, we will ask you for a separate consent on behalf of the patient at the end of this document. See **section 2.6** for more information on this optional part of the study.

What are the benefits and risks associated with participation?

The antibiotic linezolid has been authorised in Switzerland for over 20 years for the treatment of pneumonia or complicated skin and muscle infections. Linezolid is not yet routinely used as part of combination therapy.

Benefits

- If the additional administration of linezolid really is a better treatment, they could benefit directly if there are assigned to the treatment group
- If they are allocated to the control group, they will probably not benefit directly from participating in the study. However, it is possible that their participation will help future patients.

Risks

- Side effects may occur if they are also treated with linezolid. The known side effects of linezolid are generally of mild to moderate intensity (headache, gastrointestinal complaints), of limited duration or only occur after a longer treatment period.
- Caution is advised when certain medications such as antidepressants, migraine medications or opioids are administered at the same time as the study drug linezolid. In rare cases, serotonin syndrome may occur, which can manifest itself in confusion and rising blood pressure. Due to the patient's hospitalisation, medical monitoring is guaranteed at all times and the investigator can react promptly.

By signing at the end of the document, you attest that you consent voluntarily to the patient's participation in the study and that you have understood the contents of the entire document.

Detailed information on the study

1. Study aim and participant selection

In this information document, we refer to our research project as a *study*. If you, as a relative, consent to the patient's participation in the study, they are a study *participant.*

Before a test substance/medication is used to treat a disease, it must be tested in participants in a clinical study. Here, we want to investigate how effective and well tolerated the drug linezolid is for the treatment of *S. aureus* blood infections when it is given in addition to the usual antibiotic therapy.

Linezolid has been authorised in Switzerland for over 20 years for the treatment of pneumonia or complicated skin/muscle infections. Internationally, it is also used for *S. aureus* infections in the blood - usually as a follow-up therapy with tablets after treatment with another antibiotic. It has not yet been used as a combination therapy.

We are approaching you as the patient's relative, because any person with *Staphylococcus aureus* in their blood can take participate in the study.

2. General information

One third of all healthy adults carry the bacterium *Staphylococcus aureus* (*S. aureus*) on the skin or mucous membranes without any symptoms. The bacteria can enter the bloodstream through injuries or operations. Once the bacteria are in the bloodstream, they become very dangerous for the carrier. Unfortunately, treatment with standard antibiotics does not work for every patient. The infection can then become life-threatening. In addition, around one third of patients suffer permanent impairments after surviving this blood infection.

Other research studies have shown that so-called virulence factors may play an important role in the clinical outcomes after an infection with *S. aureus*. Virulence factors are like small tools that enable the *S. aureus* bacterium to survive and spread in the body and to defend itself against the human immune system.

There are a few antibiotics that specifically suppress the production of virulence factors in bacteria. Linezolid is one such antibiotic. In our study, we are investigating a new therapeutic approach in which the antibiotic linezolid is administered in addition to standard antibiotics in patients with *S. aureus* blood infections. The aim is to inhibit the bacterial production of virulence factors as quickly and efficiently as possible. In this way, we aim to minimise the damage caused by the bacteria in the body and ultimately significantly improve the response to treatment.

2.1 Structure of the study: How do we proceed?

In our study, participants are randomly assigned to one of two groups. This is important for obtaining reliable results from the study. This process is called randomisation. Each group receives a different treatment.

- **Group 1 (treatment group):** Half of the participants receive the antibiotic linezolid in addition to treatment with standard antibiotics. Linezolid is administered in the form of a tablet (dosage 600 mg). A total of 10 tablets are administered (one tablet twice a day for 5 days).
- **Group 2** (control group): Half of the participants receive a placebo in addition to treatment with standard antibiotics. The placebo looks like the linezolid tablet but contains no antibiotic. A total of 10 tablets are administered (one tablet twice a day for 5 days).

If the tablets cannot be swallowed, for example your relative has a feeding tube, the tablets can be crushed following clinical routine procedures.

The study is a so-called double-blind study. "Double-blind" means that neither the patients participating in the study, nor the investigator or other persons involved in the study (e.g. hospital staff) know who is in which group. In this sense, all participants are "blind". The aim is to take as little influence as possible on the study results. Randomisation and blinding allow us researchers to objectively assess how well the study drug linezolid works and whether there are any side effects.

We will conduct this study in accordance with the laws in Switzerland (Human Research Act, data protection laws). In addition, we will observe all internationally recognised guidelines. The responsible ethics committee and Swissmedic have already reviewed and authorised the study.

We plan to include a total of 606 patients with a *S. aureus* blood infection in the study. To achieve this, several hospitals in Switzerland are participating in this study.

A description of this study can also be found on the website of the Federal Office of Public Health at www.humanforschung-schweiz.ch under BASEC number 2025-00655.

3. Study conduct

If you are asked to provide consent for your relative's participation in this trial, they have already been admitted to a hospital. During their hospitalisation, most of the examinations and blood tests are part of routine care and will be carried out independently of their participation in the study. We planned the study together with patient representatives and took care to minimise the burden on study participants.

Schedule:

- On days 1-5, they receive the study medication twice a day and must take it in addition to the standard antibiotic treatment.
- The study-specific laboratory tests can be carried out on the blood samples that are also taken for routine treatment.
- We would like to measure the concentration of linezolid in the blood of the study participants on day 4 or 5. For this study-specific assessment, an additional tube containing 7.5 ml of blood will be taken as part of the blood collection on day 4 or 5.
- Your relative's hospital stay will not be prolonged due to taking part in the study. In the unlikely event that they are discharged before day 5, we will give them the study medication and an information sheet on taking the medication to take home. In this case, there will usually be a follow-up check with their doctor, which is independent of the study.
- They will not have any additional study-specific examinations until day 90.
- After 90 days, we will call the patient as part of the study and ask them a few questions. This will take about 5-10 minutes.
- We will also send them a questionnaire on their quality of life. Completing the questionnaire will take about 15-20 minutes. After the study results have been analysed, the patient's contact details will be deleted from the database.
- If the patient is no longer able to provide information or is repeatedly unavailable, the investigator may ask the patient's next of kin/emergency contact or family doctor about the state of the patient's health.

For the patient, participation in the study lasts 90 days and ends after the telephone interview and completion of the questionnaire on their quality of life

In very rare cases, we may have to exclude the patient from the study prematurely. This would be the case if they had serious side effects. The investigator would then discontinue the study medication early in the patient's best interest. In this unlikely scenario, they would still be included in the study and we would carry out the study-specific follow-up visits as planned. The patient's continued medical care is guaranteed at all times.

3.1 Optional additional samples

It is not yet known how *S. aureus* survives in the blood. It is possible that it hides in the white blood cells. To understand this even better, we want to analyse *S. aureus* directly in the blood. To do this, we need additional blood samples while *S. aureus* is suspected to be in the patient's blood (maximum of 1 additional tube with 7.5 ml of blood per day). These tests are not mandatory if they are taking part in the "LIPS study". You are free to decide whether you agree to these additional tests on behalf of the patient.

4. Benefit

- If the additional administration of linezolid really is a better treatment, they could benefit directly if they are assigned to the treatment group.
- If they are allocated to the control group, they will probably not benefit directly from participating in the study. However, it is possible that their participation will help future patients.

5. Voluntariness and obligations

Participation in the study is voluntary. If the patient does not participate or if you later wish to withdraw consent on the patient's behalf, no reasons need to be given. Medical care is guaranteed regardless of this decision.

As a participant, it is necessary that:

- The patient adheres to the instructions and requirements of the study throughout the duration of the study.
- The investigator is informed about the course of the disease and that new symptoms, new complaints and changes in well-being are reported.
- The investigator is informed about concurrent treatment and therapy by other physicians and about the intake of any medication.

6. Risks and burdens for the participant

There are risks and burdens associated with participating in this study, as with any medical treatment. Some risks are already known, others are still unknown. This uncertainty is not unusual in the context of studies. Many side effects can be treated medically. We will inform you of any new findings on risks and side effects during the trial. With any new combination of medications, it is possible that there are risks and side effects due to their interaction that we do not yet know.

6.1 The most frequent and most serious risks associated with the study drug

Here you will find information about the most common and most serious side effects that we are already aware of. We use the following descriptions:

very often	We find the side effect in more than 10 people out of 100 (more than 10%).
frequently	We find the side effect in 1 to 10 people out of 100 (1%-10%).
occasionally	We find the side effect in 1 to 10 people out of 1,000 (0.1%-1%).
rare	We find the side effect in 1 to 10 people out of 10,000 (0.01%-0.1%).
very rare	We find the side effect in less than 1 person in 10,000 (less than 0.01%).

Frequent side effects are:
The most common symptoms are diarrhoea, nausea, vomiting, cramps, flatulence and headaches as well as changes in taste (metallic taste), high blood sugar levels (hyperglycaemia), local fungal infections and altered liver values.

Occasional but potentially serious side effects include:

Treatment with linezolid may occasionally lead to changes in the blood count such as a lack of red or white blood cells or platelets. Symptoms of such blood count changes can include a drop in performance, tiredness, fever, chills, small haematomas, bleeding from the mucous membranes and impaired wound healing.

Rare to very rare but potentially serious side effects include:

- Lactic acidosis
- Serotonin syndrome
- Severe allergic (skin) reactions
- Seizures
- Muscle breakdown (rhabdomyolysis)

6.2 Pregnancy and breastfeeding

Controlled studies with linezolid in pregnant women are not available. Pregnant women are not excluded per se from participating in studies. In the case of pregnancy, the benefits for the pregnant participant must be balanced against the potential risk to the foetus.

As linezolid passes into breast milk, the patient must not breastfeed while taking it.

7. Alternative

Participation in the study is associated with opportunities and risks. Even if you do not consent to the patient's participation in this study, we will treat and care for them according to current medical standards.

8. Study Results

There are:

1. individual results of the study that directly affect the patient,
2. individual results of the study that arise by chance (so-called incidental findings)
3. objective results of the entire study.

Re 1: During the course of the study, the investigator will inform you on behalf of the patient about all new results and findings that are personally important for the participant. You will be informed verbally and in writing and can then decide again whether the patient should continue to participate in the study.

Re 2: Incidental findings are so-called "change findings", i.e. results that were not explicitly investigated but were found by chance. These may be, for example, the results of blood tests such as anaemia or iron deficiency. In the case of incidental findings, you will be informed if these findings are relevant to the health of the participant. This means that such findings will be communicated if a previously unknown disease is discovered by chance or if a disease that has not yet occurred can be prevented by taking preventive measures.

Re 3: We will also analyse the overall results of the study, which are based on the data from all study participants. These overall study results do not directly affect the patient and their health. At the end of the study (i.e. after the last data point has been collected from the last study participant), we will analyse the data to find out whether the addition of linezolid to the standard antibiotic treatment had a positive effect on fighting the blood infection. We do not expect the overall results of the study to be available before 2030. You will then find a summary of the results on www.humanforschung-schweiz.ch (BASEC No. 2025-00655). You can also email us at lips.trial@usb.ch at any time so that we can send you the overall results as soon as they are available.

9. Confidentiality of data and samples

We protect the patient's data (e.g. information such as blood pressure and pulse from the medical history) and their samples (e.g. blood samples). There are strict legal regulations in Switzerland for the protection of data and samples.

The Swiss Data Protection Act gives participants the right to information, correction and receipt of their data that is collected, processed and forwarded as part of the study.

9.1 Data processing and encoding

For this study, the patient's medical data is recorded and processed, partly in automated form. During data collection, the data is encoded. Encoding means that any information that could directly identify the patient (name, date of birth, etc.) is deleted and replaced by a code. For this purpose, there is a list (key list) that identifies each person with a unique code. Persons who do not have access to this key list cannot draw any conclusions about the study participants. The key list always remains in the hospital.

Only very few professionals will see the participants' unencoded data, and only to fulfil tasks within the scope of the study. These persons are subject to confidentiality. As a relative, you have the right to view the patient's data on their behalf.

9.2 Data protection and protection of samples

All data protection regulations are strictly adhered to. It is possible that the data must be transmitted in encrypted form, for example for publication, and can be made available to other researchers. If health-related data/samples are stored on site, this is a database/biobank for research purposes

Doctors who are responsible for follow-up treatment can be contacted to provide information on the state of health.

9.3 Data protection for further use

The data and samples from this study could be important for answering future research questions and/or could later be sent to and used in another database/biobank in Switzerland or abroad for other research projects. This other database/biobank must comply with the same standards as the database/biobank for this study.

For this further use, we ask you to sign another declaration of consent on the patient’s behalf at the end of this document. This second consent is independent of the patient's participation in this study.

9.4 Access rights during inspections

This study may be inspected by the responsible ethics committee and the medicines authority Swissmedic or audited by the sponsor who initiated the study. The investigator must then disclose the participants' data for review. Anyone involved in such audits or inspections is subject to confidentiality.

10. Consent withdrawal

The patient can withdraw from the study at any time and end their participation if they wish to do so or if you decide to do so on their behalf. In this case, the data and samples collected up to that point (e.g. blood values, blood cultures) will still be analysed in encoded form and remain part of the study data set. This is primarily for medical safety reasons. Please consider whether you agree to this before you consent to the study participation on the patient’s behalf.

11. Compensation

If the patient participates in this study, they will not receive any compensation.

12. Liability

The University Hospital Basel, which initiated the study and is responsible for its implementation, is liable for any harm that the patient may suffer in connection with the study drug linezolid and/or study related procedures. The requirements and process are regulated by law.

The University Hospital Basel has therefore taken out insurance with Helvetia Insurance, Dufourstrasse 40, 9001 St. Gallen to cover liability in the event of a claim.

The same liability regulations as for routine treatment apply to harm that is attributable to an authorised medicinal product used in accordance with medical standards or the use of a placebo or that would have occurred even if a standard therapy had been used.

If the patient suffers any harm as a result of participating in this study, please contact the investigator or the insurance company mentioned above.

13. Financing

This study is organised by the sponsor University Hospital Basel and is funded by the Swiss National Science Foundation (SNSF) (project number 221668).

14. Contact person

Your point of contact for this study is:

Name	PD Dr Richard Kühl
Address	Petersgraben 4, 4031 Basel
Telephone	+41 61 328 66 61<br>+41 61 265 25 25 (24-hour availability)
E-mail	richardalexander.kuehl@usb.ch

Declarations of consent

This consent consists of two independent declarations of consent

- Declaration of consent for participation in the study "Linezolid plus standard antibiotic treatment for blood infections with the bacterium *Staphylococcus aureus*" (LIPS study)
- Declaration of consent for the further use and disclosure of data and samples from this study in encoded form for further research.

Please read this form carefully. Please ask us if there is anything you do not understand or if there is anything else you would like to know. Your written consent is required for the patient's participation.

Declaration of consent for participation in the study "Linezolid plus standard antibiotic treatment for blood infections with the bacterium *Staphylococcus aureus*" (LIPS study)

**BASEC number**	2025-00655
**Title of the study**	"Linezolid plus standard antibiotic treatment for blood infections with the bacterium *Staphylococcus aureus*" (LIPS study)
**Responsible institution**<br>(Sponsor with address)	University Hospital Basel (USB)<br>Clinic for Infectiology, PD Dr Richard Kühl<br>Petersgraben 4<br>4031 Basel
**Study location**	University Hospital Basel
**Investigator at study location**	PD Dr Richard Kühl
**Participant:**<br>Name and first name in block capitals:<br><br>Date of birth:	

- As a relative or legal representative of the study participant (named above), I have been informed verbally and in writing by the signing investigator about the purpose and course of the study with linezolid and about possible advantages and disadvantages as well as possible risks.

- I confirm that I am giving consent to the patient's participation in the study and accept the verbal and written information on their behalf. I have had sufficient time to make this decision.
- My questions in connection with the patient's study participation have been answered. I will keep the written information and receive a copy of the written declaration of consent.
- I agree that their family doctor may be informed about the patient's participation in the study.
- In case of continuing treatment outside of the trial centre, I authorise the doctors providing follow-up treatment to transmit the data relevant to the trial to the investigator.
- I agree that the responsible experts of the sponsor, the responsible ethics committee and the medicines authority *Swissmedic* may inspect the patient's unencoded data for audit and monitoring purposes, but under strict confidentiality.
- In the case of results or incidental findings that directly affect the patient's health, I will be informed on their behalf.
- I am aware that the personal data and samples can only be passed on in encoded form for research purposes for this study, including abroad. The sponsor guarantees that data protection in accordance with Swiss standards will be observed.
- I can withdraw consent to participation in the study on behalf of the patient at any time and without giving reasons. The patient's continued medical care is guaranteed regardless of their participation in the study. The data and samples collected up to the time of consent withdrawal will still be analysed as part of the study.
- I have been informed that the University Hospital Basel has taken out insurance to cover any harm caused by the research project.
- I am aware that the instructions stated in the information leaflet must be complied with. The investigator may exclude the patient at any time to safeguard their health.

- I consent to the optional additional research on *S. aureus* in the patient's blood:
  Yes:☐ No: ☐

Confirmation by the next of kin/relative/legal representative:

I hereby confirm that the informed consent discussion has taken place and that the person lacking capacity has consented to participate in the study and/or that there are no signs of unwillingness to participate.

Place, date	Name and first name in capitals<br><br><br>Relationship to the patient:<br><br><br>Signature of the next of kin/relative/legal representative

Confirmation by the investigator: I hereby confirm that I have explained the nature, significance and scope of the study to the person signing above on behalf of the participant. I confirm that I will fulfil all obligations in connection with the study in accordance with Swiss law. If at any time I become aware of anything that could affect the participant's willingness to take part in the study, I will immediately inform the patient or the signing person on their behalf.

Place, date	Name and first name of the investigator in capitals<br><br><br>Signature of the investigator

Declaration of consent for the further use and/or disclosure of data and samples in encrypted form

**BASEC number**	2025-00655
**Title of the study**	"Linezolid plus standard antibiotic treatment for blood infections with the bacterium *Staphylococcus aureus*" (LIPS study)
**Participant:**<br>Name and first name in capitals:<br><br>Date of birth:	

- I authorise the patient's data from this study to be used for medical research and other purposes. The data will be stored in a database and used for future, as of yet undefined projects for an indefinite period of time. This consent remains valid until revoked.
- I understand that the data and samples are encoded and that the key is stored securely.
- The data can be analysed and stored in a database in Switzerland or abroad. The samples can be analysed here or abroad and stored in a biobank. Research institutions abroad must comply with the same data protection standards that apply in Switzerland.
- I voluntarily decide in favour of the further use and/or disclosure of data and samples of the patient in encoded form and can withdraw this decision at any time. I must only inform the patient's investigator and do not have to justify this decision.

  If I withdraw from the study on behalf of the patient, the patient's data will remain encoded.
- Usually, all data and samples are analysed together. If, by chance, a result emerges that is very important for the patient's health, I will be contacted on their behalf.

Place, date	Name and first name in capitals<br><br><br>Relationship to the patient:<br><br><br>Signature of the next of kin/relative/legal representative

Confirmation by the investigator: I confirm that I have explained to the patient's next of kin or legal representative the nature, significance and implications of the further use and/or disclosure of samples and/or data.

Place, date	Name and first name of the investigator in capitals<br><br><br>Signature of the investigator
