# Supplementary material for: Linezolid in addition to standard antibiotic treatment for Staphylococcus aureus bacteraemia: study protocol for a randomised, placebo-controlled trial
Source: BMJ Open. 2026 Apr 20;16(4):e118509. doi: 10.1136/bmjopen-2026-118509 (PMC13110666; doi:10.1136/bmjopen-2026-118509)
Supplement: online supplemental file 4 [file bmjopen-16-4-s004.pdf]

Request to participate in medical research:

LIPS

Linezolid plus standard antibiotic therapy for blood infections with the bacterium *Staphylococcus aureus*

Information and declaration of consent

Dear Sir or Madam

We would like to ask you retrospectively whether you would be willing to participate in our research project "LIPS Study". An independent doctor has confirmed in the emergency situation that you are suitable for participation in this study and that your interests have been safeguarded, and/or your relative or legal representative has consented to your participation in this study on your behalf during the period of your acute illness.

Your participation is **voluntary**. All data and samples collected in this project are subject to strict data protection regulations.

The following **patient information** is intended to help you decide whether you would like to continue participating in the study. In a personal discussion, the investigator will explain the most important points to you and answer your questions. This is what we call the doctors who are responsible for a study and who will look after you as part of this study. If you would like to participate, please sign the **consent form** at the end. With your signature you confirm that you have read and understood the patient information. If there is anything you do not understand, please ask the investigator.

In our study, we want to test a new combination of already approved antibiotics to find out whether this combination works as well or better than the current standard therapy for *Staphylococcus aureus* blood infections. We call such research a **clinical trial.** This study is initiated, supervised and managed by the University Hospital Basel, which is therefore referred to as the "sponsor".

Your point of contact for this study is:

Name	PD Dr Richard Kühl
Address	Petersgraben 4, 4031 Basel
Telephone	+41 61 328 66 61
	+41 61 265 25 25 (24-hour availability)
E-mail	richardalexander.kuehl@usb.ch

The most important information can be found directly below, detailed information follows from page 4 onwards.

Why are we conducting this study?

- The bacterium *Staphylococcus aureus* (*S. aureus*), which is very common on the skin or mucous membranes, has entered your bloodstream.
- These infections in the blood are dangerous. In certain cases, standard antibiotic treatment only has an inadequate effect.
- In the case of a *S. aureus* blood infection, a suitable antibiotic is prescribed as standard therapy depending on the nature of the bacterium. In this study, we are investigating whether the additional administration of the antibiotic linezolid (study drug) has a positive effect on the course of the disease and whether the combination is well tolerated.
- The antibiotic linezolid can efficiently suppress the production of virulence factors. Bacterial virulence factors are like little tools that the bacterium *S. aureus* uses to cause disease in the body. These virulence factors help the bacteria to protect themselves from the body's defence system, to multiply and to damage the body.
- We believe that this leads to less damage to the body and can therefore have a positive influence on the course of the disease.

You can find out more about the scientific background to the study in **Chapter 1**.

What do you have to do if you take part?

You have already been assigned to one of 2 groups during the period of your acute illness. The selection was random.

- You belong either to the **group with the study drug (treatment group)** or to the **group with a placebo (control group)**. You and the doctors treating you do not know which group you belong to.
- In the treatment group, you have received/will receive linezolid (one tablet twice a day for 5 days) in addition to the usual antibiotic therapy.
- In the control group, you have received/will receive a placebo (one tablet twice a day for 5 days) in addition to the usual antibiotic therapy.

Duration: The study lasts 90 days for you. After your discharge from the hospital, no additional doctor’s visits will be required specifically for the study.

- After 90 days, we will contact you by telephone (approx. 5-10 minutes) to enquire about your state of health.
- At this time, we will also send you a questionnaire on your quality of life, which we will ask you to complete (approx. 20 minutes). When giving your consent, please tick whether you would like to receive the questionnaire electronically by e-mail or SMS, or on paper by post.

You can find out more about the process and procedure of the study in **Chapter 2**.

Optional: To better understand *Staphylococcus aureus* in the blood, we would like to take further blood samples (maximum of 1 additional blood tube per day). We will ask for your separate consent at the end of this document. See **section 2.6** for more information on this optional part of the study.

What are the benefits and risks associated with participation?

The antibiotic linezolid has been authorised in Switzerland for over 20 years for the treatment of pneumonia or complicated skin and muscle infections. Linezolid is not yet routinely used as part of combination therapy.

Benefits

- If the additional administration of linezolid really is a better treatment option, you could benefit directly if you were assigned to the treatment group.
- If you were allocated to the control group, you will probably not benefit directly from your participation in the study. However, it is possible that your participation will help future patients.

Risks

- Side effects may occur if linezolid is added on top of your background medication. The known side effects of linezolid are generally of mild to moderate intensity (headache, gastrointestinal complaints), of limited duration, or only occur after a longer treatment period.
- Caution is advised when certain medications such as antidepressants, migraine medications or opioids are administered at the same time as the study drug linezolid. In rare cases, serotonin syndrome may occur, which can manifest itself in confusion and rising blood pressure. Due to your hospitalisation, medical monitoring is guaranteed at all times and the investigator can react promptly.

Chapter 3 contains more detailed information on other risks and burdens.

Detailed information on the study

1. The scientific background of the study

1.1 Background: Why are we conducting this study?

One third of all healthy adults carry the bacterium *Staphylococcus aureus* (*S. aureus*) on the skin or mucous membranes without any symptoms. The bacteria can enter the bloodstream through injuries or operations. Once the bacteria are in the bloodstream, they become very dangerous for the carrier. Unfortunately, treatment with standard antibiotics does not work for every patient. The infection can then become life-threatening. In addition, around one third of patients suffer permanent impairments after surviving this blood infection.

Other research studies have shown that so-called virulence factors may play an important role in the clinical outcomes after an infection with *S. aureus*. Virulence factors are like small tools that enable the *S. aureus* bacterium to survive and spread in the body and to defend itself against the human immune system.

There are a few antibiotics that specifically suppress the production of virulence factors in bacteria. Linezolid is one such antibiotic. In our study, we are investigating a new therapeutic approach in which the antibiotic linezolid is administered in addition to standard antibiotics in patients with *S. aureus* blood infections. The aim is to inhibit the bacterial production of virulence factors as quickly and efficiently as possible. In this way, we aim to minimise the damage caused by the bacteria in the body and ultimately significantly improve the response to treatment.

Linezolid has been authorised in Switzerland for over 20 years for the treatment of pneumonia or complicated skin/muscle infections. Internationally, it is also used for *S. aureus* infections in the blood - usually as a follow-up therapy with tablets after treatment with another antibiotic. It has not yet been used as a combination therapy. Linezolid has been declared a back-up antibiotic in Switzerland. Due to the small number of multi-resistant bacteria in Switzerland, linezolid is rarely used here.

1.2 Structure of the study: How do we proceed?

In our study, participants are randomly assigned to one of two groups. This is important for obtaining reliable results from the study. This process is called randomisation. Each group receives a different treatment.

- **Group 1** (treatment group): Half of the participants receive the antibiotic linezolid in addition to treatment with standard antibiotics. Linezolid is administered in the form of a tablet (dosage 600 mg). A total of 10 tablets are administered (one tablet twice a day for 5 days).
- **Group 2** (control group): Half of the participants receive a placebo in addition to treatment with standard antibiotics. The placebo tablet looks like the linezolid tablet but contains no antibiotic. A total of 10 tablets are administered (one tablet twice a day for 5 days).

If the tablets cannot be swallowed, for example if you have a feeding tube, the tablets can be crushed following clinical routine procedures.

The study is a so-called double-blind study. "Double-blind" means that neither the patients participating in the study, nor the investigator or other persons involved in the study (e.g. hospital staff) know who is in which group. In this sense, all participants are "blind". The aim is to take as little influence as possible on the study results. Randomisation and blinding allow us researchers to objectively assess how well the study drug linezolid works and whether there are any side effects.

1.3 Regulations on scientific research involving human subjects

We will conduct this study in accordance with the laws in Switzerland (Human Research Act, data protection laws). In addition, we will observe all internationally recognised guidelines. The responsible ethics committee and Swissmedic have already reviewed and authorised the study.

We plan to include a total of 606 patients with a *S. aureus* blood infection in the study. To achieve this, several hospitals in Switzerland are participating in this study.

A description of this study can also be found on the website of the Federal Office of Public Health www.humanforschung-schweiz.ch under BASEC number 2025-00655.

2. Study conduct

2.1 What do you have to do if you take part in the study?

Participation in the study is voluntary and lasts 90 days. You must adhere to the schedule (→ **Chapter 2.2**) and the instructions that your investigator will discuss with you.

You must inform your investigator,

- if your state of health deteriorates significantly or if you have new health problems.
- if you wish to terminate the study prematurely (→ **Chapter 2.3** and **2.4**).

2.2 What happens during the appointments?

You have already received study-specific treatment. Your investigator will explain to you which treatment and examinations you have already received and which are still to come. While you are hospitalised, most of the examinations and blood tests are part of your general treatment and will be carried out independently of your participation in the study. We planned the study together with patient representatives and have taken care to minimise the burden on study participants.

Schedule:

- On days 1-5, you have received/will receive the study medication twice a day, which you must take in addition to the standard antibiotic treatment.
- The study-specific measurements can be carried out on the blood samples that are also taken for your routine treatment.
- We would like to measure the concentration of linezolid in the participants' blood on day 4 or 5. For this study-specific measurement, an additional tube containing 7.5 ml of blood will be taken as part of the blood collection on day 4 or 5.
- You do not have to stay in hospital any longer to take part in the study. In the unlikely event that you are discharged before day 5, we will give you the study medication and an information sheet on taking the medication to take home with you. In this case, there will usually be a follow-up check, which is independent of the study
- You will not have any additional study-specific examinations until day 90.
- After 90 days, we will call you as part of the study and ask you a few questions. This will take about 5-10 minutes.
- We will also send you a questionnaire on your quality of life. Please tick the box on the consent form to indicate how you would like to receive the questionnaire (e-mail, SMS, post). Completing the questionnaire will take about 15-20 minutes. After the study results have been analysed, your contact details will be deleted from the database.
- If you are no longer able to provide information yourself or are repeatedly unavailable, the investigator may ask your next of kin/emergency contact or your family doctor about the state of your health.

2.3 When does participation in the study end?

Your participation lasts 90 days and ends after the telephone survey and completion of the quality-of-life questionnaire. You can end your participation at any time, even earlier. You do not have to explain why you no longer wish to participate. If you would like to end your participation earlier, please speak to your investigator.

If you end your participation prematurely, this will not affect your further medical care and treatment (→ **Chapter 2.4** for alternative treatment options).

If you discontinue the study earlier, we ask you to continue to inform your investigator if your state of health deteriorates significantly or if you have new symptoms. We will continue to analyse the data and samples (e.g. blood values, blood cultures) collected up to that point for the study. Your study data and samples will remain encrypted (→ **Chapter 6**).

In very rare cases, we may have to ask you to discontinue the study medication early. This would be the case if you had serious side effects. Your investigator would then stop the study medication early in your best interest. In this unlikely scenario, however, you would still be included in the study and we would carry out the study-specific follow-up visits as planned.

2.4 What happens if you don't want to take part?

Acute treatment as part of the study may already be in the past. Even if you are not participating in this study, we will provide you with the best possible medical treatment and care in accordance with current standards.

2.5 Pregnancy and breastfeeding

Controlled studies with linezolid in pregnant women are not available. Pregnant women are not excluded per se from participating in this study. In the case of pregnancy, the benefits for the pregnant participant must be balanced against the potential risk to the foetus.

As linezolid passes into breast milk, you must not breastfeed while taking this medicine.

2.6 Optional additional examinations

It is not yet known how *S. aureus* survives in the blood. It is possible that it hides in the white blood cells. To understand this even better, we want to analyse *S. aureus* directly in the blood. To do this, we need additional blood samples while *S. aureus* is suspected to be in your blood (maximum of 1 additional tube with 7.5 ml of blood per day). These tests are not mandatory if you are participating in the LIPS study. You are free to decide whether you agree to these additional tests.

3. Risks, burdens and side effects

3.1 What risks and burdens can occur?

There are risks and burdens associated with participating in this study, as with any medical treatment. Some risks are already known, others are still unknown. This uncertainty is not unusual in the context of studies. You will find a list of the most common and most serious risks in **Chapter 3.2.** Many side effects can be treated medically. We will inform you of any new findings on risks and side effects during the trial. With any new combination of medications, it is possible that there are risks and side effects due to their interaction that we do not yet know.

In addition, there are risks associated with the medical examinations that we carry out in this study. You will already be familiar with some of the examinations. You will find a list of these risks in **Chapter 3.3**.

3.2 The most frequent and most serious risks associated with the study drug

Here you will find information about the most common and most serious side effects that we are already aware of. We use the following descriptions:

very often	We find the side effect in more than 10 people out of 100 (more than 10%).
frequently	We find the side effect in 1 to 10 people out of 100 (1%-10%).
occasionally	We find the side effect in 1 to 10 people out of 1,000 (0.1%-1%).
rare	We find the side effect in 1 to 10 people out of 10,000 (0.01%-0.1%).
very rare	We find the side effect in less than 1 person in 10,000 (less than 0.01%).

Common side effects are

The most common symptoms are diarrhoea, nausea, vomiting, cramps, flatulence and headaches, as well as changes in taste (metallic taste), high blood sugar levels (hyperglycaemia), local fungal infections and altered liver values.

Occasional but potentially serious side effects include

Treatment with linezolid may occasionally lead to changes in the blood count, such as a lack of red or white blood cells or platelets. Symptoms of such blood count changes can include a drop in performance, tiredness, fever, chills, small haematomas, bleeding from the mucous membranes and impaired wound healing.

Rare to very rare but potentially serious side effects include

- Lactic acidosis
- Serotonin syndrome
- Severe allergic (skin) reactions
- Seizures
- Muscle breakdown (rhabdomyolysis)

3.3 Risks and burdens from examinations in the study

As already described, the vast majority of the tests carried out are part of your standard treatment for a *S. aureus* infection in the blood (→ **Chapter 2.2**). On day 4 or 5, we would like to measure the concentration of linezolid in the patient's blood. For this study-specific measurement, an additional tube containing 7.5 ml of blood will be taken as part of the blood collection.

Bruising, bleeding or swelling at the puncture site may occur during blood sampling.

4. Financing and compensation

This study is organised by the sponsor University Hospital Basel and is funded by the Swiss National Science Foundation (SNSF) (project number 221668).

If you take part in this study, you will not receive any money or other compensation. There are no additional costs for you or your health insurance company for participating in the study

5. Study results

As most of the study is carried out as part of your usual treatment, you will be informed of the results by your attending physician. There may also be incidental findings. Incidental findings are "chance findings" that are not intended. These can be, for example, the results of your blood tests showing you have anaemia or an iron deficiency. We will inform you if we diagnose incidental findings that are relevant your health.

For example, we will inform you if we happen to discover a disease that you do not yet know about and that we can treat. If you *do not* wish to be informed, please discuss this with your investigator. Some results and incidental findings are always communicated, for example, if other people are at risk or if it must be reported by law.

We will also analyse the overall results of the study, which are based on the data of all study participants. These overall study results will not directly affect you or your health. After the end of the study (i.e. after the last data point has been collected from the last study participant), we will analyse the data to find out whether the addition of linezolid to the standard antibiotic treatment had a positive effect on fighting the blood infection. We do not expect the overall results of the study to be available before 2030. You will then find a summary of the results on www.humanforschung-schweiz.ch under BASEC number 2025-00655. You can also email us at lips.trial@usb.ch at any time so we can send you the overall results once they are available.

6. Protection of data and samples

We protect your data (e.g. information such as blood pressure and heart rate from your medical history) and your samples (e.g. your blood samples). There are strict legal regulations in Switzerland for the protection of data and samples.

The Swiss Data Protection Act gives you the right to information, correction and receipt of your data that is collected, processed and forwarded as part of the study.

6.1 Encoding of data and samples

Each study generates data from the assessments (e.g. blood values, blood cultures). In addition to health data, demographic information is also collected. This data is documented and

recorded electronically in large tables, the so-called "data collection forms". All data is documented in encoded form. "Encoded" means that personal information that could directly identify you is stored *separately* from the examination results. For example, your name, date of birth or place of residence are *not* included in the data collection form. For this purpose, there is a list (key list) that identifies each person with a unique code. This key list remains at the hospital for a period of 20 years and is then destroyed. No one else will receive this key list. Special exceptions are regulated in **chapter 6.5.** Your contact details for sending the quality-of-life questionnaire and the telephone call will be deleted from the study database after the study results have been analysed.

If we pass on study data for the purpose of further analyses, e.g. to the sponsor or other researchers, the data is always passed on in encoded form and your personal data is protected. This also applies if the data is passed on to research institutes outside Switzerland.

6.2 Safe handling of data and samples during the study

The sponsor is responsible for the safe handling of your data and samples from this study. The sponsor is responsible for ensuring that the applicable laws, e.g. data protection laws, are complied with. This also applies if (encoded) data or samples for analyses are sent to countries where data protection laws are less favourable. This is how the sponsor of this study protects your data:

In this study, your data is recorded, transmitted and collected in an electronic database. The data is stored on servers in Switzerland. Only authorised study leaders, investigators, data managers and, if applicable, local authorities have access to the data during and after the study.

It is often important that your general practitioner shares your medical history with the investigator. This also applies to other doctors who treat you. You authorise this by giving your consent at the end of the document

6.3 Safe handling of data and samples after the end of the study

The sponsor remains responsible for the safe handling of your data and samples even after the end of the study. The law stipulates that all study documents, e.g. the data collection forms, must be kept for at least 20 years.

After the end of this long period, study data remain encoded. Health-relevant data from your medical history, including from this study, are and will always remain accessible to your treating healthcare professionals.

For some of the patients, we will isolate the *S. aureus* bacteria from the blood cultures and grow them in the laboratory. This will help us to understand the differences between the various strains of *S. aureus* and allow us to analyse their genetic, protein-related and metabolic characteristics. These samples let us to carry out important basic research into *S. aureus* infections. The samples do not contain any genetic data from you personally. It will not be possible to draw any conclusions about your person from the isolated bacteria.

If there are leftovers of the samples (e.g. blood samples, bacteria) at the end of the study, we collect and store them in a secure location in encoded form. This may allow them to be used later for further analyses (→ **Chapter 6.4**). Such a collection of encoded samples is called a "biobank". There are strict rules for biobanks to ensure that the information from your samples remains well protected.

Once a study has been completed, its results are usually published in scientific journals. For this purpose, the study results are summarised in a manuscript and reviewed by other experts. Your encoded data may be forwarded to these experts. However, your separate consent would be required for the data to be used for new research purposes.

6.4 Further use and disclosure of your data and samples in other, future studies

Your data and samples from this study are very important for future research. Samples that have not been used up for this study as well as data that have been collected for this study can potentially be reused and/or passed on for other studies (including abroad).

We require your separate consent for the further use and/or forwarding of your data and samples. This is voluntary. Please read the additional declaration of consent at the end of the document carefully. Please sign the consent form if you wish to support further research with your data and samples in the future. Even if you do not consent, you can still take part in the study.

6.5 Access rights during inspections

The execution of this study may be inspected or audited. Inspections are carried out by authorities such as the responsible ethics committee or the licensing authority *Swissmedic*. The sponsor must also carry out audits to ensure the quality of the study and the results.

A small number of specially trained people are given access to your personal data and medical history for this purpose. The data is therefore *not* encoded for the audits and inspections. The people who see your unencoded data are subject to confidentiality.

As a study participant, you have the right to view your data at any time.

7. Insurance cover

You are insured if you suffer harm as a result of taking the study drug. The process is regulated by law. For this purpose, the sponsor has taken out an insurance policy with Helvetia Insurance, Dufourstrasse 40, 9001 St. Gallen. If you believe that you have been harmed because of the study, please contact your investigator or the insurance company directly.

The same liability regulations as for routine treatment apply to harm that is attributable to an authorised medicinal product used in accordance with medical standards or that would have occurred even if a standard therapy had been used. In such cases, the hospital's liability insurance will cover the costs/compensation.

Declarations of consent

This consent consists of two independent declarations of consent:

- Declaration of consent for participation in the study "Linezolid plus standard antibiotic treatment for blood infections with the bacterium *Staphylococcus aureus*" (LIPS study)
- Declaration of consent for the further use and disclosure of data and samples from this study in encoded form for further research.

Please read this form carefully. Please ask us if there is anything you do not understand or if there is anything else you would like to know. Your written consent is required for participation.

Declaration of consent for participation in the study "Linezolid plus standard antibiotic treatment for blood infections with the bacterium *Staphylococcus aureus*" (LIPS study)

**BASEC number**	2025-00655
**Title of the study**	"Linezolid plus standard antibiotic treatment for blood infections with the bacterium *Staphylococcus aureus*" (LIPS study)
**Responsible institution** (Sponsor with address)	University Hospital Basel (USB)<br>Clinic for Infectiology, PD Dr Richard Kühl<br>Petersgraben 4<br>4031 Basel
**Study location**	University Hospital Basel
**Investigator at study location**	PD Dr Richard Kühl
**Participant:**<br>Surname and first name in block capitals:<br><br>Date of birth:	

- I have received verbal and written information about the study from the signing investigator.
- The investigator has explained the purpose, course and risks of the study to me.
- I take part in the study voluntarily.

- The investigator explained to me what standard treatments are available outside of the study.
- I had sufficient time to make this decision. I will keep the written information and receive a copy of my written declaration of consent.
- I can cancel my participation at any time. I do not have to explain why. Even if I end my participation, I will continue to receive medical treatment. The data and samples that have been collected up to that point will still be analysed as part of the study.
- If it is better for my health, the investigator can exclude me from the study at any time.
- I understand that my data and samples will only be passed on in encoded form for this study. The sponsor will ensure that data protection in accordance with Swiss standards is observed (even if the data is transferred abroad).
- I will be informed of any results and/or incidental findings that directly affect my health. If I do not wish to be informed, I will discuss this with my investigator.
- My general practitioner may know that I am taking part in the study and may share data from my medical history that is important for the study with the investigator. This also applies to other doctors who treat me.
- The investigator may ask my next of kin/emergency contact about the state of my health if I am no longer able to provide information myself or if I am repeatedly unavailable.
- The responsible specialists of the sponsor, the ethics committee and the medicines licensing authority *Swissmedic* may view my unencoded data for inspection. All these persons are subject to confidentiality.
- I know that the University Hospital Basel has taken out insurance. This insurance pays out if I suffer damage - but only if the damage is directly related to the study.

- I would like to receive the questionnaire on quality of life in this way:

  ☐ E-mail
  If yes, please enter your e-mail address here: ____________________

  ☐ SMS
  if yes, please enter your mobile phone number here: ____________________

  ☐ Letter

- I consent to the optional additional tests for *S. aureus* in my blood:
  Yes:☐ No:☐

Place, date	Name and first name of the participant in capitals<br><br><br>Signature of participant

Confirmation by the investigator: I hereby confirm that I have explained the nature, significance, and scope of the study to this participant. I confirm that I will fulfil all obligations in connection with this study under Swiss law. If at any time I become aware of anything that could affect the participant's willingness to take part in the study, I will inform them immediately.

Place, date	Surname and first name of the investigator in capitals<br><br><br>Signature of the investigator

Declaration of consent for the further use and/or disclosure of data and samples in encoded form

This consent does not concern you in the sense of your personal participation in the study (→·**Chapter 6.4** of the patient information).

"Further use" means that data and samples can be stored beyond the time of your participation in the study and used in encoded form for further research. This can mean, for example, that a blood sample and corresponding laboratory values from you are statistically analysed together with many other values, or that new research is carried out with them.

"Sharing" means that your data and samples may be passed on to other researchers or research institutions in encoded form for further research projects. These other researchers or research institutions may also be located abroad. It is the responsibility of the sponsor to ensure that this country has an adequate level of data protection comparable to that in Switzerland.

**BASEC number**	2025-00655
**Title of the study**	"Linezolid plus standard antibiotic treatment for blood infections with the bacterium *Staphylococcus aureus*" (LIPS study)
**Participant:**<br>Name and first name in capitals:<br><br><br>Date of birth:	

- I authorise that my encoded data and samples from this study may be used and passed on for medical research (also abroad). The samples will be stored in a biobank. They will then be available for future research projects for an indefinite period of time.
- I understand that the data and samples are encoded and that the key is stored securely.
- The data can be analysed and stored in a database in Switzerland or abroad. The samples can be analysed here or abroad and stored in a biobank. Research institutions abroad must comply with the same data protection standards that apply in Switzerland.
- I voluntarily decide in favour of the further use and/or disclosure of data and samples in encoded form. I can withdraw this consent at any time and will only need to inform the investigator without having to justify this decision.

  If I withdraw from the study, the data remains encoded.

- Usually, all data and samples are analysed together. If, by chance, there is a finding that is very important for my health, I will be contacted. If I do not wish to be contacted, I will inform the investigator.

Place, date	Name and first name of participant in capitals<br><br><br>Signature of participant

Confirmation by the investigator: I confirm that I have explained to the participant the nature, significance and implications of the further use and/or disclosure of samples and/or data.

Place, date	Name and first name of the investigator in capitals<br><br><br>Signature of the investigator
