# Supplementary material for: Linezolid in addition to standard antibiotic treatment for Staphylococcus aureus bacteraemia: study protocol for a randomised, placebo-controlled trial
Source: BMJ Open. 2026 Apr 20;16(4):e118509. doi: 10.1136/bmjopen-2026-118509 (PMC13110666; doi:10.1136/bmjopen-2026-118509)
Supplement: online supplemental file 5 [file bmjopen-16-4-s005.pdf]

Docusign Envelope ID: 7FB1D507-0860-4709-95DA-14CC8893F28B

Clinical Trial protocol

Combination antibiotic treatment with linezolid for Staphylococcus aureus bacteraemia: a randomised controlled trial

Short title: **Li**nezolid **p**lus **s**tandard of care
Trial acronym: **LIPS**

Study Type:	Clinical trial with Investigational Medicinal Product (IMP)
Study Categorisation:	B
Study Registration:	humanresearch-switzerland.ch, clinicaltrials.gov (NCT06958835)
Study Identifier:	LIPS Trial
Sponsor-Investigator:	PD Dr. Richard Kühl<br>Department of Infectious Diseases<br>University Hospital Basel<br>Petersgraben 4<br>4031 Basel<br>Switzerland<br>Phone: +41 (0)61 328 66 61<br>Email: richardalexander.kuehl@usb.ch
Investigational medicinal product:	Linezolid 600 mg tablet<br>Placebo tablet
Protocol Version and Date:	Version 1.2 (dated 30/07/2025)

Confidentiality:
The information contained in this document is explicitly not confidential. We will make the study protocol publicly available as a PDF file on clinicaltrials.gov.

LIST OF PROJECT LEADERS, LOCAL INVESTIGATORS AND OTHER KEY PERSONS INVOLVED IN THE TRIAL

Please refer to the separate document "LIPS list of project leaders" for detailed information on project leaders, local investigators and other key persons involved in the trial including their roles and functions.

LIPS

Signature Pages

Study number	•	ClinicalTrials.gov Identifier: NCT06958835
Study Title	•	Combination antibiotic treatment with linezolid for Staphylococcus aureus bacteraemia: a randomised controlled trial (LIPS Trial)

The Sponsor-Investigator and trial statistician have approved the protocol version 1.2 dated 30/07/2025 and confirm hereby to conduct the study according to the protocol, current version of the World Medical Association Declaration of Helsinki, the ICH-GCP guidelines and the local legally applicable requirements.

The Sponsor-Investigator has received the ICF and considers it appropriate for use.

Sponsor-Investigator: PD Dr. Richard Kühl

Basel 31.07.2025 31.07.2025 | 17:11 MESZ — Signiert von: Richard Kühl 6CCB4A1527DC4A8...

Place/Date — Signature

Co-Principal Investigator: PD Benjamin Speich, PhD

31.7.2025, Basel 31.07.2025 | 15:59 MESZ — DocuSigned by: Benjamin Speich E0B0625F2CE44F5...

Place/Date — Signature

Co-Principal Investigator: Prof Dr. Nina Khanna

Basel 31.07.2025 | 16:19 MESZ — DocuSigned by: Nina Khanna 91E7D0E177DC4DA...

Place/Date — Signature

Trial Statistician: Laura Werlen, PhD

Basel 04.08.2025 | 16:49 MESZ — Signiert von: Laura Werlen 84DEFFDBE5794DE...

Place/Date — Signature

Trial Manager: Natalie Rose, PhD

Basel 31.07.2025 | 16:02 CEST — Signiert von: Natalie Rose F0466B05A8EF463...

Place/Date — Signature

Local Investigator at study site:

I have read and understood this trial protocol and agree to conduct the trial as set out in this study protocol, the current version of the World Medical Association Declaration of Helsinki, ICH-GCP guidelines and the local legally applicable requirements.

I have received the ICF and consider it appropriate for use.

Site:

Local investigator:

Place/Date	Signature

Table of Contents

STUDY SYNOPSIS **8**
ABBREVIATIONS **13**
SUMMARY OF THE REVISION HISTORY IN CASE OF AMENDMENTS **15**
STUDY SCHEDULE **16**
1. STUDY ADMINISTRATIVE STRUCTURE **17**
1.1 Monitoring institution 17
1.2 Data Safety Monitoring Board 17
1.3 Independent outcome assessment board 17
1.4 Any other relevant Committee, Person, Organisation, Institution 17
1.4.1 Patient and Public Involvement (PPI) 17
1.4.2 IMP Manufacturer 17
2. ETHICAL AND REGULATORY ASPECTS **18**
2.1 Study registration 18
2.2 Categorisation of study 18
2.3 Competent Ethics Committee (CEC) 18
2.4 Swissmedic 18
2.5 Ethical Conduct of the Study 18
2.6 Declaration of interest 18
2.7 Patient Information and Informed Consent 19
2.8 Participant privacy and confidentiality 19
2.9 Premature termination of the study 19
2.10 Protocol amendments 20
3. BACKGROUND AND RATIONALE **21**
3.1 Background and Rationale 21
3.2 Investigational Medicinal Product (treatment) and Indication 22
3.3 Preclinical Evidence 22
3.4 Clinical Evidence to Date 23
3.5 Rationale for the dosage, route, regimen 23
3.6 Explanation for choice of comparator 23
3.7 Risks / Benefits 24
3.8 Justification of choice of study population 24
3.9 Summary of Background and Data on Linezolid 25
4. STUDY OBJECTIVES **26**
4.1 Overall Objective 26
4.2 Primary Objective 26
4.3 Secondary Objectives 26
4.4 Safety Objectives 26
5. STUDY OUTCOMES **27**
5.1 Primary Outcome 27
5.2 Secondary Outcomes 28
5.3 Other Outcomes of Interest 29
5.4 Safety Outcomes 29
6. STUDY DESIGN **30**
6.1 General study design and justification of design 30
6.2 Patient and Public Involvement 30
6.2.1 PPI activities before receiving approval from authorities 30
6.2.2 PPI activities after receiving approval from authorities 31
6.3 Methods of minimising bias 31

6.3.1 Randomisation 31
6.3.2 Blinding procedures....31
6.3.3 Other methods of minimising bias 32
6.4 Unblinding Procedures (Code break) 32
7. STUDY POPULATION 33
7.1 Eligibility criteria....33
7.2 Recruitment and screening 33
7.3 Assignment to study groups 34
7.4 Criteria for withdrawal / discontinuation of participants....34
8. STUDY INTERVENTION.... 36
8.1 Identity of Investigational Medicinal Products....36
8.1.1 Experimental Intervention....36
8.1.2 Control Intervention (standard/routine/comparator treatment)....36
8.1.3 Packaging, Labelling and Supply (re-supply) 36
8.1.4 Storage Conditions....36
8.2 Administration of experimental and control interventions 36
8.2.1 Experimental Intervention....36
8.2.2 Control Intervention 36
8.3 Dose modifications 36
8.4 Compliance with study intervention 37
8.5 Data Collection and Follow-up for withdrawn participants 37
8.6 Trial specific preventive measures 37
8.7 Concomitant Interventions (treatments) 37
8.8 Study Drug Accountability 37
8.9 Return or Destruction of Study Drug 37
9. STUDY ASSESSMENTS 38
9.1 Study flow chart/ table of study procedures and assessments 38
9.2 Assessments of outcomes 39
9.2.1 Assessment of primary outcome 39
9.2.2 Assessment of secondary outcomes 40
9.2.3 Assessment of other outcomes of interest in a subset of participants 41
9.2.4 Assessment of safety outcomes....41
9.2.5 Assessments in participants who prematurely stop the study 42
9.3 Procedures at each visit 42
9.3.1 Screening and identification of patients (Up to 72 hours before randomisation) 42
9.3.2 Enrolment of participants (0-72 hours before randomisation)....42
9.3.3 Randomisation (day 1) 43
9.3.4 Assessment of baseline characteristics (day 1) 43
9.3.5 Treatment (day 1 – day 5)....43
9.3.6 Assessment of routine laboratory measurements (day 1-7)....44
9.3.7 Subproject *Molecular mechanisms of S. aureus bacteraemia* (day 1 until 48h after the patient's first negative blood culture) 44
9.3.8 Follow-up blood cultures (day 2, day 5 ±1)....44
9.3.9 Assessment of SIRS criteria (day 5 ±1)....44
9.3.10 Measurement of trough levels of linezolid in a subset of participants (day 4-5) 44
9.3.11 Assessment of adverse events of special interest (day 1-5, day 14 ±3, discharge day)....45
9.3.12 Assessment of serious adverse events and serious adverse reactions (from enrolment until discharge, and at day 90 ±7)....45
9.3.13 Assessment of new antibiotic resistance (discharge day) 45
9.3.14 Assessment of microbiological failure (day 28, discharge day, day 90 ±7) 46

9.3.15 Assessment of clinical failure (day 14, day 28, discharge day, day 90)....46
9.3.16 Assessment of length of hospital stay and length of ICU stay (discharge day)....46
9.3.17 Assessment of all-cause mortality (day 7, day 14, day 28, day 90 ±7)....46
9.3.18 Assessment of return to usual level of function (day 90 ±7)....46
9.3.19 Assessment of physical and mental health (quality of life, SF-36, day 90 ±7)....46
10. SAFETY 48
10.1 Drug studies....48
10.1.1 Definition and assessment of (serious) adverse events and other safety related events....48
10.1.2 Reporting of serious adverse events (SAE) and other safety related events....49
10.1.3 Follow up of (Serious) Adverse Events....50
10.2 Assessment, notification and reporting on the use of radiation sources....50
10.3 Exemption from the documentation requirements of AE....50
10.3.1 Exemption from the documentation requirements of AE due to pharmacological arguments....50
10.3.2 Exemption from the documentation requirements of AE due to clinical arguments....50
11. STATISTICAL METHODS 51
11.1 Hypothesis....51
11.2 Determination of Sample Size....51
11.3 Statistical criteria of termination of trial....51
11.4 Planned Analyses....51
11.4.1 Datasets to be analysed, analysis populations....51
11.4.2 Primary Analysis....52
11.4.3 Secondary Analyses....52
11.4.4 Interim analyses....53
11.4.5 Safety analysis....53
11.4.6 Deviation(s) from the original statistical plan....53
11.5 Handling of missing data and drop-outs....53
12. QUALITY ASSURANCE AND CONTROL.... 54
12.1 Data handling and record keeping / archiving....54
12.1.1 Electronic Case Report Forms (eCRF)....54
12.1.2 Specification of source documents....54
12.1.3 Record keeping / archiving....54
12.2 Data management....54
12.2.1 Data Management System....54
12.2.2 Data security, access, and back-up....54
12.2.3 Analysis and archiving....55
12.2.4 Electronic and central data validation....55
12.3 Monitoring....55
12.4 Audits and Inspections....55
12.5 Confidentiality, Data Protection....55
12.6 Storage of biological material and related health data....55
13. PUBLICATION AND DISSEMINATION POLICY.... 56
14. FUNDING AND SUPPORT 56
14.1 Funding....56
15. INSURANCE 56
16. TRIAL SUB-PROJECT(S) 57
16.1 Molecular mechanisms of *S. aureus* bacteraemia....57
17. REFERENCES 58
18. APPENDICES 64
18.1 Linezolid Stada® Summary of Product Characteristics....64
18.2 The Hunter Serotonin Toxicity Criteria: for diagnosing serotonin syndrome....64

STUDY SYNOPSIS

Sponsor	PD Dr. Richard Kühl<br>Department of Infectious Diseases<br>University Hospital Basel<br>Petersgraben 4<br>4031 Basel<br>Switzerland<br>Phone: +41 (0)61 328 66 61<br>Email: richardalexander.kuehl@usb.ch
Study Title:	Combination antibiotic treatment with linezolid for *Staphylococcus aureus* bacteraemia: a randomised controlled trial
Short Title / Study ID:	**Li**nezolid **p**lus **s**tandard of care (LIPS)
Protocol Version and Date:	Version 1.2 (dated 30/07/2025)
Trial registration:	ClinicalTrials.gov Identifier: NCT06958835<br>humanresearch-switzerland.ch: BASEC Nº2025-00655
Study category and Rationale	Risk Category B according to ClinO (Art 2)<br>• Placebo controlled antibiotic treatment with linezolid STADA® authorized in Germany
Clinical Phase:	Phase 3
Background and Rationale:	*Staphylococcus aureus (S. aureus)* is the leading bacterial cause of death in high-income countries. Bloodstream infection (bacteraemia) represents a major manifestation of *S. aureus* affecting 20-30 in 100,000 persons yearly. 90-day mortality rates of 20-30% have remained unchanged over the last decades. Furthermore, *S. aureus* spreads to secondary body sites in 6-44% of cases, causing long-term impairment in up to a third of survivors, with a relapse rate of 4-10%. While more men are affected by *S. aureus* bacteraemia (ratio of approximately 1.5:1), women have 18% higher odds of death (OR 1.18) compared to men. So far, more effective treatments have not been identified. Thus, finding better treatments for patients suffering from *S. aureus* bacteraemia is a high unmet medical need.<br>*S. aureus* expression of virulence factors facilitates the persistence and spread of the bacteria during infection and causes organ damage. We demonstrated the importance of *S. aureus* virulence factors in patho-mechanistic analyses directly from infected patient tissue. This led to our hypothesis that patient outcomes could be improved by adding an antibiotic that inhibits virulence factor expression. Several international guidelines recommend this strategy for toxin-mediated infections. However, due to a lack of evidence from randomised trials, this approach has not become standard clinical practice for bacteraemia with *S. aureus*. Linezolid, an established antibiotic for pneumonia or skin and soft tissue infections, strongly inhibited *S. aureus* virulence factor expression in preclinical studies.
Objectives:	**Primary objective:** To investigate whether early addition of 5-day treatment with linezolid to standard of care improves outcomes at 90 days in participants with *S. aureus* bacteraemia as measured by the desirability of outcome ranking (DOOR).<br>**Secondary objectives:**<br>Secondary objectives include exploring the impact of early addition of 5-day treatment with linezolid to standard of care on a number of secondary outcomes such as physical and mental health, length of hospital stay and safety (full list in section 5 STUDY OUTCOMES). In addition, we aim to explore if potential effects might differ between patient populations based on sex (male vs. female), ICU status at baseline (participants on ICU vs. participants not on ICU), and focus of infection (vascular catheter, skin and soft tissue, endocarditis, osteoarticular, pneumonia, other focus, or focus not identified).

Outcomes:	**Primary outcome**:

The **Desirability of Outcome Ranking (DOOR)** developed for this study comprises five ranks (Table 1) and is assessed at day 90. The primary outcome will be expressed as the win ratio, i.e., the ratio of the number of times that participants in the intervention group have a lower DOOR compared to those in the control group. In this study, we use the win ratio approach of Pocock et al. (2012), i.e., every participant in the linezolid group is compared with every participant in the control group. When comparing two participants, the winner will be determined by the first component of the DOOR in which the two participants differ, the only exceptions being ties when both participants die or if they do not die but have the same length of hospitalisation (see section 11 STATISTICAL METHODS for details).

Rank	Alive at 90 days	Return to usual level of function by day 90	None of the following complications:<br>• Microbiological or clinical failure leading to treatment change<br>• Serious adverse reaction<br>• Adverse event leading to study drug discontinuation	Hospital length of stay
**1**	Yes	Yes	Yes<br>(no complications occurred)	Ties will be resolved using the length of hospitalisation
**2**	Yes	Yes	No<br>(complication occurred)	
**3**	Yes	No	Yes<br>(no complications occurred)	
**4**	Yes	No	No<br>(complication occurred)	
**5**	No	Not applicable	Not applicable	Not applicable

Table 1: Desirability of Outcome Ranking (DOOR) outcome used for the LIPS trial

Secondary outcomes:

Day of randomisation is defined as day 1.

- All-cause mortality at 90 days
- Time to death up to day 90
- Proportion of participants back to their usual level of function at day 90
- Microbiological failure at 14-90 days leading to treatment change
- Early microbiological failure at 5-13 days leading to treatment change
- Clinical failure at 14-90 days leading to treatment change
- Early clinical failure at 5-13 days leading to treatment change
- Hospital length of stay after randomisation
- Time to being discharged alive (assessed by day 90)
- Number of days without being on the Intensive Care Unit (ICU) up to day 90
- Number of days alive and free of antibiotics up to day 90
- Mental health at day 90 (SF-36 questionnaire)
- Physical health at day 90 (SF-36 questionnaire)

	• Persistent bacteraemia: *S. aureus*-positive blood culture on day 5<br>• ≥ 2 systemic inflammatory response syndrome (SIRS) criteria on day 5<br>• Change in C-reactive protein on day 5<br>• Development of new antibiotic drug resistance in *Staphylococcus aureus*<br>**<u>Adverse events/safety:</u>**<br>• Serious adverse reactions until day 90<br>• Adverse events leading to study drug discontinuation<br>• Adverse events of special interest: clinical signs of serotonin toxicity, laboratory signs of myelosuppression, hyperlactatemia, acute kidney injury, *Clostridioides difficile*-associated diarrhoea within 90 days
Study design:	Multi-centric, 1:1 randomised, parallel group, placebo-controlled clinical trial with blinded participants, physicians, and outcome assessors conducted in the acute-care hospital setting.
Inclusion / Exclusion criteria:	Inclusion criteria:<br>• *S. aureus* grown from at least one blood culture<br>• Hospitalised at a participating centre<br>• ≥18 years old<br>• Written informed consent or fulfilling criteria for an emergency exception from informed consent requirements<br><br>Exclusion criteria:<br>• Administration of the initial drug treatment not feasible within 72 hours since the collection of the first positive blood culture with *S. aureus*<br>• Documented history of positive blood cultures for *S. aureus* occurring between 72 hours and 180 days prior to the eligibility assessment<br>• Necrotising fasciitis<br>• Currently receiving linezolid or clindamycin<br>• Use of any monoamine oxidase A or B inhibitor within the last two weeks<br>• Known hypersensitivity to linezolid or any other ingredients of the study drugs<br>• Current severe thrombocytopenia (i.e. <30 x 10^9/L)<br>• Application of study drug not possible (per mouth or per gastric tube)<br>• Currently breastfeeding<br>• Local treating team believes that death is imminent and inevitable<br>• Patient is receiving end of life care and antibiotic treatment is not considered appropriate<br>• Local treating team believes that participation in the study is not in the best interest of the patient<br>• Any indication that the patient is unwilling to participate in the study including an advance directive stating such unwillingness<br><br>General<br>Patients taking serotonergic drugs such as selective serotonin reuptake inhibitors (SSRI) or tricyclic antidepressants are not generally excluded. See detailed information on risk assessment and precautions in chapter 8.6 *Trial specific preventive measures*.<br>Pregnant women will not be excluded from study participation per se, as linezolid is not formally contraindicated during pregnancy. In the event of pregnancy, the treating physician and the patient (or the patient's next of kin) will assess whether the potential benefits for the patient outweigh the potential risks to the foetus.<br>Linezolid has been shown to pass into breast milk and accordingly, breastfeeding should be discontinued prior to and throughout administration of linezolid.

Measurements and procedures:	Infectious disease specialists, serving as the local principal investigators at all participating centres, are routinely involved in the care of patients with *S. aureus* bacteraemia. Hence, study teams at all participating sites will consider each patient with *S. aureus* bacteraemia for potential inclusion in the LIPS trial. The local investigators (or designated staff) will check inclusion and exclusion criteria, initiate the contact, and inform the patient (or a close relative or an independent physician if patient is incapable of judgement) about this trial. After the written informed consent is obtained, the participant will be randomised to the intervention group (i.e. linezolid 600 mg tablets, twice daily for 5 days in addition to standard of care antibiotic treatment) or the control group (i.e. placebo tablets in addition to standard of care antibiotic treatment). The LIPS trial will mainly use data generated as part of clinical routine during hospitalisation. In brief, baseline data (e.g., date of birth, gender, risk factors), microbiological results (e.g., blood culture results, resistance patterns), laboratory results (e.g., blood count, renal function), and data of concomitant treatments (standard antibiotic, source control procedures) will be collected from the hospital electronic medical records. Participants will be treated and monitored according to clinical routine. If a physician suspects an adverse event (e.g. serotonin toxicity, myelosuppression) they should order any assessments and tests necessary for the detection, as in routine clinical practice.<br>The outcomes “alive at day 90” and "return to usual level of function by day 90" will be assessed through a phone call on day 90 (given that the participant did not die during the hospitalisation). In addition, the participants will receive a questionnaire at day 90 to assess their quality of life (QoL, i.e. physical health and mental health).
Study Product / Intervention:	Participants in the intervention arm will receive linezolid 600 mg tablets (twice a day for 5 days) in addition to standard of care antibiotic treatment.
Control Intervention:	Participants in the control arm will receive oral placebo tablets (twice a day for 5 days) in addition to standard of care antibiotic treatment.
Number of Participants with Rationale:	606 participants (303 in control and 303 in the intervention group)<br>We will use the win ratio to assess if participants in the intervention group have a better overall clinical outcome as defined by the DOOR compared to participants in the control group taking the following domains into considerations: i) survival, ii) participant-reported level of functioning at 90 days compared to the level before the blood infection, iii) treatment change due to persistence or spread of the bacterium within day 14-90 or serious adverse events leading to study drug discontinuation, and iv) hospital length of stay in days.<br>The sample size was calculated using a simulation approach (including missing data in the assumptions), for which 1000 synthetic datasets for each combination of plausible values for the relevant parameters were generated and the intended primary analysis was applied. With a two-sided significance level of $\alpha=0.05$ and a desired power of 90% (1:1 allocation), an estimated 550 participants will be required to show a significant effect of the intervention. Because of possible post-randomisation withdrawal due to missing retrospective consent, the sample size was increased by 10% to minimise the risk of being underpowered.<br>See section 11 STATISTICAL METHODS for detailed assumptions about (i) occurrence of events and potential effect for each DOOR domain and (ii) about the frequency of missing data for each DOOR domain.
Study Duration:	Estimated duration for the main investigational plan (e.g. from start of screening of first participant to last participant processed and finishing the study):<br>38 months
Study Schedule:	First patient in: September 2025<br>Last patient out: October 2028
Local site-Investigators:	Please refer to the separate document “LIPS list of project leaders” for detailed information on local investigators.
Study Centres:	Approximately 12 institutions in Switzerland will be involved in this multi-centric trial. Please refer to the separate document “LIPS trial sites” for the complete list.
Statistical	Primary analysis:

Considerations:	Intervention and control groups will be compared based on the win-ratio approach as described by Pocock and colleagues: In brief, participants in the intervention group will be compared with participants in the control group. When comparing two participants, the winner will be determined by the first component of the DOOR in which the two participants differ. The following two scenarios will be considered a tie: (i) both participants died within 90 days, or (ii) both participants have the same outcomes for all DOOR elements, including hospital length of stay. The win ratio will be calculated by dividing the number of cases in which participants receiving linezolid win with the number of cases in which participants receiving placebo win. We will reject the null hypothesis that the win ratio is equal to 1 if the *p*-value calculated is less than 0.05.<br><br>More detailed information and intended approaches for the secondary analyses are listed in section 11 STATISTICAL METHODS. A detailed analysis plan will be written before closing the study database.
GCP Statement:	This study will be conducted in compliance with the protocol, the current version of the Declaration of Helsinki, ICH-GCP, as well as all national legal and regulatory requirements.

ABBREVIATIONS

ADL	Activities of Daily Living
AE	Adverse Event
ALT	Alanine Transaminase
Art.	Article
ASR	Annual Safety Report
BASEC	Business Administration System for Ethical Committees, (https://submissions.swissethics.ch/en/)
C. difficile	*Clostridioides difficile*
CA	Competent Authority (e.g. Swissmedic)
CD	Cluster of Differentiation
CEC	Competent Ethics Committee
CHUV	Centre hospitalier universitaire vaudois
CI	Confidence Interval
ClinO	Ordinance on Clinical Trials in Human Research *(in German: KlinV, in French: OClin, in Italian: OSRUm)*
CRF	Case Report Form
CRP	C-reactive protein
CTCAE	Common terminology criteria for adverse events
DKF	Department of Clinical Research, University Hospital Basel
DOOR	Desirability Of Outcome Ranking
DSMB	Data Safety Monitoring Board
DSUR	Development Safety Update Report
e.g.	For example
eCRF	Electronic Case Report Form
EDC	Electronic Data Capture
EOC	Ospedale Regionale di Lugano
FOPH	Federal Office of Public Health
GCP	Good Clinical Practice
gGT	Gamma-glutamyl Transferase
Hb	Haemoglobin
Ho	Null hypothesis
HOCH	Health Ostschweiz
HR	Heart Rate
HRA	Federal Act on Research involving Human Beings *(in German: HFG, in French: LRH, in Italian: LRUm)*
HUG	Hôpitaux Universitaires de Genève
i.e.	That is
IB	Investigator’s Brochure
ICF	Informed Consent Form
ICH	International Council on Harmonisation of Technical Requirements for Registration of Pharmaceuticals for Human Use
ICMJE	International Committee of Medical Journal Editors
ICU	Intensive Care Unit
ID	Identification
IICT	Investigator-Initiated Clinical Trial
IIT	Investigator-Initiated Trial

IMP	Investigational Medicinal Product
ITT	Intention To Treat
IV	Intravenous
KDIGO	Kidney Disease Improving Global Outcomes
KSA	Kantonsspital Aarau
Lc	Leukocytes
LIPS	**Li**nezolid **P**lus **S**tandard of Care
MALDI-TOF	Matrix-Assisted Laser Desorption/Ionization-Time of Flight
MF-DAC	Data Access Committee of the Faculty of Medicine at the University of Basel
MIC	Minimal Inhibitory Concentration
MRSA	Methicillin-Resistant S. aureus
MSSA	Methicillin-Susceptible S. aureus
OR	Odds Ratio
PCR	Polymerase Chain Reaction
PD	Privatdozent
PI	Principal Investigator
PPI	Patient and Public Involvement
PROM	Patient-Reported Outcome Measure
QoL	Quality of Life
RCT	Randomised Controlled Trial
RR	Respiratory Rate
S. aureus	*Staphylococcus aureus*
SAE	Serious Adverse Event
SAR	Serious Adverse Reaction
SCTO	Swiss Clinical Trial Organisation
SDV	Source Data Verification
SF-36	Short Form 36 Survey
SIRS	Systemic Inflammatory Response Syndrome
SNAP	*Staphylococcus aureus* Network Adaptive Platform
SNCPT	Swiss National Clinical Trials Portal
SNSF	Swiss National Science Foundation
SOP	Standard Operating Procedure
SMPC	Summary of Product Characteristics
SS	Serotonin Syndrome
SSRI	Selective Serotonin Reuptake Inhibitors
SUSAR	Suspected Unexpected Serious Adverse Reaction
Tc	Thrombocytes
TLS	Transport Layer Encryption
TMF	Trial Master File
US	United States of America
USB	University Hospital Basel
USZ	University Hospital Zurich
WHO ICTRP	World Health Organisation International Clinical Trials Registry Platform

SUMMARY OF THE REVISION HISTORY IN CASE OF AMENDMENTS

Version Nr, Version Date	Chapter	Description of change	Reason for the change
1.0, 27/03/2025		Initial version	
1.1, 27/06/2025		Minor changes throughout protocol before submission to Swissmedic	Incorporated feedback from competent Ethics committee
1.2, 30/07/2025	7.1, 8.6	Added “Currently breastfeeding” and “Use of any monoamine oxidase A or B inhibitor within the last two weeks” to exclusion criteria, changed wording of hypersensitivity criterion; minor changes in assessment description	Incorporated feedback from Swissmedic’s further information request; close alignment with linezolid SmPC

Docusign Envelope ID: 7FB1D507-0860-4709-95DA-14CC8893F28B

STUDY SCHEDULE

The study schedule can be found in chapter 9.1 *Study flow chart/ table of study procedures and assessments*.

1. STUDY ADMINISTRATIVE STRUCTURE

1.1 Monitoring institution

Clinical Trial Unit

Department of Clinical Research

University Hospital Basel

Spitalstrasse 8/12, 4031 Basel

Switzerland

Tel. +41 61 328 51 48

ClinicalTrials@usb.ch

1.2 Data Safety Monitoring Board

The trial will be reviewed by an independent and blinded Data Safety Monitoring Board (DSMB) who assesses safety during prespecified interim analyses. The DSMB will consist of at least three infectious disease experts unaware of the treatment assignments and not otherwise involved in the study. Members and frequency of meetings are described in the DSMB charter. For all serious adverse events (SAEs), the DSMB will assess if it is suspected to be related to the study drug (defining the SAE as an SAR).

1.3 Independent outcome assessment board

The outcomes “microbiological failure” and “clinical failure” (“complications” a and b, see 9.2.1 *Assessment of primary outcome*) will be reviewed by an independent and blinded outcome assessment board. The independent outcome assessment board will consist of at least three infectious disease experts unaware of the treatment assignments and not otherwise involved in the study. In case of disparity, results by the independent outcome assessment board will overrule site/investigator assessments. Members and frequency of meetings are described in the independent outcome assessment board charter.

1.4 Any other relevant Committee, Person, Organisation, Institution

1.4.1 Patient and Public Involvement (PPI)

Patient representatives will be involved at all stages of the trial. Detailed information can be found in 6.2 *Patient and Public Involvement*.

1.4.2 IMP Manufacturer

Spital-Pharmazie

Universitätsspital Basel

Spitalstrasse 26

4031 Basel

2. ETHICAL AND REGULATORY ASPECTS

Before the study is conducted, the protocol, the proposed patient information and consent form as well as other study-specific documents shall be submitted to a properly constituted Competent Ethics Committee (CEC) and Swissmedic, in agreement with local legal requirements, for formal approval. The CEC and Swissmedic approval will be communicated to the Sponsor-Investigator in writing before commencement of this study. The study will not start recruiting participants until ethical and regulatory approval has been granted. Any amendment to the protocol will, if legally required, also be approved by these institutions.

2.1 Study registration

This trial will be registered in the following registries before the recruitment of the first participant:

- http://humanresearch-switzerland.ch (BASEC Nº 2025-00655)
- ClinicalTrials.gov: https://clinicaltrials.gov/study/NCT06958835

2.2 Categorisation of study

Category B. A placebo-controlled study using an IMP authorized in Germany. Linezolid STADA® 600 mg tablets have been authorized in Germany, a country with equivalent medicinal product control in accordance with Article 13 Therapeutic Products Act, as a generic of Zyvoxid® 600 mg since 2014. Linezolid STADA® 600 mg is authorized for the treatment of pneumonia and complicated skin and soft tissue infections caused by Gram-positive bacteria. Recommended dose 600 mg twice a day. Recommended treatment duration is 10-14 days, but shorter treatment regimen may be sufficient for certain types of infections. *Staphylococcus aureus* is a Gram-positive bacterium and nearly always susceptible to linezolid. The effectiveness of linezolid in *S. aureus* bacteraemia as an oral continuation treatment after initial intravenous antibiotics is well-documented in observational studies. Drugs containing linezolid as their active ingredient have been approved in Switzerland for over 20 years.

2.3 Competent Ethics Committee (CEC)

The Sponsor-investigator will submit all Swiss centres for approval to the lead CEC (Ethikkommission Nordwest- und Zentralschweiz, EKNZ) in collaboration with the local Swiss investigators. No changes are made to the protocol without prior sponsor and CEC approval, except where necessary to eliminate apparent immediate hazards to study participants.

The sponsor-investigator will report the premature termination, interruption, or resumption of the study, including reasons thereof, to the CEC within 15 days. An interruption lasting more than two years is considered a premature termination. The sponsor-investigator will report the first visit of the first participant and the end of the study (defined as the last follow-up visit of the last study participant) to the CEC within 30 days. The final study report will be submitted within one year after study end or premature study termination. Amendments are reported according to chapter 2.10 *Protocol amendments*.

2.4 Swissmedic

The sponsor will obtain approval from Swissmedic before the start of the clinical trial. Reporting duties and timelines are the same as for CEC, except for non-substantial amendments that shall be reported as soon as possible. The application must be submitted to the CEC within two years of approval by the CA (ClinO Art. 23). An application for an extension beyond the two years is a substantial amendment; if this is not complied with, the approval to conduct the study lapses.

Amendments are reported according to chapter 2.10 *Protocol amendments*.

2.5 Ethical Conduct of the Study

The study will be carried out in accordance with the protocol and with principles enunciated in the current version of the Declaration of Helsinki, the guidelines of Good Clinical Practice (GCP) issued by ICH, and Swiss Law and Swiss regulatory authority's requirements. The CEC and regulatory authorities will receive annual safety reports (ASR) and interim reports and be informed about study stop/end in agreement with local requirements. In addition, the CEC will be informed annually about the general progress of the clinical trial.

2.6 Declaration of interest

There are no potential conflicts of interest to disclose.

2.7 Patient Information and Informed Consent

Patients will be informed about the study by their treating physician and consent will be obtained from each patient. Patients who are conscious and capable of making decisions will be approached to give their consent. The local investigator (or a designated physician) will explain the nature of the study, its purpose, the procedures involved, the expected duration, the potential risks and benefits and any discomfort it may entail. Each patient will be informed that the participation in the study is voluntary, that the patient may withdraw from the study at any time and that withdrawal of consent will not affect the patient's subsequent medical assistance and treatment. The patient will be informed that any questions can be asked, and that consulting family members, friends, their treating physicians, or other experts before deciding about study participation is possible. Enough time will be given before making the decision about study participation. However, it will be noted that the potential effect of the study medication (i.e. linezolid) might be greater if treatment can be started earlier. The patient will be informed that authorised individuals other than their treating physician may examine the patient's medical records.

Due to the nature of *S. aureus* bacteraemia, we expect approximately 10-20% of patients to be unresponsive and incapable of giving informed consent during the acute infection. These patients are particularly at risk of succumbing to the infection and should thus not be excluded from the study. In these emergency situations, consent will be obtained from the patient's next of kin. If no next of kin are present, an independent physician who safeguards the interests of the patient and is not involved in the conduct of the study can confirm participation ("confirmation by independent physician"). If there is any indication that the patient is unwilling to participate in the study including an advance directive stating such unwillingness, the patient will be excluded from participation. After patients regain capacity of judgement, they will be approached about giving retrospective consent.

We will provide the following specific information sheet and consent forms which will enable an informed decision about study participation:

1) Patient informed consent form
2) Next of kin informed consent form
3) Confirmation by Independent physician
4) Retrospective patient informed consent form

Formal consent, using the approved consent form, will be obtained before the patient is subjected to any investigation procedure. The signed consent form is retained as part of the trial records. Consent forms were developed in collaboration with patient representatives.

2.8 Participant privacy and confidentiality

The investigator affirms and upholds the principle of the participant's right to privacy and that they shall comply with applicable privacy laws. In particular, anonymity of the participants will be guaranteed when presenting the data at scientific meetings or publishing them in scientific journals.

Individual participant medical information (i.e. information that would identify an individual participant) obtained as a result of this study is considered confidential and disclosure to third parties is prohibited. Participant confidentiality will be further ensured by utilizing participant identification code numbers to correspond to treatment data in the computer files.

The investigator has appropriate knowledge and skills in the areas of data security and data protection or is able to ensure compliance by calling in appropriate expertise (Art. 6, ClinO).

For data verification purposes, authorized representatives of the sponsor, a competent authority (e.g., Swissmedic), or a CEC may require direct access to parts of the medical records relevant to the study, including participants' medical history.

2.9 Premature termination of the study

The Sponsor-Investigator in consultation with the DSMB may terminate the study prematurely before reaching the anticipated sample size of 606 participants according to certain circumstances, for example:

- ethical concerns
- insufficient participant recruitment
- when the safety of the participants is doubtful or at risk, respectively
- alterations in accepted clinical practice that make the continuation of a clinical trial unwise

If the study is terminated earlier than planned, the investigator notifies the CEC and Swissmedic according to the

provisions of ClinO Art. 38.

2.10 Protocol amendments

Suggestions for a protocol amendment can be made to the LIPS trial team by any group member and by all investigators. Should these impact trial specific procedures, additional on-site or web-based training will be provided to all investigators. Substantial amendments are only implemented after approval of the CEC and Swissmedic, respectively. Substantial amendments are changes that affect the safety, health, rights and obligations of participants, changes in the protocol that affect study objective(s) or central research topic, changes of trial site(s) or of trial leader and sponsor (ClinO, Art. 29).

Under emergency circumstances, deviations from the protocol to protect the rights, safety, and wellbeing of human participants may proceed without prior approval of the sponsor and the CEC/Swissmedic. Such deviations shall be documented and reported to the sponsor and the CEC/Swissmedic as soon as possible.

All non-substantial amendments are communicated to Swissmedic and to the CEC within the ASR.

3. BACKGROUND AND RATIONALE

3.1 Background and Rationale

Staphylococcus aureus (S. aureus) is the leading bacterial cause of death in high-income countries and estimated second leading bacterial cause of death globally after tuberculosis.[1] Bacteraemia (bacteria in the bloodstream) represents a common manifestation of *S. aureus* infection, affecting 20-30 in 100,000 persons annually.[2-4] These numbers have risen in recent years due to aging of the population and other risk factors such as the use of implantable medical devices.[2 4-6] Approximately 20-30% of patients with *S. aureus* bacteraemia die within 90 days.[7 8] This rate has not significantly changed for decades despite active standard antibiotic therapy[9 10] representing the highest mortality rate among all common bloodstream pathogens.[1] *S. aureus* bacteraemia is reported more often in men than in women.[11-13] Risk factors, as well as diagnostic and therapeutic management can differ between men and women.[13] Women may experience worse outcomes and higher mortality.[11 14-16]

In addition, *S. aureus* bacteraemia spreads to secondary body sites in 6-44% of cases,[17-20] produces prolonged impairment in up to one-third of survivors,[21] and is associated with a relapse rate of 4-10%.[22-24] Despite the magnitude of disease burden with an estimated 2 million people affected yearly worldwide, less than 3000 patients have been included in published randomised controlled trials (RCTs) testing treatments for *S. aureus* bacteraemia.[25] This leaves us with considerable uncertainty about an effective treatment.

Methicillin-susceptible *S. aureus* (MSSA) represents 95% of *S. aureus* antibiotic resistance profiles in Switzerland.[2] The standard management of MSSA bacteraemia consists of an intravenous treatment with an anti-staphylococcal beta-lactam antibiotic such as flucloxacillin or cefazolin for 2-6 weeks and rapid source control if possible.[26-29] Previous studies attempted to increase the antimicrobial efficacy of standard therapy by adding a second antibiotic with evidence of additive or synergistic killing capacity *in vitro*. As identified by a recent systematic review,[30] gentamicin,[31-33] rifampicin,[34 35] levofloxacin,[36] daptomycin,[37] or fosfomycin[38] have been added to standard antibiotics in RCT of bacteraemia with methicillin-susceptible *S. aureus* (Table 2). Unfortunately, none of these RCTs succeeded in demonstrating a significant improvement in clinical outcomes. Several factors may have contributed to the shortcomings of these clinical trials. One possible factor is that the interventions may not have effectively addressed the underlying pathophysiologic factors responsible for the poor outcomes in patients with *S. aureus* bacteraemia. Limitations of the RCTs also include that the sample sizes were generally small, making it difficult to detect small, but relevant differences in mortality. In addition, these RCTs did not prioritise the assessment of other important patient-related outcomes, such as post-infection functional status.

Study (year)	RCT design sample size	Population	intervention	Control	PROM	Primary outcomes
Abrams (1979) [25]	open-label single-centre N=35	*S. aureus* endocarditis in intravenous drug users	Standard + **gentamicin**	Standard (Beta-lactam)	no	time to defervescence: I: 6.3 days vs. C: 6.6 days mortality/relapse of infection I: 0%. vs. C: 0% **No difference** in primary outcome
Korzeniowski (1982) [26]	open-label multicentre n=78	*S. aureus* endocarditis in drug-addicts and non-addicts	Standard + **gentamicin**	Standard (nafcillin)	no	cure: I: 60% vs. 65% **No difference** in primary outcome
Van der Auwera (1985) [28]	double-blind placebo-controlled multicentre n=29 (bacteraemia)	*S. aureus* infections (subgroup with bacteraemia)	Standard + **rifampicin**	Standard (oxacillin / vancomycin) + **placebo**	no	clinical cure: I: 69%, C: 63% **No difference** in primary outcome
Ribera (1996) [27]	open-label single-centre n=90	*S. aureus* right-sided endocarditis	Standard + **gentamicin**	Standard (cloxacillin)	no	cure (no persistence/relapse/death): I: 86%, C: 89% **No difference** in primary outcome
Ruotsalainen (2006) [30]	open-label multicentre n=381	*S. aureus* bacteraemia	Standard + **levofloxacin**	Standard (semisynthetic penicillin)	no	28-d mortality: I:14%, C: 14% 90-d mortality: I:18%, C: 21% **No difference** in primary outcome
Thwaites (2018) [29]	double-blind placebo-controlled multicentre n=758	*S. aureus* bacteraemia (MSSA/MRSA)	Standard + **rifampicin**	Standard (flucloxacillin / vancomycin) + **placebo**	no	failure: (persistence/recurrence/death): I: 17%, C: 18% **No difference** in primary outcome
Cheng (2020) [31]	double-blind placebo-controlled multicentre n=104	*S. aureus* bacteraemia (MSSA)	Standard + **daptomycin**	Standard (cefazolin/ flucloxacillin) + **placebo**	no	90-d mortality: I: 18.9%, C: 17.7% Duration of bacteraemia: I: 3.09 d, C: 3.0 d **No difference** in primary outcome
Grillo (2023) [32]	open-label multicentre n=215	*S. aureus* bacteraemia (MSSA)	Standard + **fosfomycin**	Standard (cloxacillin)	no	7-d treatment success (alive/better qSOFA score/afebrile/negative blood culture): I: 79.8%, C: 74.5% **No difference** in primary outcome

Table 2: Overview of published randomised controlled trials (RCT) testing for superiority in methicillin-susceptible S. aureus bacteraemia (identified by the systematic review by Grillo et al. [30], and Grillo et al [38])
Abbreviations: PROM: patient-related outcome measure; I: intervention group, C: control group, MSSA: methicillin-susceptible S. aureus; MRSA: methicillin-resistant S. aureus

Thus, there is an urgent clinical need to evaluate new therapeutic strategies through high-quality RCTs with the aim of improving patient-relevant outcomes in *S. aureus* bacteraemia.

S. aureus is known for its ability to survive and rapidly adapt to new environments in the human body.[39 40] It employs a wide range of virulence factors, including toxins to attack human (immune) cells[41] and proteases to invade human tissue[42], often eliciting a strong inflammatory response at the site of infection. It can be inferred that most of the damage to the body results from direct and indirect effects of these virulence factors. The impact of indirect damage and physical strain from the infection is evident, as 40-60% of deaths in people with *S. aureus* bacteraemia happen after the initial infection and cause of death cannot be clearly linked to the infection.[17 43] This finding is consistent with prior research involving patients with sepsis.[44] Furthermore, akin to sepsis as a whole, it is imperative to consider not only mortality but also long-term functional and cognitive impairments resulting from the direct and indirect consequences of the infection. This aspect represents a thus far underexplored area of research.[45 46]
The significance of virulence factor expression has been observed in data obtained directly from patient samples as part of our research project within the National Centre of Competence in Research (publication in preparation). Determining the role of virulence factors becomes even more important considering that standard treatment with a beta-lactam-type antibiotic can augment bacterial toxin production as long as *S. aureus* is not killed.[47] Thus, it has been hypothesised that adding an antibiotic agent inhibiting virulence factor expression could reduce direct and indirect organ damage thereby improving patient outcomes.[48] Linezolid strongly reduced the expression of *S. aureus* virulence factors in preclinical studies,[47] making it an ideal candidate to use clinically. Expert opinion and some guidelines advocate this strategy for severe infections or for *S. aureus* strains with known toxin expression.[49-52] However, evidence from RCTs for this approach is still lacking: our systematic literature search for published or planned RCTs (in PubMed, Embase, and the Cochrane Central Register of Controlled Trials [indexing planned RCTs from clinicaltrials.gov and WHO ICTRP]) that was developed by an information specialist from the University Medical Library yielded 943 hits (search conducted in June 2023). None of those reported RCT results or an ongoing RCT involving an antibiotic combination treatment with linezolid for *S. aureus* bacteraemia.
Currently, an international platform trial called SNAP ("*Staphylococcus aureus* Network Adaptive Platform")[25] compares various treatment options for *S. aureus* bacteraemia including one study domain that assesses open-label adjunctive treatment with clindamycin — a different antibiotic recognised for its toxin-inhibiting properties. Our proposed study, in contrast, will employ linezolid as the adjunctive intervention, thus providing crucial complementary evidence to the findings of the SNAP trial's adjunctive clindamycin.

In summary, effective treatment of *S. aureus* bacteraemia is a high unmet medical need. Our hypothesis suggests that *S. aureus* virulence factors are responsible for these negative outcomes and that early intervention to suppress these factors may lead to improved clinical outcomes. We also consider that the chosen outcomes to date have not covered the whole spectrum of effects associated with the disease. Our goal is to evaluate addition of linezolid to standard of care in a robust RCT with patient-relevant endpoints to achieve better outcomes for patients with *S. aureus* bacteraemia.

3.2 Investigational Medicinal Product (treatment) and Indication

Linezolid STADA® 600 mg tablets and oval placebo tablets (Fagron) will be imported from Germany by Spital-Pharmazie Basel, Spitalstrasse 26, 4031 Basel. Linezolid STADA® 600 mg is authorized for the treatment of pneumonia and complicated skin and soft tissue infections caused by Gram-positive bacteria. Recommended treatment duration is 10-14 days, but shorter treatment regimen may be sufficient for certain types of infections[53].

Packaging and labelling will be performed by Spital-Pharmazie Basel to ensure blinding. A sample of the label will be enclosed to the Swissmedic application.

Detailed information on Linezolid STADA® 600 mg can be found in the provided Summary of Product Characteristics (SMPC).

3.3 Preclinical Evidence

Linezolid inhibits bacterial protein synthesis at an early stage by binding to the 50S subunit of the bacterial ribosome. Animal data revealed enhanced activity of linezolid combined with vancomycin or imipenem in experimental endocarditis models,[54 55] as well as in combination with fosfomycin in a murine peritonitis model.[56] Additionally, linezolid effectively decreased *in vivo* toxin production[57] and was much more effective than vancomycin in protecting animals from dying in pneumonia models.[57-59]

A summary of relevant preclinical data is included in the SMPC.

3.4 Clinical Evidence to Date

Linezolid is an established antibiotic treatment option for skin- and soft tissue infections as well as for pneumonia, especially in case of MRSA due to the low rate of linezolid resistance.[60] Detailed information on pivotal studies of linezolid can be found in the SMPC. For nosocomial MRSA pneumonia, linezolid has been shown to be superior to the commonly administered intravenous vancomycin.[59] For MRSA bacteraemia, linezolid is a safe and effective alternative to standard-of-care glycopeptide (vancomycin/ teicoplanin).[61-63] A trial directly comparing standard-of care beta-lactam therapy with linezolid in MSSA bacteraemia is lacking, since beta-lactam therapy is regarded as most effective for MSSA. Limited clinical data exist regarding combination therapy with linezolid in *S. aureus* bacteraemia. Two observational cohort studies (involving a total of 54 linezolid treated participants) reported favourable outcomes (microbiological response, success rate, mortality) with salvage combination therapy using linezolid and a carbapenem.[64 65] Successful treatment was also observed in a case of MRSA bacteraemia and meningitis with linezolid, daptomycin, and rifampicin.[66] Linezolid may be most effective when started as early as possible according to the open label RCT by Wilkox et al.[67] Even though the study was underpowered, it supports the hypothesis that empirically initiated linezolid might result in better survival. Similarly, a prospective observational single-centre study found significantly improved survival in participants with MRSA bacteraemia empirically receiving linezolid compared to a glycopeptide (adjusted odds ratio for survival 7.7; 95%CI 1.1–53).[68]

The potential clinical impact of toxin inhibition by linezolid is demonstrated in a case report on the successful treatment of a staphylococcal toxic shock syndrome.[69] In addition, a review of 93 cases of *S. aureus* necrotizing pneumonia revealed reduced mortality rates when antibiotic therapy incorporated an anti-toxin agent, such as clindamycin or linezolid.[70] Our systematic search showed that no RCT has assessed the effect of adding linezolid to the standard antibiotic therapy in patients having *S. aureus* bacteraemia.

In summary, linezolid is a valuable antibiotic for various *S. aureus* infections, especially for MRSA cases in which beta-lactams are ineffective. Early use of linezolid, as well as late salvage treatment using linezolid in combination with a beta-lactam for persistent bacteraemia and toxin-mediated infections has been documented. However, no study has evaluated early combined treatment of linezolid with standard therapy for *S. aureus* bacteraemia to mitigate potential toxin-related complications and improve patient relevant outcomes.

3.5 Rationale for the dosage, route, regimen

Linezolid will be applied in addition to the standard antibiotic treatment prescribed by the treating physician.

Linezolid is approved according to the Swissmedic-approved prescribing information of a reference product with marketing authorisation as follows: 600 mg IV or oral twice daily for 10-14 days in case of nosocomial pneumonia and complicated skin and soft tissue infections, and 600 mg IV or oral twice daily for 14-28 days in case of vancomycin-resistant enterococcus infections including enterococcal bacteraemia. Treatment duration is variable and dependent on pathogen, site and severity of infection, as well as clinical response of the patient. The maximum treatment duration is 28 days. Due to linezolid's excellent oral bioavailability of nearly 100%,[71] we selected the oral dosing route for our study. This is in line with patient preferences. It has the additional benefit that participants do not need an intravenous catheter for the study which would increase the risk of catheter-related infections. Moreover, participants are not burdened by the additional administration of fluids, which can have negative cardiovascular effects, especially in patients with heart failure.

We expect the highest therapeutic benefit of linezolid within the first three days following detection of infection. Beyond this early period, we hypothesize that damage caused by the expression of virulence factor has already occurred, leading to decreased potential efficacy and benefit of linezolid. Thus, in line with our hypothesis we have reduced the treatment duration to 5 days which must start within 72 hours after the collection of the first positive blood culture.

3.6 Explanation for choice of comparator

Placebo will be applied in addition to the standard antibiotic treatment prescribed by the treating physician. Some primary outcome measures might be influenced by expectations of physicians and participants. The blinded study design ensures blinding of caregivers (including nurses, treating physicians), the participant, the investigators, and outcome assessors, and mitigates the bias associated with conduct of an open trial.

3.7 Risks / Benefits

Risks

The most commonly reported adverse event of linezolid is gastrointestinal intolerance, occurring in approximately 30-40% of patients when linezolid is administered for a longer duration.[72 73] Within the first 5 days of treatment, gastrointestinal intolerance manifests in approximately 8% leading to early discontinuation in 3%.[73] Myelosuppression, which primarily presents as thrombocytopenia, is another common adverse effect of linezolid that affects up to a third of patients[74-77] and occurs more frequently during treatment durations of ≥10 days.[74 78] Moreover, linezolid is a weak, reversible, and unselective monoamine oxidase inhibitor.[79] Consequently, the combination of linezolid with other serotonergic drugs, such as selective serotonin reuptake inhibitors (SSRIs) or tricyclic antidepressants, can potentially lead to an increase in serotonin activity. The clinical effects of serotonin excess can range from mild serotonin-related side effects to severe serotonin toxicity.[80 81] In the largest meta-analysis involving 2208 patients receiving linezolid and 5058 patients not receiving linezolid, the observed incidence of serotonin toxicity was 0.4% among patients taking linezolid in combination with a serotonergic agent (compared to 0.15% in those taking a serotonergic agent alone).[82] Similarly, in a large cohort of 1135 patients, fewer than 0.5% experienced serotonin toxicity.[83] Further, hyperlactatemia is a rare but potentially severe adverse effect of linezolid documented in various case reports.[72 84 85] The incidence is not well reported, but may be at 1-3% according to retrospective studies.[73 86] Finally, it should be noted that linezolid can potentially cause peripheral and/or optic neuropathy if treatment is administered for more than 28 days.[85 87] Given the short treatment duration of 5 days, we expect a low incidence of these adverse events in our study. Nevertheless, we will closely monitor the administration of the drug and, if necessary, terminate treatment in response to adverse events (see 8.6 *Trial specific preventive measures*).

Benefits

S. aureus bacteraemia represents a high burden not only on each affected individual person, but also on the community and on the healthcare system in general. [88 89] Even a modest improvement in outcomes resulting from our intervention would yield significant overall benefits, alleviating both the direct and indirect consequences of this still very severe infection. Should our novel approach against *S. aureus* bacteraemia prove effective, it holds the potential to revolutionise the therapeutic management of patients with this condition, not only in Switzerland, but on a global scale. Furthermore, it could serve as a catalyst for both basic and clinical research to explore even more efficacious treatments with an anti-virulence approach.

3.8 Justification of choice of study population

A systematic review from 2022 reported that the median age of patients with *S. aureus* bacteraemia ranged from 62 to 72 years[4] . A retrospective observational study looking at cases of *S. aureus* bacteraemia in Switzerland between 2008 and 2021 found that 92% of all *S. aureus* patients were adults[2]. Overall, mortality seems to be lower in children (2-15%)[90-93] suggesting that the disease may manifest differently in children compared to adults. Due to this difference, we will not include children.

In all participating hospitals, infectious disease specialists serving as the local principal investigators, are routinely involved in the care of patients with *S. aureus* bacteraemia. We will approach all patients with *S. aureus* bacteraemia, regardless of sex or gender, who meet the inclusion criteria and do not meet any exclusion criteria about participating in the LIPS trial. Approximately 10-20% of eligible patients will not be able to provide consent themselves due to their compromised health status caused by the *S. aureus* bacteraemia. These patients stand to benefit the most from improved treatment and should not be categorically excluded from trial participation. Therefore, we will include these patients whenever feasible by obtaining consent from a close relative or confirmation by an independent physician not involved in the study. Once the participant's health improves sufficiently to make an informed decision, delayed consent will be sought directly from the participant. We will follow Swissethics guidelines for obtaining consent in emergency situations.[94]

Patients who are pregnant will not be excluded from study participation per se, as linezolid is not formally contraindicated during pregnancy. Limited data exist on the treatment of pregnant women with linezolid, mainly from clinical trials in treating multidrug-resistant tuberculosis. In 31 recorded pregnancies under linezolid exposure in 7 clinical trials, none reported foetal toxicity.[95] In the event of pregnancy, the treating physician and the patient (or the patient's next of kin) will assess whether the potential benefits outweigh the potential risks to the foetus. As linezolid has been shown to pass into breast milk,[96 97] breastfeeding should be discontinued prior to and throughout administration of linezolid.

Linezolid is a weak, reversible, and unselective monoamine oxidase inhibitor.[79] Patients whose background

medication includes other serotonergic drugs such as selective serotonin reuptake inhibitors (SSRIs) or tricyclic antidepressants, can potentially experience an increase in serotonin activity. The clinical effects of serotonin excess range from mild serotonin-related side effects to severe serotonin toxicity.[80 81] In the largest meta-analysis involving 2208 patients receiving linezolid and 5058 patients not receiving linezolid, the observed incidence of serotonin toxicity was 0.4% among patients taking linezolid in combination with a serotonergic agent (compared to 0.15% in those taking a serotonergic agent alone).[82] Similarly, in a large cohort of 1135 patients, fewer than 0.5% experienced serotonin toxicity.[83] Assuming 20% of study participants are on serotonergic drugs, we would expect an additional 0-1 participants with serotonin toxicity in the linezolid arm compared to the placebo arm. In view of the low incidence of clinically relevant serotonin toxicity combined with the study design ensuring adequate monitoring of participants, patients on serotonergic agents are not excluded from study participation. A detailed list of such medications can be found in chapter 8.6 *Trial specific preventive measures*.

3.9 Summary of Background and Data on Linezolid

S. aureus bacteraemia remains a life-threatening condition with high morbidity and a mortality of up to 30% despite antibiotic therapy. Emerging evidence suggests that bacterial virulence factors significantly contribute to organ damage and poor patient outcomes. Linezolid, a well-established antibiotic with anti-virulence factor properties, offers a promising adjunctive treatment option. Preclinical studies demonstrate that linezolid effectively inhibits *S. aureus* virulence factor expression and improves survival in animal models. Clinical data support its safety and efficacy in various *S. aureus* infections, including pneumonia and bacteraemia.

Since its approval more than 20 years ago, the risk profile of linezolid has been well-documented. The approved treatment duration varies from 10-28 days depending on the cause and location of the infection. Notable adverse events of linezolid are myelosuppression, serotonin syndrome, kidney injury, *C. difficile*-associated diarrhoea, lactic acidosis, and neuropathy. Myelosuppression is typically associated with prolonged use (≥10 days) and is reversible upon linezolid discontinuation. Serotonin syndrome is very rare with monotherapy and remains uncommon even with concurrent serotonergic medications. Renal adverse effects including increased creatinine and rare cases of renal failure have been reported with linezolid use. Like many antibiotics, linezolid has been associated with *C. difficile*-associated diarrhoea, which can range from mild diarrhoea to fatal colitis. Lactic acidosis is rare but serious and is linked to extended treatment durations. Neuropathy has been described in patients with treatment durations exceeding the maximum recommendation of 28 days and is thus very unlikely to occur in this trial.

Since linezolid will be administered for only 5 days in the LIPS trial, a favourable safety profile is expected. In addition, hospitalization of study participants facilitates a close monitoring for any adverse events.

Given the significant unmet clinical need and the favourable safety profile of short-term linezolid therapy, the anticipated benefits — including reduction of virulence factor-mediated complications and improvement in patient-relevant outcomes — clearly outweigh the manageable risks. The trial will provide critical evidence on whether early adjunctive treatment with linezolid improves outcomes in *S. aureus* bacteraemia. If linezolid proves to be effective in treating *S. aureus* bacteraemia, it holds the potential to improve the therapeutic management of millions of patients with this condition worldwide.

4. STUDY OBJECTIVES

4.1 Overall Objective

The purpose of the LIPS trial is to evaluate whether the addition of linezolid to standard therapy in participants with *S. aureus* bacteraemia leads to an overall improvement in patient-related outcomes such as mortality, health-related quality of life, complications, and duration of hospitalisation.

4.2 Primary Objective

The primary objective is to investigate whether early addition of 5-day treatment with linezolid to standard of care improves outcomes at 90 days in participants with S. aureus bacteraemia as measured by the desirability of outcome ranking (DOOR).[98 99]

4.3 Secondary Objectives

Secondary objectives include exploring the impact of early addition of 5-day treatment with linezolid to standard of care on a number of secondary outcomes such as physical and mental health, length of hospital stay (full list in section 5 STUDY OUTCOMES). In addition, we aim to explore if potential effects might differentiate between patient population based on sex (male vs. female), ICU status at baseline (participants on ICU vs. participants not on ICU), and focus of infection (vascular catheter, skin and soft tissue, endocarditis, osteoarticular, pneumonia, other focus, or focus not identified).

4.4 Safety Objectives

The LIPS study aims to assess the safety of early addition of 5-day treatment with linezolid to standard of care treatment for *S. aureus* bacteraemia in a real-world setting. Details on adverse events of special interests can be found in chapters 5.4 *Safety Outcomes* and 9.2.4 *Assessment of safety outcomes*.

5. STUDY OUTCOMES

5.1 Primary Outcome

The primary outcome utilises the Desirability of Outcome Ranking (DOOR), which has been specifically introduced as a patient-centred outcome for *S. aureus* bacteraemia.[100 101] This hierarchical composite outcome ranking has the advantage that it can incorporate multiple outcomes (both beneficial and harmful) to measure if patients experience better overall outcomes after receiving a specific intervention compared with an appropriate comparator ("*Good Studies Evaluate the Disease While Great Studies Evaluate the Patient*"[100]). In contrast to composite outcomes, the DOOR outcome does not have the limitation that events of different importance are weighted equally.[102] We modified the proposed ranking based on the feedback of our patient representatives and the ongoing SNAP trial.[103] The most important outcome for patient representatives was surviving the infection. The second most important aspect was that they "are the same as before the disease", meaning that they have the same level of function as before the *S. aureus* infection. Patient representatives with a history of *S. aureus* bacteraemia informed us that results of their bacterial blood culture were of highest relevance to them during their hospital stay due to high uncertainty of the progression of the disease. This was particularly important because diagnostic evidence of persistent, relapsing or new *S. aureus* infection results in new diagnostic steps, interventions and/or new exposure to antibiotics with their accompanying toxicities. Therefore, we have implemented the third domain *complications*, including microbiological and clinical failure leading to treatment change, and serious adverse reactions or adverse events leading to study drug discontinuation. Finally, hospital length of stay was included as the fourth DOOR component, since patient representatives mentioned it as an important outcome. We will use outcome definitions consistent with the SNAP trial to facilitate data comparisons.

DOOR comprises five ranks (Table 3: Desirability of Outcome Ranking (DOOR) outcome used for the LIPS trial). This outcome is assessed at day 90. The overall distribution of rankings can then be compared between the control and intervention arms, with the final treatment effect estimate being the win ratio, i.e., the probability that a participant randomly assigned to the intervention arm has a superior DOOR compared to a participant randomly assigned to the control arm[99] (see section 11 STATISTICAL METHODS).

Rank	Alive at 90 days	Return to usual level of function by day 90	None of the following complications:<br>• Microbiological or clinical failure leading to treatment change<br>• Serious adverse reaction<br>• Adverse event leading to study drug discontinuation	Hospital length of stay
1	Yes	Yes	Yes (no complications occurred)	Ties will be resolved using the length of hospitalisation
2	Yes	Yes	No (complication occurred)	
3	Yes	No	Yes (no complications occurred)	
4	Yes	No	No (complication occurred)	
5	No	Not applicable	Not applicable	Not applicable

Table 3: Desirability of Outcome Ranking (DOOR) outcome used for the LIPS trial

Detailed definitions of DOOR components:

Alive at 90 days: defined as alive at 90 days after randomisation

Return to usual level of function by day 90: defined as return to baseline value (or better score) according to the modified functional bloodstream infection score[104]. Baseline is defined as the best score within the 4 weeks before randomisation. The scores used will be the following:

6: Out of hospital, able to complete daily activities without assistance (no limitations)

5: Out of hospital, able to complete daily activities without assistance but with some limitations (e.g. slow, pain)

4: Out of hospital, unable to complete daily activities without assistance

3: Out of hospital; significant disability; requires a high level of care and assistance daily (this includes residential

aged care and nursing homes)

2: Hospitalised (or equivalent)

1: On palliative care in terminal phases of life (in hospital or at home)

Complications:

- Microbiological or clinical failure leading to treatment change: This includes any type of adaptation of the treatment such as new intervention or surgery, or re-start, prolongation or change of antibiotic treatment
    - Microbiological failure: Any positive sterile site culture with *S. aureus* between 14 and 90 days after randomisation. A sterile site means any site of the body where microorganisms are usually absent, i.e. below the outer and inner colonised surfaces of the skin and mucous membranes. Positive sterile sites cultures include deep visceral and musculoskeletal abscesses obtained in a sterile manner.
    - Clinical failure: Newly identified focus of *S. aureus* between 14 and 90 days after randomisation as determined by the site investigator (or delegated physician). This can incorporate clinical, radiological, microbiological and pathological findings.
- Serious adverse reaction (SAR, see definition below)
- Any adverse event (irrespective of grade) leading to study drug discontinuation

Hospital length of stay: This corresponds to the duration of the index acute hospital stay from randomisation until the day of hospital discharge. Transfers to another acute care hospital for continuation of acute treatment will be included in the assessment of hospital length of stay. Days after transfer to rehabilitation centres or switch to outpatient parenteral ambulatory treatment will not be included in the acute hospital stay.

5.2 Secondary Outcomes

The secondary outcomes include each part of the DOOR outcome separately as well as additional patient-relevant outcomes and specific outcomes of interest to assess the influence on infection dynamic and control. The secondary outcomes have been aligned to the SNAP trial to allow comparison of the data at a later time point.

Separate parts of the DOOR outcome:

- All-cause mortality at 90 days
- Return to usual level of function at day 90
- Microbiological treatment failure 14-90 days after randomisation leading to treatment change
- Clinical treatment failure 14 and 90 days after randomisation leading to treatment change
- Length of hospital stay of acute index hospitalisation

  Adverse events leading to study drug discontinuation

Clinical outcomes:

- Time to death up to day 90
- Time to being discharged alive
- Number of days without being on the ICU up to day 90

Infection dynamics and control:

- Persistent bacteraemia: positive blood culture on day 5 (±1 day) after randomisation
- Two or more systemic inflammatory response syndrome (SIRS) criteria on day 5 after randomisation
- Change in C-reactive protein (CRP) from day 1 until day 5 (±1 day) after randomisation
- Early microbiological treatment failure 5-13 days after randomisation leading to treatment change
- Early clinical treatment failure 5-13 days after randomisation leading to treatment change

- Development of any new antibiotic drug resistance in *S. aureus* until day 90
- Number of days alive and free of antibiotics in the 90 days

Patient-reported outcomes:

- Physical health at day 90 as assessed by the short form 36 (SF-36) questionnaire.[105-107]
- Mental health at day 90 as assessed by the short form 36 (SF-36) questionnaire.[105-107]

5.3 Other Outcomes of Interest

Additional laboratory outcomes for a subset of participants only in participating centres:

- Linezolid plasma concentrations at day 4 or 5
- Minimal inhibitory concentration (MIC) of linezolid for the respective *S. aureus* strain

5.4 Safety Outcomes

Safety outcomes encompass any serious adverse events (SAE) and serious adverse reactions (SAR) until day 90 as well as adverse events of special interest. Definitions can be found in chapter 10.1.1 *Definition and assessment of (serious) adverse events and other safety related events*.

Adverse events of special interest

(details on how they are assessed is provided in chapter 9.2.4.1 *Adverse events*:

- Clinical signs of serotonin toxicity until day 7
- Laboratory signs of myelosuppression until day 7
- Hyperlactatemia until day 7
- Acute kidney injury
- *C. difficile*-associated diarrhoea within 90 days

Further information on safety outcomes (mortality, SAR, AEs leading to discontinuation of trial intervention) can be found in chapters 5.1 *Primary Outcome* and 5.2 *Secondary Outcomes*.

6. STUDY DESIGN

6.1 General study design and justification of design

LIPS is a pragmatic, multicentre, 1:1 randomised, parallel-group, placebo-controlled, superiority trial with blinded participants, physicians, and outcome assessors in the acute-care hospital setting (see Figure 1).

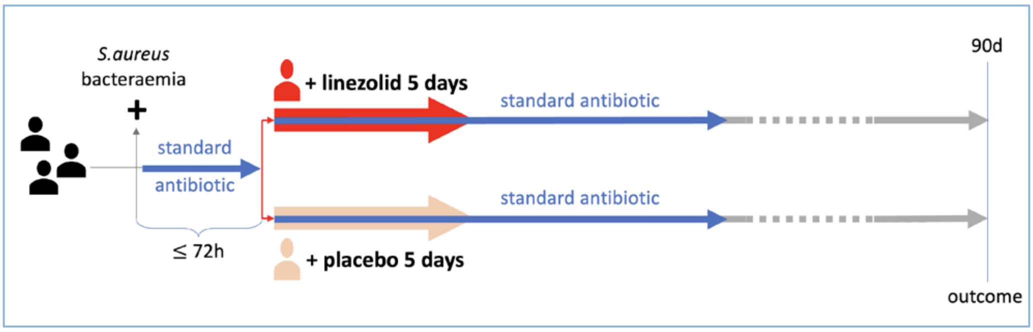

Figure 1: Schematic overview of the trial

The LIPS trial was designed with the involvement of four patient representatives to make sure that the assessed outcomes are patient relevant and that the burden due to the trial remains minimal for the participants. We have chosen a pragmatic trial mainly following routine care (i.e. the majority or outcomes are collected during routine care and the additional follow-up consists only of one phone call at day 90 and a quality-of-life questionnaire at day 90). However, as the outcomes can be influenced by expectations from participants (i.e. level of function) as well as the treating physicians (i.e. hospital length of stay) we have chosen a blinded study design to eliminate this source of bias.

6.2 Patient and Public Involvement

The following Patient and Public Involvement (PPI) has been and will be conducted to ensure that the trial is highly relevant to patients. We have been collaborating with four PPI representatives (two patient representatives who had complicated *S. aureus* bacteraemias as well as two patient experts) since the preparation of the funding application. These PPI representatives have agreed to lend their expertise for the duration of the trial up to the dissemination of the trial results. Ahead of every meeting, the PPI representatives are briefed on the current stage of the trial and the purpose of the meeting in layman's terms. Before the finalisation of the first protocol and patient informed consent draft, we conducted 3 focus group discussions with the four PPI representatives (2-4 hours/meeting).

6.2.1 PPI activities before receiving approval from authorities

PPI contribution	Outcome
- Advised on the patient relevance of the proposed outcomes.<br>- Informed the researchers about important aspects not covered by the proposed outcomes.<br>- Discussed what the primary outcome should be and help with the exact definition.	- Trial outcomes that are relevant to patients were chosen and ranked in order of importance (DOOR outcome).<br>- PPI representatives advised to include a patient-reported outcome measure on quality of life at day 90.
- Reviewed the eligibility criteria with a focus on inclusivity and making the trial results generalizable.	- Broad inclusion criteria were chosen as recommended by the PPI representatives.<br>- Exclusion criteria focus on safety aspects.
- Provided feedback on how understandable the provided lay summary was.	- Parts of lay summary were flagged as needing further explanation or simplifications and edited accordingly.
- Advised on the chosen time points and if the study visits are useful and patient friendly.	- Outcomes may also be collected by asking the participant's relatives or general practitioner.<br>- Burden for participants with the proposed trial

- Assessed burden for patients and identified ways to decrease burden, dropout rates and incomplete data selection.	design was deemed very low.
- Defined the further study phases during which PPI is critical and what form such involvement should take (guided by SCTO template).	- PPI activities during and after trial conduct were planned (see chapter 6.2.2 *PPI activities after receiving approval from authorities*).
- Provided feedback on draft patient information and informed consent form (ICF) in German.<br>- Some PPI representatives will also assess patient information in other languages	- ICF is concise and comprehensible, giving enough information without frightening patients regarding their diagnosis.<br>- Patient-relevant information on background of the underlying disease, study procedures, risks and adverse events in layman's terms are included.

6.2.2 PPI activities after receiving approval from authorities

Planned PPI contribution	Objective
- Advise on how to approach patients and relatives to inform them about study participation.	- Incorporating PPI feedback into trainings of study sites on how to approach patients and relatives in a sensitive manner.
- Participation in DSMB during prespecified interim analyses	- Including the patient perspective when serious adverse are assessed in the interim analysis.
- Discussing which of the results/effects are patient relevant and how to best visualise the data.	- We will report all outcomes independent of the assessed effect. The objective of this exchange is to receive feedback which effects are most relevant for patients and how data should be visualised so that they can be understood easily
- Advise on which form the results should be communicated.	- Assuring that the study results are disseminated to a wide audience.
- Support the communication of results in lay language.	- Ascertaining that the study results can be communicated to participating patients and the general public.
- Discussing the impact and the next steps with the researchers.	- Making sure that appropriate action is taken based on the study results taking into consideration the patient perspective.
- Evaluating the PPI involvement	- Learning how best to involve PPI in future collaborative research projects

6.3 Methods of minimising bias

6.3.1 Randomisation

Participants will be allocated 1:1 to either linezolid or placebo for 5 days using block randomisation with varying random block sizes of 2 or 4 implemented in the data capture system REDCap®. For more details see chapter 7.3 *Assignment to study groups*.

6.3.2 Blinding procedures

The treatment allocation will not be revealed to any study staff or participants (i.e., physicians, study nurses, participants, outcome assessors). Only the unique randomization number will be visible for blinded personnel. The randomisation list will only be accessible to designated staff from Spital-Pharmazie Basel for manufacturing,

labelling and blinding the study medication, and the statistician who will prepare the randomisation list and upload it in REDCap®. The study medication will be packaged and dispensed by Spital-Pharmazie Basel to ensure that all participants, treating physicians, clinical investigators and study nurses involved in the study (including outcome assessors) will remain blinded to treatment allocation throughout the study.

6.3.3 Other methods of minimising bias

Not applicable.

6.4 Unblinding Procedures (Code break)

The study medication (10 tablets per container) with a unique randomisation number will be distributed to the study centres. For each unique randomisation number, the study centre will receive a sealed opaque envelope with the information if the container contains linezolid or placebo. The treating physician will only be allowed to open an envelope if safety concerns require knowing a participant's allocated intervention. All unblinding procedures must be entered within the eCRF. The envelopes will be checked during monitoring visits and after the completion of the study.

7. STUDY POPULATION

7.1 Eligibility criteria

Inclusion criteria:

- *S. aureus* grown from at least one blood culture
- Hospitalised at a participating centre
- ≥18 years old
- Written informed consent or fulfilling criteria for an emergency exception from informed consent requirements

Exclusion criteria:

- Administration of the initial drug treatment not feasible within 72 hours since the collection of the first positive blood culture with *S. aureus*
- Documented history of positive blood cultures for *S. aureus* occurring between 72 hours and 180 days prior to the eligibility assessment
- Necrotising fasciitis
- Currently receiving linezolid or clindamycin
- Use of any monoamine oxidase A or B inhibitor within the last two weeks
- Known hypersensitivity to linezolid or any other ingredients of the study drugs
- Current severe thrombocytopenia (i.e. $<30x10^9/l$)
- Oral application of study drug not possible (per mouth or per gastric tube)
- Currently breastfeeding
- Local treating team believes that death is imminent and inevitable
- Patient is receiving end of life care and antibiotic treatment is not considered appropriate
- Local treating team believes that participation in the study is not in the best interest of the patient
- Any indication that the patient is unwilling to participate in the study including an advance directive stating such unwillingness

General:

Patients taking serotonergic drugs such as selective serotonin reuptake inhibitors (SSRI) or tricyclic antidepressants are not generally excluded. See detailed information on risk assessment and precautions in chapter 8.6 *Trial specific preventive measures*.

Pregnant women will not be excluded from study participation per se, as linezolid is not formally contraindicated during pregnancy. Limited data exist on the treatment of pregnant women with linezolid, mainly from clinical trials in treating multidrug-resistant tuberculosis. In 31 pregnancies under linezolid exposure, none reported foetal toxicity.[95] In the event of pregnancy, the treating physician and the patient (or the patient's next of kin) will assess whether the potential benefits outweigh the potential risks to the foetus.

Linezolid has been shown to pass into breast milk and accordingly, breastfeeding should be discontinued prior to and throughout administration of linezolid.

7.2 Recruitment and screening

We anticipate that patients will be recruited at the infectious disease departments of these 12 Swiss hospitals:

- Basel, University Hospital Basel (Sponsor Investigator centre)
- Aarau, Cantonal Hospital Aarau
- Basel, St. Clara Hospital Basel
- Bern, University Hospital Bern
- Delémont, Cantonal Hospital Jura

- Lausanne, University Hospital Lausanne
- Lugano, Cantonal Hospital Lugano
- Geneva, University Hospital Geneva
- St. Gallen, HOCH Health Ostschweiz, Cantonal Hospital St. Gallen
- Winterthur, Cantonal Hospital Winterthur
- Zürich, University Hospital Zürich
- Zürich, City Hospital Zürich

Patients will be recruited during their hospitalisation at participating centres. Patients will be flagged as soon as *S. aureus* is detected in a blood culture. The time required from blood collection to the confirmation of *S. aureus*, typically ranging from 12 to 36 hours, will depend on factors such as the time to positivity and the method employed for *S. aureus* identification.[108] The results will be immediately reported to the study team according to each centre's infrastructure - either via an automated electronic alarm system or via verbal communication from the clinical microbiology laboratory. Infectious disease specialists, serving as the local principal investigators at all participating centres, are routinely involved in the care of patients with *S. aureus* bacteraemia. Their involvement is well established and recognized for its positive impact on patient outcomes.[109-112] Patients with blood cultures positive for *S. aureus* will be immediately screened for eligibility using the inclusion and exclusion criteria by the local PI or delegated physician. Eligible patients will be approached by the local study team as soon as possible. They will be informed about the LIPS trial via verbal and written study explanation, which consists of a printed summary in lay language including details of the intervention and potentially associated benefits and risks. Each participant will receive a consent form offering sufficient details to enable them to make an informed decision about their participation. For patients unable to give informed consent due to severe illness or other circumstances, it must be checked if a statement of wishes formulated in a state of capacity is available. If no such statement if available, an appropriate substitute (i.e., a close relative or a physician not involved in the research project) may make decisions based on the presumed wishes and the best interests of the patient as recommended by Swissethics.[113] Participants who were included after receiving consent from their next of kin or confirmation by an independent physician will be asked to provide retrospective consent as soon as their health status allow an informed decision.

The randomisation and intervention will start immediately upon the receipt of a formal written consent. Participants will not receive any financial compensation for their participation. The participating centres will receive financial reimbursement for establishing the local trial infrastructure and workflow as well as a reimbursement per participant to compensate for the costs of the study staff and other resources.

The sponsor notifies the CEC and the CA of the first study participant, in accordance with Art. 23a ClinO. If the first participating person is not included in the trial within two years following the issuance of the last authorization, the trial is considered interrupted. The clinical trial may then not be commenced until an application for an extension of the time limit has been approved. The application for the extension is submitted to the CEC, and to CA as a substantial amendment.

7.3 Assignment to study groups

Participants will be allocated 1:1 to either linezolid for 5 days or placebo for 5 days. The randomisation will be stratified by centre and ICU status at time of enrolment and done using block randomisation with varying random block sizes of 2 or 4. The trial statistician will generate the randomisation list outside of the data capture system REDCap®. This list will then be uploaded into the data capture system. The goal is to randomise participants such that the groups are as balanced as possible with respect to study centre and ICU status at time of enrolment.

After informed consent has been obtained and all eligibility criteria have been reviewed and met, the local investigator (or designated staff) enters the minimal required data into REDCap® (i.e., participant ID, year of birth, if participant is on ICU). The allocation is done by REDCap® which will display a unique, randomisation number that remains the same throughout the study. Each randomisation number will correspond to a drug container which is labelled with the unique randomisation number.

7.4 Criteria for withdrawal / discontinuation of participants

Participants can withdraw from the study at any time without providing a reason. Participants who discontinue the intervention will still be encouraged to complete follow-up assessments. Reasonable effort should be made to

establish the reason for discontinuation while fully respecting the participant's rights so reasons may be analysed. In case of adverse events, the treating physician decides (if possible, together with participants or close relatives) whether the study drug must be discontinued. Outcomes of these participants will still be collected, and they remain in the intention-to-treat analysis as randomised.

Participants who were included after receiving consent from their next of kin or confirmation by an independent physician will be asked to provide retrospective consent as soon as their health status allow an informed decision. If the participant refuses to give this retrospective consent, we will not collect any further data. However, we will analyse the data that has been collected up to this timepoint, as excluding these data could bias the analysis.

8. STUDY INTERVENTION

8.1 Identity of Investigational Medicinal Products

8.1.1 Experimental Intervention

The IMP in this study is linezolid 600 mg tablets (Linezolid STADA®) as described in chapter 3.2 *Investigational Medicinal Product (treatment) and Indication*. The IMP will be administered orally from day 1 until day 5, twice daily.

8.1.2 Control Intervention (standard/routine/comparator treatment)

Participants in the control arm will be receiving oral placebo tablets. Procedures regarding route of administration, study treatment duration and treatment phases will be identical in the IMP- and the placebo-group (see chapter 8.1.1 *Experimental Intervention*).

8.1.3 Packaging, Labelling and Supply (re-supply)

Linezolid STADA® and placebo tablets will be provided as trial supplies to the study centres re-packaged in identical opaque containers by Spital-Pharmazie Basel. Each container will contain 10 tablets of either linezolid 600 mg or placebo. The containers will be labelled identically except for the unique randomisation number on each container. Spital-Pharmazie Basel will be responsible for the labelling, packaging, and shipping of the IMPs. Each centre will be supplied with a minimum number of containers at site initiation. The label contains the information required by the authorities.

The trial manager will monitor the recruitment progress of the individual study centres and will inform Spital-Pharmazie Basel if re-supply is needed. Spital-Pharmazie Basel will arrange for the shipment of the re-supply to the centres following instructions by the trial manager.

8.1.4 Storage Conditions

The IMP must be kept in a secure, limited access storage area under the recommended storage conditions (i.e. identical storage conditions apply as for linezolid tablets that are used in routine practice at the study centre).

8.2 Administration of experimental and control interventions

8.2.1 Experimental Intervention

One linezolid 600 mg tablet will be administered orally twice a day on 5 consecutive days (chapter 3.4 *Clinical Evidence to Date*). For hospitalised participants, the study drug will be distributed in the same manner as a participant's other oral medication. The local investigator or delegated staff will hand over the study drug to the nursing team who will distribute the study drug to the participant according to the instructions by the physician and according to the site-specific local procedures. In general, each dose/each tablet will be distributed separately to the participant and only just before intake. If, based on the treating team's assessment, a participant is unable to swallow the study medication, tablets may be crushed and resuspended for easier consumption or for administration via a nasogastric tube according to clinical routine and following site-specific SOPs.

In the unlikely event that participants are discharged home before day 5, they will receive the remaining study medication along with detailed instructions on how and when to take them at home.

8.2.2 Control Intervention

Dosing and administration of the placebo tablets are the same as for participants in the experimental intervention arm.

8.3 Dose modifications

Drug-related AEs leading to discontinuation of the drug are expected to be extremely rare when linezolid is used for 5 days. Discontinuation of IMP may occur in the situations listed in chapter 6.4 *Unblinding Procedures (Code*

break) and chapter 7.4 *Criteria for withdrawal / discontinuation of participants*. The study medication will be given twice a day for 5 days (in total 10 administrations) with approx. 12 hours interval between each administration. If maintaining a fixed 12-hour interval is challenging due to the start time (e.g. if subsequent doses would need to be administered in the middle of the night), the treating physician may adjust the first dosing interval according to standard practice. However, the interval should not be shortened or extended by more than 6 hours. Further dose modifications are not allowed.

8.4 Compliance with study intervention

The intake of the study medication will be documented for each tablet (date and time) either electronically in the participant's eCRF or directly on a separate leaflet (which will be later transferred to the eCRF). Any missed doses, along with the reasons for missing them, must also be documented. The participant will receive instructions for the correct storage and administration of IMP at the ward if discharge occurs before completion of the 5-day intervention phase.

8.5 Data Collection and Follow-up for withdrawn participants

Participants can discontinue the trial intervention at any time without providing a specific reason. Participants who wish to withdraw from the study will commonly receive further measurements as part of clinical routine. We will ask these participants if we are allowed to collect the corresponding routine clinical data and/or to contact them on day 90 to assess the primary outcome.

8.6 Trial specific preventive measures

Thrombocytopenia is a common adverse event of linezolid, but it is usually associated with treatment durations of 10 or more days. Given the lifespan of thrombocytes and the short intervention duration of five days in our study, we expect a low risk of linezolid-mediated thrombocytopenia. However, thrombocytopenia is also commonly seen in participants undergoing sepsis. If participants develop severe thrombocytopenia during the intervention, the local investigator should assess if this may be associated with the trial medication and decide on stopping or continuing the intervention for this participant.

Linezolid should not be used in patients taking any medicinal product which inhibits monoamine oxidases A or B (e.g. the antidepressant moclobemide or the Parkinson’s medications rasagiline and safinamide) or within two weeks of taking any such medicinal product.

For participants taking serotonergic agents, the local investigator should evaluate if pausing the serotonergic medication for the duration of the trial is possible. If not, the local investigator (or delegate) should decide if the participant can be included ensuring that potential signs of serotonin toxicity are monitored for the duration of the study intervention phase, or if the patient should be excluded. On trial days 1-5, clinical observation for signs of serotonin toxicity is required for participants taking any of the following medications: serotonin re-uptake inhibitors, tricyclic antidepressants, serotonin 5-HT1 receptor agonists (triptans), directly and indirectly acting sympathomimetic agents (including the adrenergic bronchodilators, pseudoephedrine and phenylpropanolamine), vasopressive agents (e.g. epinephrine, norepinephrine), dopaminergic agents (e.g. dopamine, dobutamine), pethidine or buspirone.

8.7 Concomitant Interventions (treatments)

All participants receive standard of care treatment for *S. aureus* bacteraemia as clinically indicated and according to local and international recommendations. This includes targeted antibiotic treatment for at least 2 to 6 weeks, mostly applied intravenously. The choice of standard of care antibiotic will not be affected by the study. In case clindamycin is administered after enrolment, this must be documented in the eCRF.

8.8 Study Drug Accountability

Study drugs will be accurately and adequately monitored from shipment to the site until return or disposal. Dates of receipt/expiry/use/return will be recorded.

8.9 Return or Destruction of Study Drug

Study sites will destroy the left-over study medication according to site-specific SOP. In the rare cases that participants are discharged from the hospital before completing the study medication, they will be provided with the necessary tablets to continue their treatment at home until the 5-day course is completed. They will be instructed to return any unused tablets (e.g., if they are unable to take a dose for any reason) to a pharmacy for proper disposal.

Docusign Envelope ID: 7FB1D507-0860-4709-95DA-14CC8893F28B

9. STUDY ASSESSMENTS

9.1 Study flow chart/ table of study procedures and assessments

Study Schedule

	Screening	Enrolment	Intervention phase					In-Hospital Follow-Up (applicable only if participant remains hospitalised)				Close-out
**Time point**	**0-72h before randomisation**		**Day 1**	**Day 2**	**Day 3**	**Day 4**	**Day 5**	**Day 7**	**Day 14**	**Day 28**	**Discharge day**	**Day 90**
S. aureus positive blood culture	X											
Identification of patient		X										
Eligibility checks		X										
Informed consent		X										
Randomisation			X									
**IMP administration**			X	X	X	X	X					
**Time point**	**0-72h before randomisation**		**Day 1 (-1 day)**	**Day 2**	**Day 3 (±1 day)**	**Day 4**	**Day 5 (±1 day)**	**Day 7 (±1 day)**	**Day 14 (±3 days)**	**Day 28 (±4 days)**	**Discharge day**	**Day 90 (±7 days)**
**Clinical assessments**												
Baseline data			X[1]									
SIRS criteria							X					
All-cause mortality			Assessed until day 90									
Clinical failure										X	X	X
Length of hospital stay											X	
Length of ICU stay											X	
Antibiotic and/or surgical treatment												X
**Laboratory assessments**												
Blood cultures				X			X[2]					
Creatinine			X				X		X[3]			
CRP			X				X					
ALT and gGT			X				X		X[3]			
Blood count (Hb, Tc, Lc)			X		X		X	X				
Linezolid trough conc.							X[4]					
Microbiological failure										X	X	X
New antibiotic resistance												X
**Patient reported outcomes**												
Quality of life (SF-36)												X
Return to usual level of function												X
**Safety assessments**												
Serotonin toxicity, myelosuppression, hyperlactatemia[5]			X	X	X	X	X	X				
Acute kidney injury[5]							X		X			
C. difficile - associated diarrhoea[5]												X
SAE / SAR			Collected until day 90									

Abbreviations: S. aureus – Staphylococcus aureus; SAE – Severe Adverse Event; SAR – Severe Adverse Reaction; SIRS - Systemic Inflammatory Response Syndrome; ICU – Intensive Care Unit; CRP – C-Reactive Protein; ALT – Alanine Transaminase; gGT – Gamma-glutamyl Transferase; Hb – Haemoglobin; Tc – Thrombocytes; Lc – Leukocytes; SF-36 – Short Form-36

[1] Baseline data include: date of hospital admission, index blood culture, linezolid susceptibility, ethnicity, vital signs, comorbidities, standard antibiotic treatment, markers of severity (mechanical ventilation, cardiac arrest, use of vasopressors, ICU), injection drug use, mental health diagnosis.
[2] Assessment of persistent bacteraemia. Only required if day 2 blood culture is positive or recorded if done as part of standard of care.
[3] Choose the result that represents the highest level recorded between day 6-14.
[4] Linezolid trough concentration is only measured in a subset of participants in participating centres.
[5] Adverse events of special interest until day 7 include: serotonin toxicity, myelosuppression, hyperlactatemia; until day 14: acute kidney injury; until day 90: C. difficile-associated diarrhoea.

9.2 Assessments of outcomes

9.2.1 Assessment of primary outcome

The primary outcome utilises the Desirability of Outcome Ranking (DOOR) and is comprised of the following 4 assessments:

i) "Alive on day 90" and "return to usual level of function by day 90" will be documented by local investigators or delegated staff. Participants who did not die during hospitalisation will be called on day 90 (±7 days). If a routine patient visit coincides with this time point, the assessment may be done in person. If the participant can't be reached, the local investigator or designated personnel will make at least 5 attempts at different times of the day to reach the participant. If these fail, reasonable attempt will be made to acquire the information from relatives, caregivers, or the participant's emergency contact or primary care physician.

ii) During the phone call at day 90 (±7 days), the participant will be asked to rate their level of function in the 4 weeks prior to their bacteraemia and their current level of function from 6 to 1 according to the modified functional bloodstream infection score[104]:

6: Out of hospital, able to complete daily activities without assistance (no limitations)

5: Out of hospital, able to complete daily activities without assistance but with some limitations (e.g. slow, pain)

4: Out of hospital, unable to complete daily activities without assistance

3: Out of hospital; significant disability; requires a high level of care and assistance daily (this includes residential aged care and nursing homes)

2: Hospitalised (or equivalent)

1: On palliative care in terminal phases of life (in hospital or at home)

Baseline is defined as the best score within the 4 weeks before randomisation. If the participant is alive but unable to answer the questions, their caregiver, relatives, or primary care physician will answer the questions to their best knowledge.

iii) "Complications" will be assessed by evaluation of the participant during hospitalisation either by direct observation or by laboratory assessments. The data are accessed from hospital information systems and entered into the RedCap® database by the local investigator or designated personnel.

a. Microbiological failure: Any positive sterile site culture with *S. aureus* between 14 and 90 days after randomisation. A sterile site means any site of the body where microorganisms are usually absent, i.e. below the outer and inner colonised surfaces of the skin and mucous membranes. Positive sterile sites cultures include deep visceral and musculoskeletal abscesses obtained in a sterile manner.
b. Clinical failure: Newly identified focus of *S. aureus* between 14 and 90 days after randomisation as determined by the site investigator. This can incorporate clinical, radiological, microbiological, and pathological findings.
c. Treatment change: This includes any type of adaptation of the treatment such as new intervention or surgery, or re-start, prolongation or change of antibiotic treatment initiated and documented by the treating physician.
d. Serious adverse reactions are assessed and documented by the treating physician.
e. Any adverse event (irrespective of grade) leading to study drug discontinuation is documented by the treating physician.

"Complications" a and b will be routinely captured in the eCRF by the local investigator (or a delegated physician), based on hospital information system data on day 28, 42, and 90 and at the day of hospital discharge. An independent and blinded outcome assessment board will review all microbiological and clinical failures to ensure consistency.

On day 90, information on treatment changes ("complication" c) will be assessed from hospital information system data. SAE ("complication" d) will be documented as soon as an investigator becomes aware of a SAE (see chapter 10.1.1 *Definition and assessment of (serious) adverse events and other safety related events*). For all serious adverse events (SAEs), the independent and blinded data safety monitoring board (DSMB) will review any suspected relation to the study drug (defining the SAE as an SAR). In cases where the participant is transferred to another hospital, the local investigator must contact the new hospital to assess the hospital length of stay and potential SAE. Adverse events ("complication" e) will be assessed

as outlined in chapter 9.2.4 *Assessment of safety outcomes.*

iv) "Hospital length of stay" corresponds to the duration of the acute hospital stay from the day of randomisation into the LIPS trial until day of hospital discharge. These data are assessed using hospital information system data. In case participants are transferred to another acute care hospital ("new hospital"), the local investigator (or delegated personnel) must contact the "new hospital" to receive the information when the participant was discharged from the "new hospital". Outpatient parenteral antibiotic treatment or transfer to rehabilitation centres are not part of the acute hospital stay.

9.2.2 Assessment of secondary outcomes

The secondary outcomes include each part of the DOOR outcome separately as described in chapter 9.2.1 *Assessment of primary outcome* and time to death up to day 90.

Further secondary outcomes will be assessed as follows:

<u>Clinical outcomes:</u>

- Time to being discharged alive (assessed until day 90)

  In the eCRF, we collect the day of hospital discharge (see 5.1 *Primary Outcome* for detailed definition of primary outcome). For participants who die during the hospitalisation, 90 days will be recorded.

- Number of days without being on the Intensive Care Unit (ICU) up to day 90 (i.e. mean number of days being alive without being on the ICU between randomisation until day 90).

  After day 90 we will collect from the hospital information system data how many days the participant spent on the ICU. Together with the available data on mortality, we will calculate the "Number of days without being on the ICU".

<u>Infection dynamics and control:</u>

Information on these outcomes is collected from the hospital information system data.

- Persistent bacteraemia: positive blood culture on day 5 (±1 day) after randomisation. If blood culture on day 2 or 3 is negative, then day 5 blood culture will be assumed to be negative (in case it is not carried out).
- Two or more systemic inflammatory response syndrome (SIRS) criteria on day 5 after randomisation. This includes: 1. Abnormal body temperature (<36°C or >38°C), 2. tachypnoea or mechanical ventilation (RR >20 breaths per minute), 3. tachycardia (HR >90 beats per minute in an adult, 4. abnormal leukocyte count (from routine blood sampling on day 5 ±1 day, defined as >12.0 x 10^9/L or <4.0 x 10^9/L or >10% of immature (band) forms).
- Change in C-reactive protein (CRP) from randomisation until day 5 (±1 day). Day 1 CRP means any blood CRP measurement taken on randomisation day 1 or the calendar day prior to randomisation. If there is more than one measurement, the value recorded is the one taken closest before randomisation.
- Early clinical treatment failure between 5 and 13 days after randomisation leading to treatment change. Newly identified focus of *S. aureus* between 14 and 90 days after randomisation as determined by the site investigator. This can incorporate clinical, radiological, microbiological, and pathological findings.
- Early microbiological treatment failure between 5 and 13 days after randomisation leading to treatment change. Any positive sterile site culture with *S. aureus* between 5 and 13 days after randomisation. A sterile site means any site of the body where microorganisms are usually absent, i.e. below the outer and inner colonised surfaces of the skin and mucous membranes. Positive sterile site cultures include deep visceral and musculoskeletal abscesses obtained in a sterile manner.
- Development of any new antibiotic drug resistance in *S. aureus* until day 90. This includes any new resistance absent in the *S. aureus* from the initial blood culture and detected in any *S. aureus* cultured after the start of the intervention.
- Days alive and free of antibiotics up to day 90
- At hospital discharge, the number of days a patient received antibiotics will be recorded. At day 90, an additional check will be conducted in case additional antibiotics were prescribed after hospital discharge.

Patient-reported outcomes:

SF-36 questionnaire:[105-107] This represents a validated patient-reported outcome measure and will give more detailed information of the patient's physical, mental, emotional, and social well-being. Participants will receive this questionnaire on day 90 after randomisation either via mail or E-mail (based on participant preference). The questionnaire will be available in German, English, French, and Italian. From the SF-36, the following two outcome measures are derived:

- Physical health at day 90 (measured with the SF-36 questionnaire, Mental Component Summary)
- Mental health at day 90 (measured with the SF-36 questionnaire, Physical Component Summary)

9.2.3 Assessment of other outcomes of interest in a subset of participants

The following other outcomes of interest will only be collected at participating study centres and therefore will only be collected from a subset of trial participants.

- Linezolid trough plasma concentration at day 4 or 5
- Linezolid minimal inhibitory concentration of the respective *S. aureus* strain from the blood

Measurement of linezolid trough levels is an established assay that is routinely performed by the USB diagnostic laboratory and can be ordered from the standard catalogue of analyses. The laboratory will perform linezolid trough analyses in batches on plasma samples collect and frozen by the participating centres (see 9.3.10 *Measurement of trough levels of linezolid in a subset of participants (day 4-5)*).

For the same participants, the minimal inhibitory concentration (MIC, in mg/l) of linezolid for the respective *S. aureus* strain will be measured according to microbiological standard procedures used for clinical routine. In general, this will be done by the gradient diffusion method (Etest).[114]

9.2.4 Assessment of safety outcomes

9.2.4.1 Adverse events

Adverse events that will be documented by the local investigator or a delegated physician are the following:

Adverse events of special interest:

- Clinical signs of serotonin toxicity until day 7

  Serotonin toxicity will be assessed during clinical routine. In case of clinical signs of serotonin toxicity, the Hunter criteria and diagnostic algorithm will be used (including spontaneous or inducible clonus, ocular clonus, tremor, hypertonia and hyperthermia, agitation or diaphoresis, hyperreflexia). Written instructions on how to assess the hunter criteria (only in case of clinical suspicion of serotonin toxicity) are provided in the appendix, chapter 18.2 *The Hunter Serotonin Toxicity Criteria: for diagnosing serotonin syndrome*.

- Laboratory signs of myelosuppression until day 7

  Participants will be monitored for signs of myelosuppression (thrombocytopenia, anaemia, leukopenia) as part of the clinical routine blood sampling and graded according to the Common Terminology Criteria for Adverse Events (CTCAE) of the US National Institute of Health/National Cancer Institute [115]. This includes for thrombocytopenia grade 1: platelet between lower limit of normal – 75 x 10^9/L, grade 2: 75 – 50 x 10^9/L, grade 3: 50-25 x 10^9/L, grade 4, < 25 x 10^9/L; for anaemia: grade 1: haemoglobin between lower limit of normal – 100 g/l, grade 2: < 100-80 g/l, grade 3: < 80 g/l, grade 4: life-threatening consequences; urgent intervention indicated, grade 5: death; and for leukopenia: grade 1 leukocytes between lower limit of normal – 3.0 x 10^9/L, grade 2: <3.0 – 2.0 x 10^9/L, grade 3: <2.0 – 1.0 x 10^9/L, grade 4: < 1.0 x 10^9/L.

- Evidence of hyperlactatemia until day 7

  In case of a clinical suspicion of hyperlactatemia, local investigators must collect a blood sample to assess if the lactate level is above the upper norm according to specific laboratory-defined reference ranges. For increased lactate levels, the causality with the study treatment will be assessed (see chapter *10.1.1 Definition and assessment of (serious) adverse events and other safety related events*).

- Acute kidney injury until day 14

  Acute kidney injury according to the modified Kidney Disease Improving Global Outcomes (KDIGO) stage 1 is defined as an increase in serum creatinine of ≥26.5 µmol/L from randomisation (baseline) to study day 5 or an increase in serum creatinine by 1.5 times or more the level of randomisation within 14 days of

randomisation. To collect this outcome, the serum creatinine at baseline (on randomisation day 1 or the calendar day prior to randomisation), day 5 (±1 day), and only if participant remains hospitalised, at day 14 (±3 days) will be documented in the eCRF. The values will be captured from available data in the hospital information system or from the participant's primary care physician, respectively.

- *C. difficile*-associated diarrhoea within 90 days.

  The treating clinicians will assess for *C. difficile* infection based on the centre-specific diagnostic algorithm if clinical suspicion arises due to diarrhoea during antibiotic treatment. *C. difficile* infection will be evaluated on day 14, at discharge, and on day 90 by reviewing the participant's electronic health records. If a participant reports severe diarrhoea during a 90-day follow-up phone call, occurring after discharge and managed outside the centre, the responsible physician may be contacted to determine if a *C. difficile* infection was diagnosed.

9.2.4.2 Laboratory parameters

Blood sampling will be done according to clinical routine. Laboratory-specific adverse events will be measured as part of the clinical routine as outlined above. No additional tissue sampling or body fluids are necessary.

9.2.4.3 Vital signs

Vital signs are measured according to clinical routine. No additional study-specific vital sign measurements are necessary.

9.2.5 Assessments in participants who prematurely stop the study

Participants who withdraw from the study will receive further measurements as part of clinical routine. This includes all relevant assessments of adverse events.

9.3 Procedures at each visit

All visits except the 90-day follow-up will take place during the index hospitalisation and will be integrated or part of the clinical routine management of the participants.

9.3.1 Screening and identification of patients (Up to 72 hours before randomisation)

In patients suspected of having a bloodstream infection, blood is routinely drawn into blood culture bottles. These bottles are sent to the respective centre-specific laboratory for diagnostic microbiology to be incubated at approximately 37°C. When growth is detected in the bottles, pathogen identification diagnostics are initiated according to the laboratory-specific work instructions. If it is a case of *S. aureus* bacteraemia, blood culture bottles typically become positive (detection of bacterial growth in the bottle) after about 9 hours to 1 day of incubation.

Eligible patients will be identified by having positive blood cultures with *S. aureus*. Therefore, screening for eligible patients will primarily be done through regular review of the results of blood culture results sent to the diagnostic microbiology laboratory of each centre. Each centre will implement centre-specific procedures to ensure prompt notification of a positive blood culture result with *S. aureus.* This can occur through an electronic alert system or through other communication channels, depending on what is available and practical. Infectious diseases specialists of each centre are routinely involved in all patients with *S. aureus* bacteraemia. The goal is to identify potential participants as quickly as possible. Any microbiological identification method to identify *S. aureus* used in routine practice can be applied to positive blood cultures: PCR (polymerase chain reaction) results as well as other methods (e.g., MALDI-TOF: Matrix-Assisted Laser Desorption/Ionization-Time of Flight) are acceptable. PCR results (usually part of commercially available multiplex panel PCR) are typically available about 3 hours after detecting growth in the blood cultures; without PCR, pathogen identification is usually available within 24 hours.

9.3.2 Enrolment of participants (0-72 hours before randomisation)

Once a patient with *S. aureus* bacteraemia has been identified, the responsible centre-specific study team will assess whether the patient meets the inclusion criteria for the study. If this is the case, the site investigator or a delegated and study-specific trained person will approach the hospitalized patient. The patient will be informed

about the study and asked if they wish to participate. The patient should be given time to discuss any questions or uncertainties they may have. The patient will receive the informed consent form along with written explanations about the purpose and procedures of the study. Once the patient agrees to participate in the study, they will have to provide written consent by signing the appropriate form.

If a patient is unable to give an informed consent due to medical reasons (e.g., lack of consciousness due to severity of illness), a family member or a study-independent, treating physician should decide on the inclusion in the study on behalf of the patient. If the patient can be included, the patient representative should sign the corresponding informed consent form. When such a participant regains the ability to make decisions, the study inclusion will be reviewed with them by the study team, and a new informed consent will be signed. The participant may choose to withdraw from the study at any point.

The study team informs the care team (doctors, nursing staff) about the participant's participation in the study. Each centre will establish its own process how to distribute this information.

9.3.3 Randomisation (day 1)

Once a participant is enrolled via informed consent, the participant will be randomly assigned to a study arm through electronic randomisation within the REDCap® database. This will be initiated and coordinated by the local investigator (or designated personnel). All involved persons (participant, treating team, study team) will remain blinded to the allocation throughout the entire study duration – except in the case of emergency unblinding (see chapter 6.4 *Unblinding Procedures (Code break)*)

9.3.4 Assessment of baseline characteristics (day 1)

Most study-specific baseline characteristics are assessed as part of the clinical routine and can be extracted from the patient charts of each centre. The baseline characteristics include date of hospital admission, index blood culture, linezolid susceptibility, ethnicity, vital signs, polymicrobial bacteraemia (defined as more than one organism [at species level] in the index blood cultures, excluding those organisms judged to be contaminants by the treating clinicians), comorbidities, standard antibiotic treatment, markers of severity (mechanical ventilation, cardiac arrest, use of vasopressors, ICU), labour force status, injection drug use, and mental health diagnosis. The data closest to the time before start of the study treatment should be used. The study team of each site will check at the time of randomisation if all relevant information is available from the clinical routine assessment and – if necessary – will add the missing information by contacting the treating team or the participant directly. Together with the participant, the local investigator (or a delegated physician) will assess the level of function 4 weeks before randomisation. In case the participant is not able to provide this information, this data will be assessed at day 90 (asking about their level of function 4 weeks before their *S. aureus* bacteraemia).

9.3.5 Treatment (day 1 – day 5)

Each centre will implement the trial-specific procedures in accordance with local circumstances to ensure the administration and documentation of the study medication. The study medication should be prescribed and handled by the medical team in the same way as other oral medication during hospitalisation. The study team will hand over the study medication to the responsible medical treatment team on the respective ward. The study medication will be clearly labelled and stored in a designated location according to the centre's guidelines and prespecified workflow for the study. The study medication should be administered alongside the participant's other medications during hospitalization, generally by the nursing staff. The study medication should be administered as soon as possible once a participant has been enrolled and randomised. Thus, the first dose may also be given at night or at any time during the day. The study medication will be given twice daily for five days (in total 10 administrations) with approx. 12 hours interval between each administration. If maintaining a fixed 12-hour interval is challenging due to the start time (i.e., if subsequent doses would need to be administered in the middle of the night), the treating physician may adjust the interval according to standard practice. However, the interval should not be shortened or extended by more than 6 hours. It must be ensured that the start and stop of the treatment, the timing of each individual administration, and any missed doses are documented and can be traced for each participant. If a dose was not administered, the reason for this should be documented.

Most study-specific baseline characteristics are assessed as part of the clinical routine and can be extracted from the patient charts of each centre. The baseline characteristics include date of hospital admission, index blood culture, ethnicity, vital signs, comorbidities, standard antibiotic treatment, markers of severity (mechanical ventilation, cardiac arrest, use of vasopressors, ICU), injection drug use, and mental health diagnosis. The data

closest to the time before start of the study treatment should be used. The study team of each site will check at the time of treatment start if all relevant information is available from the clinical routine assessment and – if necessary – will add the missing information by contacting the treating team or the participant directly.

9.3.6 Assessment of routine laboratory measurements (day 1-7)

Laboratory assessments at start and during treatment will be part of the clinical routine. Either on the day before or on the day of study treatment start, participants will receive routine blood sampling which includes blood count (Hb, Tc, Lc), measurement of renal function (creatinine) and liver function (ALT, gGT), as well as inflammatory parameter (CRP). The study team will review the blood samples at start and during treatment and will re-order any missing laboratory measurements from the collected blood samples if, in rare cases, the necessary measurements for the study according to the visit plan were not routinely performed. No additional blood should be drawn to assess clinical routine measurements. The blood count should be measured 4 times between day 1 (-1) and day 7 (±1), creatinine and CRP at least on day 1 and day 5 (±1), creatinine also on day 14 (±3) if a participant is still hospitalised.

9.3.7 Subproject *Molecular mechanisms of S. aureus bacteraemia* (day 1 until 48h after the patient's first negative blood culture)

Only for a subgroup of patients included in Basel, Switzerland.

Blood will be used to determine the location of *S. aureus* in the blood (intra- or extracellular).

For this, a maximum of one additional 7.5 ml EDTA tube of blood per day will be collected from day 1 until follow-up blood cultures remain negative for 48 hours.

- For patients who consented to further analysis of *S. aureus* in their blood, blood samples will be collected daily. If no routine blood work is performed on an eligible day, an additional blood draw should be performed to collect the sample.
- For patients enrolled via the confirmation of an independent physician under emergency exception from informed consent requirements, blood samples will only be collected during routine blood drawing.
- For patients who refused consent to further analysis in the blood, no additional blood samples for the subproject will be taken.

9.3.8 Follow-up blood cultures (day 2, day 5 ±1)

Follow-up blood cultures are routinely performed and repeated in *S. aureus* bacteraemia until blood cultures become negative. This belongs to the guidelines-supported management of such infections. At day 2 after trial enrolment one further pair of blood culture should be drawn. This needs to be ordered by the treating physician. If the prior blood cultures were still positive for *S. aureus*, then these follow-up blood cultures will be routinely ordered and drawn. If the prior blood cultures were negative, then the blood cultures at day 2 need to be ordered additionally to the clinical routine. If day 2 blood culture stayed positive, then further follow-up cultures are mandated at day 5 (±1) and will be part of the clinical routine as outlined above.

9.3.9 Assessment of SIRS criteria (day 5 ±1)

The components of the SIRS criteria (abnormal body temperature, mechanical ventilation, tachycardia, and abnormal leucocytes) are routinely assessed and documented during clinical management of the hospitalised participants. The vital parameters (body temperatures, respiratory rate, pulse) are usually measured at least once daily. Leukocytes are usually measured every 1-3 days. Tachypnoea is assessed when clinically indicated. Thus, these criteria will be collected from the hospital information system data.

9.3.10 Measurement of trough levels of linezolid in a subset of participants (day 4-5)

Participating centres will collect one 7.5 ml Monovette® Li-Heparin from participants between day 4 and 5 after randomisation for a representative linezolid trough measurement. Plasma sampling may be combined with the routine blood draw but should be timed immediately before the next linezolid dose.

Plasma should be isolated by centrifugation and frozen as soon as possible after blood draw, but at the latest within 6 hours. Samples will be stored at -80°C until shipment. Periodically, samples are shipped on dry ice to the University Hospital Basel where linezolid trough levels will be analysed by the diagnostic laboratory.

9.3.11 Assessment of adverse events of special interest (day 1-5, day 14 ±3, discharge day)

- Clinical signs of serotonin toxicity until day 7: Serotonin toxicity will be assessed during clinical routine. Participants will routinely be visited daily by the treating physician. Daily measurement of vital signs including blood pressure is part of the clinical routine. In case of clinical signs of serotonin toxicity, the Hunter criteria and diagnostic algorithm will be used (including spontaneous or inducible clonus, ocular clonus, tremor, hypertonia and hyperthermia, agitation or diaphoresis, hyperreflexia). In case of clinical signs of serotonin toxicity, the IMP administration will be halted. The local investigator will be involved to re-assess the clinical picture and to confirm or reject the observation. The local investigator will then decide if the participant can continue taking the IMP or needs to stop the intervention.
- Laboratory signs of myelosuppression until day 7: Participants will be monitored for signs of myelosuppression (thrombocytopenia, anaemia, leukopenia) as part of the clinical routine blood sampling. If blood count shows signs of myelosuppression, the local investigator will be involved to assess the potential causality between the IMP and the laboratory measurements. The local investigator will then decide if the participant needs to stop the intervention.
- Evidence of hyperlactatemia until day 7: In case of a clinical suspicion of hyperlactatemia, the local investigators must collect a blood sample to assess if the lactate level is above the upper norm according to specific laboratory-defined reference ranges. For increased lactate levels, the causality with the study treatment will be assessed. The local investigator will then decide if the participant needs to stop the intervention.
- Acute kidney injury: the kidney function is measured in the blood (creatinine) regularly as part of the clinical routine. To collect this outcome, the serum creatinine at baseline, on day 5 (±1), and only if participant remains hospitalised, at day 14 (±3 days) will be documented. The values will be captured from available data from the hospital information system.
- *C. difficile*-associated diarrhoea within 90 days: The treating clinicians will assess for *C. difficile* infection based on the centre-specific diagnostic algorithm if clinical suspicion arises due to diarrhoea during antibiotic treatment. *C. difficile* infection will be evaluated on day 14, at discharge, and on day 90 by reviewing the participant's electronic health records. If a participant reports severe diarrhoea during a 90-day follow-up phone call, occurring after discharge and managed outside the centre, the responsible physician may be contacted to determine if a *C. difficile* infection was diagnosed.

9.3.12 Assessment of serious adverse events and serious adverse reactions (from enrolment until discharge, and at day 90 ±7)

All assessments of serious adverse events (SAE) and serious adverse reactions (SAR) during acute care hospitalisation will be part of the routine clinical work-up and monitoring of the participants. This generally includes a daily routine visit and evaluation of the participant by the treating physician. Further, the vital signs (blood pressure, pulse, body temperature, oxygenation) will be measured regularly by the nursing staff. Blood samples will regularly be taken to measure organ function (myelopoiesis, liver, kidney). For severely ill participants treated on the intensive-care unit, the clinical as well as laboratory evaluation is generally much more frequent. Any serious adverse event will be reported to the study-site principal investigator, who then assesses whether there is a reasonable causal relationship with the IMP. Assessment will take place from enrolment until acute care hospital discharge. Further, any post-discharge occurrence of an SAE/SAR will be assessed during the phone call at day 90 follow-up.

9.3.13 Assessment of new antibiotic resistance (discharge day)

Any *S. aureus* detected at a new site of infection in a participant will routinely be tested for its antibiotic susceptibility as part of the clinical microbiological work-up and according to the respective standards of the responsible laboratory. In case of persistent or repetitive detection of *S. aureus* in the blood, the microbiological laboratory will decide if an antibiotic susceptibility testing should be repeated. Detection of any new resistance not present in the *S. aureus* from the initial blood culture will count as new antibiotic resistance. The standard susceptibility testing suffices; additional determination of the MIC of an antibiotic is not necessary except if clinically indicated. The data will be gathered from the hospital information system.

9.3.14 Assessment of microbiological failure (day 28, discharge day, day 90 ±7)

"Early microbiological failure" and "microbiological failure" are defined as the identification of any new focus of *S. aureus* between 5 and 13 days, or 14 and 90 days after randomisation, respectively. This will be assessed by reviewing the participant's microbiological results in the electronic health records. Any detection of *S. aureus* (by culture or by culture-independent methods such PCR) within the time frame will be further investigated in detail by reviewing the corresponding clinical health records. Thereby it can be determined – if necessary - whether the *S. aureus* originated from a sterile site. Any change of the initial antibiotic (and/or surgical) treatment plan will be analysed if it occurred due to proven detection of *S. aureus* at a sterile site. During the 90-day telephone call, it will be asked if any microbiological samples were taken outside of the corresponding centre. If this is the case, the responsible laboratory will be directly contacted to ask to provide the corresponding results. The principal investigator of each site will finally decide if a microbiological failure occurred. Furthermore, the independent outcome assessment board will review each documented microbiological failure on a case-by-case basis.

9.3.15 Assessment of clinical failure (day 14, day 28, discharge day, day 90)

"Early clinical failure" and "clinical failure" are defined as the identification of any new focus of *S. aureus* between 5 and 13 days, or 14 and 90 days after randomisation, respectively. This encompasses not only microbiological findings (i.e. detection of *S. aureus*) but also clinical, radiological and histological results. Clinical failure will be assessed by screening of patient history on multiple levels in the available electronic health records and during the telephone call at day 90: any change of the initial antibiotic (and/or surgical) treatment plan will be analysed if it occurred due to proven or assumed new focus of *S. aureus*. Further, all available microbiological results, histological results as well as radiological examens will be screened for signs of newly identified *S. aureus* focus/foci. In case of ambiguous interpretations, the treating physicians can be contacted directly to inquire his/her interpretation of the results. The principal investigator of each site will finally decide if a clinical failure occurred. Furthermore, a blinded panel of experts, selected by the trial investigators, will review each documented clinical failure on a case-by-case basis.

9.3.16 Assessment of length of hospital stay and length of ICU stay (discharge day)

At the time of acute care hospital discharge, the length of hospital stay will be documented according to the hospital records. Similarly, length of stay on the ICU will be extracted from the electronic hospital records.

9.3.17 Assessment of all-cause mortality (day 7, day 14, day 28, day 90 ±7)

The data will be examined in both the hospital records and, when available, external documents to ascertain whether the participant has died during the follow-up period. If the participant cannot be reached for the 90-day telephone follow-up, their emergency contact or primary care physician will be contacted. Documentation of the presumed cause of death is not mandatory.

9.3.18 Assessment of return to usual level of function (day 90 ±7)

During the phone call at day 90, the participant will be asked to rate their level of function in the 4 weeks prior to their bacteraemia and their current level of function from 6 to 1 according to the modified functional bloodstream infection score (see chapter 5.1 *Primary Outcome* for the corresponding scale). All levels of the scale will be presented orally in a clear and precise manner, ensuring that the participant has adequate time to self-assess. In the event of any misunderstanding regarding the classifications, the scale will be repeated as necessary. If the participant is unable to perform the self-assessment (whether due to medical reasons or limited communication abilities over the phone), efforts will be made to contact a family member or the participant's primary care physician to obtain the information.

9.3.19 Assessment of physical and mental health (quality of life, SF-36, day 90 ±7)

Quality of life (i.e. mental health and physical health) will be assessed at day 90 through the SF-36 questionnaire. Participants will have the option to complete it either electronically or on paper. At the time of enrolment in the study, participants will be asked which method they prefer. For the electronic version, participants will be requested to provide their email address and/or mobile phone number. This information will be encrypted and accessible only to the local Investigator of the respective centre. On day 90, participants will be notified via email or mobile phone

message about the task and will be directed to the corresponding online version of the study-specific SF-36 questionnaire. For participants who prefer the written version, a letter with a printed version of the SF-36 questionnaire will be sent by postal mail, including an envelope for returning the filled questionnaire. We expect that participants will need approximately 10 minutes to complete the questionnaire. At the time of the 90-day telephone call, participants will be asked whether they have already completed the questionnaire and if they have any questions regarding it. It is not intended for the questionnaire to be filled out verbally over the phone, as this could potentially introduce bias.

10. SAFETY

10.1 Drug studies

During the entire duration of the study, all serious adverse events (SAEs) are collected, fully investigated and documented in source documents and case report forms (CRF). Predefined adverse events of special interest (AE) critical to the safety evaluation will be assessed as outlined in the study protocol. Study duration encompasses the time from when the participant signs the informed consent form until the last protocol-specific procedure has been completed, including a safety follow-up period.

10.1.1 Definition and assessment of (serious) adverse events and other safety related events

An **Adverse Event (AE)** is any untoward medical occurrence in a patient or a clinical investigation participant administered a pharmaceutical product and which does not necessarily have a causal relationship with the study procedure. An AE can therefore be any unfavourable and unintended sign (including an abnormal laboratory finding), symptom, or disease temporally associated with the use of an investigational medicinal product, whether or not related to the investigational medicinal product. [ICH E6 1.2]

A **Serious Adverse Event (SAE)** is classified as any untoward medical occurrence that:

- results in death,
- is life-threatening,
- requires in-patient hospitalization or prolongation of existing hospitalisation,
- results in persistent or significant disability/incapacity, or
- is a congenital anomaly/birth defect.

In addition, important medical events that may not be immediately life-threatening or result in death, or require hospitalisation, but may jeopardise the patient or may require intervention to prevent one of the other outcomes listed above should also usually be considered serious. [ICH E2A]

SAEs should be followed until resolution or stabilisation. Participants with ongoing SAEs at study termination (including safety follow-up visit) will be further followed up until recovery or until stabilisation of the disease after termination.

Assessment of Causality

Both Investigator and Sponsor make a causality assessment of the event to the study investigational medicine product, based on the criteria listed in the ICH E2A guidelines:

Relationship	Description
Definitely	Temporal relationship<br>Improvement after de-challenge*<br>Recurrence after re-challenge<br>(or other proof of drug cause)
Probably	Temporal relationship<br>Improvement after de-challenge<br>No other cause evident
Possibly	Temporal relationship<br>Other cause possible
Unlikely	Any assessable reaction that does not fulfil the above conditions
Not related	Causal relationship can be ruled out

*Improvement after de-challenge only taken into consideration if applicable to reaction

Unexpected Adverse Drug Reaction

An “unexpected” adverse drug reaction is an adverse reaction, the nature or severity of which is not consistent with the applicable product information (e.g. Investigator’s Brochure for drugs that are not yet approved and Product Information for approved drugs, respectively). [ICH E2A]

Suspected Unexpected Serious Adverse Reactions (SUSARs)

The Sponsor evaluates the SAE that has been reported regarding seriousness, causality and expectedness. If the event is related to the investigational medicinal product and is both serious and unexpected, it is classified as a SUSAR.

Assessment of Severity

Severity of any AEs will be graded by the local Investigator as well as the Sponsor-Investigator based on the following criteria according to the Common Terminology Criteria for Adverse Events (CTCAE) Version 5.0 published November 27, 2017 [116].

Severity	Description
Grade 1	Mild; asymptomatic or mild symptoms; clinical or diagnostic observations only; intervention not indicated.
Grade 2	Moderate; minimal, local or non-invasive intervention indicated; limiting age-appropriate instrumental ADL*.
Grade 3	Severe or medically significant but not immediately life-threatening; hospitalization or prolongation of hospitalization indicated; disabling; limiting self care ADL**.
Grade 4	Life-threatening consequences; urgent intervention indicated.
Grade 5	Death related to AE.

A semi-colon indicates ‘or’ within the description of the grade. ADL: Activities of Daily Living

*Instrumental ADL refer to preparing meals, shopping for groceries or clothes, using the telephone, managing money, etc.

**Self care ADL refer to bathing, dressing and undressing, feeding self, using the toilet, taking medications, and not bedridden.

10.1.2 Reporting of serious adverse events (SAE) and other safety related events

Reporting of SAEs

All SAEs must be reported immediately and within a maximum of 24 hours of becoming aware of the SAE to the Sponsor-Investigator of the study. The Sponsor-Investigator will re-evaluate the SAE in a separate eCRF form and provide feedback to the site in case the Sponsor-Investigator’s opinion differs. In addition, SAEs will also be evaluated during the interim analyses with the DSMB (including external clinical experts as well as patient and public representatives; see also chapter *11.4.4 Interim analysis*).

Reporting of SUSARs

A SUSAR must be reported to the Ethics Committee (local event via local Investigator) via BASEC and to Swissmedic (via the Sponsor-Investigator) within 7 days of becoming aware of the SUSAR if the event is life-threatening or fatal. For all other SUSARs, the report must be made within 15 days.

The reporting obligations of SUSAR also apply if the investigator or the sponsor becomes aware of a suspected case after termination of the clinical trial.

Reporting of immediate safety and protective measures

All suspected new risks and relevant new aspects of known adverse reactions that require immediate safety-related measures, must be reported to the Sponsor-Investigator within 24 hours of becoming aware of the new risks. The Sponsor-Investigator must report these measures within 7 days to the Ethics Committee (local event via local Investigator) via BASEC and to Swissmedic.

The Sponsor must immediately inform all participating Investigators about the immediate safety and protective measures.

Reporting and Handling of Pregnancies

In the event of pregnancy, the treating physician and the participant (or the participant’s next of kin) will determine

whether the potential benefits for the participant outweigh the potential risks to the foetus. The course and outcome of the pregnancy should be followed up carefully, and any abnormal outcome regarding the mother or the child must be documented and reported.

Periodic reporting of safety and general progress of the clinical trial.

Once a year, the Sponsor-Investigator submits to the CEC and Swissmedic a list of the safety events including the severity of the events, their causality to the intervention and the safety of the study participants. This report also includes an update on the general progress of the study.

The safety and general progress reports encompass the time from the date of the sponsor's first authorisation to conduct the clinical trial in any country worldwide until the Last Patient Last Visit in Switzerland.

The safety and general progress report is submitted yearly to the CEC and to Swissmedic throughout the duration of the clinical trial in Switzerland, and the last submission of the safety report will cover the Last Patient Last Visit in Switzerland.

10.1.3 Follow up of (Serious) Adverse Events

SAEs occurring during hospitalisation will be followed-up until they are resolved during the hospital stay. If any study personnel become aware of an SAE outside of hospital, the responsible local investigator must conduct regular follow-ups until the SAE is resolved. At least 5 attempts at different times of the day to reach the participant will be conducted by the treating physician (or a delegated physician). If these fail, reasonable attempt will be made to acquire the information from relatives, caregivers, or the participant's emergency contact or primary care physician.

10.2 Assessment, notification and reporting on the use of radiation sources

Not applicable.

10.3 Exemption from the documentation requirements of AE

10.3.1 Exemption from the documentation requirements of AE due to pharmacological arguments

Not applicable.

10.3.2 Exemption from the documentation requirements of AE due to clinical arguments

Not applicable.

11. STATISTICAL METHODS

11.1 Hypothesis

Primary research hypothesis: Inhibition of virulence factor expression by early 5-day treatment with linezolid (600 mg twice daily) in addition to treatment with standard antibiotics will result in superior outcomes at 90 days compared to placebo in participants with *S. aureus* bacteraemia.

11.2 Determination of Sample Size

We collected retrospective data on the DOOR components from all *S. aureus* bacteraemia patients treated at the University Hospital Basel in 2022 (n=119). Our assumptions for the sample size are based on these data along with what is known from the literature to derive plausible estimates for the DOOR components. Patients who died in the first three days after a *S. aureus* bacteraemia diagnosis were excluded so that we do not overestimate the mortality of the planned trial (of note, most of these patients would probably have died before they could have been included in our study (n = 8)). Following extensive consultations with patient representatives, we have established that, for mortality at 90 days, a trial should demonstrate a meaningful relative difference of 10% (equating to an absolute difference of 2.2%). Meanwhile, for the remaining domains, the following relative reductions were deemed acceptable: 15% for level of function and 20% for complications and length of hospital stay).

The sample size was calculated using a simulation approach, for which 1000 synthetic datasets for each combination of plausible values for the relevant parameters were generated. We calculated the p-value of the win ratio for each of the simulated datasets and tested for statistical significance at $\alpha = 0.05$ using the unmatched win-ratio approach described in Pocock et al.[117]. The final sample size is based on the following specific assumptions:

- The statistical tests are conducted at a two-sided significance level of α=0.05, and the desired power is 90%
- The two treatment arms are equal in size (1:1 allocation)
- The proportion of deaths within 90 days in the control group is expected to be 22.5% and 20.3% in the linezolid group (relative reduction of 10%; absolute reduction of 2.2%)
- The proportion of participants with worse level of function in the control group is expected to be 25.0% [21], and 21.3% in the linezolid group (relative reduction of 15%; absolute reduction of 3.7%)
- The proportion of participants with any complications (defined DOOR component) will be 6.3% in the control group and 5.0% in the linezolid group (relative reduction of 20%; absolute reduction of 1.3%).
- We modelled hospital length of stay using a log-normal model with any of the complications listed in the definition above as an independent variable; the parameters for this model were derived from the retrospective USB data, and the expected 20% relative reduction in hospital length of stay due to the intervention was applied to the mean of the log-transformed data when generating synthetic datasets.
- Missing data were accounted for in the simulations (i.e., 10% for worse level of function and only 3% for all other components as these data will be available from routinely collected data). In case of missing DOOR components, the comparison among participants was made with the following DOOR domain.

Under the assumptions listed above, 550 participants are required to show a significant effect of the intervention. Since it is possible that some patients themselves or their next of kin withdraw consent post-randomisation, we increased the target sample size by 10% to minimise the risk of being underpowered. Hence, we plan to include 606 participants (303 participants in each study arm).

11.3 Statistical criteria of termination of trial

Not applicable. In brief, we do not have statistical criteria of early termination of the trial. Safety will be monitored by the independent DSMB (which includes patient representatives). If the DSMB observes a major safety concern (i.e. which would also be unacceptable if linezolid is superior to compared to standard of care) the DSMB will have the power to initiate the early termination of the trial.

11.4 Planned Analyses

11.4.1 Datasets to be analysed, analysis populations

Analyses will be performed on the full analysis set (i.e., all randomised participants) and the per protocol dataset

(i.e., those who received at least 7 out of 10 doses of linezolid or placebo). The primary estimand to be calculated in this study is the effect (summarised as the win ratio) of being randomly assigned to treatment with linezolid in the patient population (i.e., equivalent to an intention-to-treat analysis). We will also estimate the treatment effect among the treated participants (i.e., the per protocol dataset).

A detailed analysis plan will be written before closing the study database. Of note, we are currently conducting a systematic scoping review, assessing current practices how to analyse a hierarchical composite outcome. These results will inform our pre-specified analysis plan. Furthermore, we will consider further developments in risk-score classifications for *S. aureus* bacteraemia (e.g. for a potential use of a matched win-ratio versus unmatched win-ratio). The statistical analysis plan will be made publicly available (on clinicaltrials.gov). Currently we intend to conduct the following analyses:

11.4.2 Primary Analysis

Treatment and placebo will be compared on the basis of the win-ratio approach as described by Pocock and colleagues:[117] In brief, each participant in the treatment group will be compared with each participant in the placebo group. When comparing two participants, the winner will be determined by the first component of the DOOR in which the two participants differ. The following two scenarios will be considered as a tie: (i) when both participants died within 90 days, or (ii) when participants have the same outcomes for all DOOR elements, including length of hospital stay. The win ratio will be calculated by dividing the number of cases in which participants receiving linezolid win compared with the number of cases in which participants receiving placebo win. We will reject the null hypothesis that the win ratio is equal to 1 if the *p*-value is less than 0.05.

Subgroup analyses: For the primary outcome and for all DOOR components separately, we will assess if treatment effects differ by sex (male vs. female), ICU status at baseline (participants on ICU vs. participants not on ICU) and focus of infection (vascular catheter, skin and soft tissue, endocarditis, osteoarticular, pneumonia, other focus, or focus not identified). All subgroup analyses will be exploratory in nature.

11.4.3 Secondary Analyses

We intend to conduct the following analyses for the secondary outcomes:

Logistic regression

- Returning to usual level of function by day 90
- Microbiological treatment failure 14-90 days after randomisation
- Early microbiological treatment failure 5-13 days after randomisation
- Clinical treatment failure 14-90 days after randomisation
- Persistent bacteraemia defined as positive blood culture on day 5 after randomisation
- Meeting two or more SIRS criteria simultaneously on day 5 after randomisation

Log-normal linear regression

- Length of hospital stay of acute index inpatient hospitalisation (in days)

Poisson regression

- Number of days without being on the Intensive Care Unit (ICU) up to day 90
- Number of days alive and free of antibiotics up to day 90

Cox proportional hazards model

- Time to death up to day 90

Fine & Gray competing risk model

- Time to being discharged alive (assessed until day 90)

The models listed above will include sex, ICU stay at baseline, and centre as adjustment factors (will be further pre-specified in separate data analysis plan).

Wilcoxon-Mann-Whitney test

- Physical health at day 90 as assessed by the short form 36 (SF-36) questionnaire
- Mental health at day 90 as assessed by the short form 36 (SF-36) questionnaire

Subgroup analyses: We will conduct the following subgroup analyses on the primary outcome to assess if treatment effects differ by sex (male vs. female), ICU status at baseline (participants on ICU vs. participants not on ICU), and dominant focus of infection (vascular catheter, skin and soft tissue, endocarditis, osteoarticular, pneumonia, other focus, or focus not identified).

11.4.4 Interim analyses

After the treatment of 50 participants and again after 200 participants, the independent Data and Safety Monitoring Board (DSMB, including external clinical experts as well as patient and public representatives) will meet to discuss summary statistics about safety outcomes (see section 10 *SAFETY*). In addition, the adherence to treatment will be assessed in these interim analyses. If the proportion of non-adherence is above 10% (i.e., more than 10% of participants received fewer than 7 of the planned 10 doses), the study team will assess the reasons why doses were missed and implement actions (e.g., additional training) to increase adherence.

11.4.5 Safety analysis

The following adverse events and safety outcomes will be analysed descriptively by study group:

- Serious adverse reactions or serious adverse events in the 90 days following study entry
- The following adverse events of special interest until 14 days after study entry during the index hospital stay:
  - Clinical signs compatible with serotonin syndrome
  - Laboratory signs of haematotoxicity
- Acute kidney injury defined using modified Kidney Disease Improving Global Outcomes (KDIGO) criteria, modified according to SNAP trial:
  - Increase in serum creatinine by 26.5 µmol/l or more at any time from baseline entry to day 5

    OR

  - Increase in serum creatinine by 1.5 times or more the level at study entry (baseline) within 14 days of study entry
- *Clostridioides difficile*-associated diarrhoea

11.4.6 Deviation(s) from the original statistical plan

If substantial deviations of the analysis as outlined in these chapters are needed for whatever reason, the protocol will be amended. All deviations of the analysis from the protocol or from the detailed analysis plan will be listed and justified in a separate chapter of the final statistical report.

11.5 Handling of missing data and drop-outs

The primary analysis will be performed on the data available (complete cases). In the sample size calculation, we have included expected proportions of missing data for the individual DOOR components. To keep as many participants as possible in the analysis, we will use the components of the DOOR that are not missing in cases where not all components are missing. For the secondary outcomes, we will consider multiple imputation if the proportion of excluded participants is larger than 5% (the imputation strategy will be pre-specified for each outcome in a separate data analysis plan).

12. QUALITY ASSURANCE AND CONTROL

Written standard operating procedures (SOPs) and manuals of operation will be issued to all sites and adherence to guidance is monitored during site visits.

12.1 Data handling and record keeping / archiving

12.1.1 Electronic Case Report Forms (eCRF)

Relevant clinical study data for each enrolled study participant, i.e., observations, tests and assessments specified in the protocol, are recorded in eCRFs via the web-based electronic data capture (EDC) system implemented in REDCap® at the study centres. The participant's name and address will not be recorded. The EDC system includes guidance for study sites on how to perform data entry and will also be used for query handling.

Local investigators and site trial members will be authorized for the eCRF entries of study participants enrolled at the site. Investigators will be trained to use the EDC system during the site initiation visit. The investigators ensure the accuracy, completeness, and timeliness of the data recorded and provide answers to data queries, as specified in the study protocol and in accordance with additional instructions. The identity of the local investigator entering data, and date and time of data entry will be recorded as meta-data in the study database.

12.1.2 Specification of source documents

Source data will include all study documents (e.g. informed consent forms, online AE/SAE forms, patient data in hospital information system). Source data will be available at all sites and may be found in paper or electronic form.

12.1.3 Record keeping / archiving

The sponsor retains all data relating to the clinical trial for at least twenty years after completion or premature termination of the clinical trial. The local investigators retain all documents necessary for the identification and follow-up of the trial participants and all other original data for at least twenty years after completion or discontinuation of the clinical trial. Data will be stored in the REDCap study database located on servers of the University of Basel. Any study relevant paper documents will be archived at each site for a minimum of 20 years.

12.2 Data management

12.2.1 Data Management System

Study data will be captured via the web-based electronic data capture system REDCap®. The system is developed by Vanderbilt University, US and licensed and hosted by the Department of Clinical Research of the University Basel. The REDCap® software installation and periodical updates are validated by the Department of Clinical Research. All data is stored on servers of the University Hospital Basel in Switzerland. Password protection and user-right management safeguard access, ensuring that only authorized personnel can view or modify the data.

Patients will be entered into REDCap® with a unique study ID. Patient data entry in REDCap® is facilitated by manually entering the unique hospital identification number, which is used to locate and input data at predefined time points. Using data-access groups in REDCap® will ensure that each local investigator (or delegated personnel) can only access data from their respective centre. To enhance data security and confidentiality, the unique hospital identification number is designated as an identifier field in REDCap®, preventing its inclusion in exported datasets. In these exports only the unique study ID of each participant will be included. To ensure that patients can still be correctly identified, each centre will maintain a separate key list that also serves as a site screening and enrolment log file. All data collected is entered into study specific case report forms. An audit trail maintains a record of initial entries and any changes made; time and date of entry; and username of person authorizing entry or change.

12.2.2 Data security, access, and back-up

Data is encrypted at rest and protected by advanced threat detection software according to the security policy of the University Hospital Basel. REDCap® is accessible via a standard browser on devices with internet connection. The data transfer between clients and servers is encrypted using Transport Layer Encryption (TLS) cryptography protocol. Password protection and user-right management ensure that only authorized study investigators, monitors, data managers and local authorities (if necessary) will have access to the data during and after the study.

User administration and user training is performed by the Department of Clinical Research (DKF) Basel according to predefined processes. The built-in audit trail will register any unplanned deviations from the study protocol.

Backup of the REDCap® database is performed daily according to established processes.

12.2.3 Analysis and archiving

REDCap database will be locked after all data has been monitored and cleaned, and all raised queries have been resolved. Data will be archived by the Sponsor for a minimum of 20 years.

12.2.4 Electronic and central data validation

The data managers of the DKF Basel will implement validation rules in REDCap®. When data gets saved in a case report form, it will be validated for discrepancies. The data will be reviewed by the responsible investigator as well as an independent monitor. The monitor will raise queries using the query management system implemented in REDCap®. Designated investigators must respond to the query and confirm or correct the corresponding data. Thereafter the monitor can close the query.

12.3 Monitoring

The Department of Clinical Research of the University of Basel will perform the monitoring of this trial. A monitor from the Department of Clinical Research will, in accordance with the Sponsor, contact all sites for initiation of the trial. According to the scope of the monitoring plan, the monitor will visit the trial site during the study or perform remote monitoring visits, regularly if necessary, and after closing. The monitor will verify the adherence to the protocol, completeness, consistence, and accuracy of the data being entered on eCRF and will thus require access to all patient medical records including laboratory test results and surgery, pathology and radiology reports and supporting documents. The investigator (or his/her delegate) should collaborate with the monitor. They must ensure that source data verification forms, source data and documents are made accessible to the study monitor, and they should answer questions by the Study Monitor in detail to ensure that any problems detected during these visits are resolved. Open action items will be listed and explained by the monitor in follow-up letters and are expected to be resolved in reasonable time.

12.4 Audits and Inspections

In accordance with ICH GCP guidelines [2], audits may be performed by the CEC and CA during the course of the study. The audits will include control of adherence to the protocol, standard operating procedures, ICU GSP guidelines and national legislation. Source data verification and checking of the data entered in the eCRF will be used for assessment of complete and reliable documentation. The local investigators ensure that source data and documents are made accessible to auditors and inspectors and answer their questions. All involved parties must keep the participant data strictly confidential.

12.5 Confidentiality, Data Protection

Direct access to source documents will be permitted for purposes of monitoring, audits and inspections.

Study data entered into the eCRF is only accessible to authorized personnel. Once all data is entered into the REDCap platform and monitoring is completed, the database will be locked and closed for further data entry. The complete dataset is then exported and transferred to the study statistician as well as the principal investigator through a secure channel.

12.6 Storage of biological material and related health data

Plasma samples used for linezolid trough measurement will be coded and stored until the analyses are performed. Samples will be stored locally at the participating centre until shipment to the sponsor site (University Hospital Basel). Any leftover samples will be stored in the LIPS biobank. Detailed information can be found in the LIPS biobanking regulation.

13. PUBLICATION AND DISSEMINATION POLICY

The trial results will be published in a peer-reviewed open access journal. Results will also be published if they are negative or if the trial was discontinued. The full protocol will be made publicly available on clinicaltrials.gov. A detailed analysis plan will be written before closing the study database. The statistical analysis plan will be made publicly available on clinicaltrials.gov. Furthermore, we will consider publishing a short peer-reviewed version of the trial protocol. Authorship to publications will be granted according to the rules of the International Committee of Medical Journal Editors (ICMJE). After publication of the main results, the full dataset will be submitted to the Data Access Committee of the Faculty of Medicine (MF-DAC) at the University of Basel. Other researchers interested in re-using the study data may then contact the independent Data Access Committee from the University Hospital of Basel.

The sponsor will enter and publish a summary of the trial results in a public register in accordance with ClinO Art. 65a within one year of completion or premature termination of the trial. An interruption lasting more than two years is considered a premature termination of the trial.

For the purpose of publication in the public register the sponsor also ensures that a lay summary of the trial results in German, French, and Italian is entered in BASEC within one year of completion or premature termination of the trial.

The investigator will provide each study participants with the lay summary of the trial results at the end of the study, directly and inform them where they can find the study results.

14. FUNDING AND SUPPORT

14.1 Funding

The LIPS trial is funded by the Swiss National Science Foundation (SNSF) under the Investigator initiated clinical trials (IICT) funding call (SNSF ID: 221668).

15. INSURANCE

Insurance with a policy value in accordance with ClinO Annex 2 will be provided by the Sponsor University Hospital Basel. A copy of the certificate is filed in each investigator site file and the trial master file. The certificate will be provided as soon as ethics approval has been obtained.

16. TRIAL SUB-PROJECT(S)

16.1 Molecular mechanisms of *S. aureus* bacteraemia

Hypothesis: *S. aureus* uses intracellular survival inside immune cells as a main survival strategy in the blood for persistence of infection.

Objective: Identification of human cells harbouring *S. aureus* in bacteraemia.

Background: Despite extensive research, the mechanisms underlying *S. aureus* persistence in the bloodstream for more than 48 hours despite active antibiotic therapy remain unclear[8 9]. The associated stark increase of mortality and risk of metastatic spread[18] in these courses of infection underline the importance of further research. Current investigations are limited to *in vitro* and animal studies, lacking sufficient predictive power for the relevant patient setting. Based on these previous findings, *S. aureus* can survive and proliferate within human neutrophils and monocytes[118] and could, as hypothesized[119], use the intracellular niche as a crucial way of evading antibiotic exposure. The existence and relevance of intracellular *S. aureus in* bacteraemia in humans has not yet been shown.

Methods: Blood samples will be drawn daily in routine or study-specific blood draws and sent to the research facility as described in 9.3.7 until 48h after the patient's first negative blood culture. Samples will be divided to perform several analyses: i) Quantification of intracellular and extracellular viable bacteria will be achieved by plating of serum respectively plating of lysed cells on agar plates for counting of colony forming units ii) blood smears stained with fluorescent vancomycin derivate VAN-JF669[120] for intracellular bacterial staining and other standard stainings (e.g. RAB5, RAB7, fibrinogen) will be analysed on a Nikon Ti2 confocal microscope iii) Flow cytometry analysis will be performed on a subsection of blood cells. Using Percoll separation, specific blood cell types are obtained and stained with VAN-JF669 and for cell type markers (e.g. CD14, CD16, CD68). Cells will be analysed using spectral flow cytometry (Cytek Aurora) or image flow cytometry (Amnis ImageStreamX Mark II).

17. REFERENCES

1. Collaborators GBDAR. Global mortality associated with 33 bacterial pathogens in 2019: a systematic analysis for the Global Burden of Disease Study 2019. *Lancet* 2022;400(10369):2221-48. doi: 10.1016/S0140-6736(22)02185-7 [published Online First: 20221121]
2. Renggli L, Gasser M, Buetti N, et al. Increase in methicillin-susceptible Staphylococcus aureus bloodstream infections in Switzerland: a nationwide surveillance study (2008-2021). *Infection* 2023;51(4):1025-31. doi: 10.1007/s15010-023-01980-6 [published Online First: 20230202]
3. Laupland KB, Lyytikainen O, Sogaard M, et al. The changing epidemiology of Staphylococcus aureus bloodstream infection: a multinational population-based surveillance study. *Clin Microbiol Infect* 2013;19(5):465-71. doi: 10.1111/j.1469-0691.2012.03903.x [published Online First: 20120523]
4. Hindy JR, Quintero-Martinez JA, Lee AT, et al. Incidence Trends and Epidemiology of Staphylococcus aureus Bacteremia: A Systematic Review of Population-Based Studies. *Cureus* 2022;14(5):e25460. doi: 10.7759/cureus.25460 [published Online First: 20220529]
5. Benfield T, Espersen F, Frimodt-Moller N, et al. Increasing incidence but decreasing in-hospital mortality of adult Staphylococcus aureus bacteraemia between 1981 and 2000. *Clin Microbiol Infect* 2007;13(3):257-63. doi: 10.1111/j.1469-0691.2006.01589.x
6. Mathe P, Gopel S, Hornuss D, et al. Increasing numbers and complexity of Staphylococcus aureus bloodstream infection-14 years of prospective evaluation at a German tertiary care centre with multi-centre validation of findings. *Clin Microbiol Infect* 2023;29(9):1197 e9-97 e15. doi: 10.1016/j.cmi.2023.05.031 [published Online First: 20230603]
7. Bai AD, Lo CK, Komorowski AS, et al. Staphylococcus aureus bacteremia mortality across country income groups: A secondary analysis of a systematic review. *Int J Infect Dis* 2022;122:405-11. doi: 10.1016/j.ijid.2022.06.026 [published Online First: 20220618]
8. Kaasch AJ, Barlow G, Edgeworth JD, et al. Staphylococcus aureus bloodstream infection: a pooled analysis of five prospective, observational studies. *J Infect* 2014;68(3):242-51. doi: 10.1016/j.jinf.2013.10.015 [published Online First: 20131116]
9. Bai AD, Lo CKL, Komorowski AS, et al. Staphylococcus aureus bacteraemia mortality: a systematic review and meta-analysis. *Clin Microbiol Infect* 2022;28(8):1076-84. doi: 10.1016/j.cmi.2022.03.015 [published Online First: 20220323]
10. Wiggli BJ, Frei R, Laffer R, et al. Survival from methicillin-sensitive Staphylococcus aureus bloodstream infections over 20 years: a cohort of 1328 patients. *Swiss Med Wkly* 2017;147:w14508. doi: 10.4414/smw.2017.14508 [published Online First: 20170930]
11. Smit J, Lopez-Cortes LE, Kaasch AJ, et al. Gender differences in the outcome of community-acquired Staphylococcus aureus bacteraemia: a historical population-based cohort study. *Clin Microbiol Infect* 2017;23(1):27-32. doi: 10.1016/j.cmi.2016.06.002 [published Online First: 20160622]
12. Nambiar K, Seifert H, Rieg S, et al. Survival following Staphylococcus aureus bloodstream infection: A prospective multinational cohort study assessing the impact of place of care. *J Infect* 2018;77(6):516-25. doi: 10.1016/j.jinf.2018.08.015 [published Online First: 20180901]
13. Westgeest AC, Ruffin F, Kair JL, et al. The association of female sex with management and mortality in patients with Staphylococcus aureus bacteraemia. *Clin Microbiol Infect* 2023;29(9):1182-87. doi: 10.1016/j.cmi.2023.06.009 [published Online First: 20230614]
14. Yahav D, Yassin S, Shaked H, et al. Risk factors for long-term mortality of Staphylococcus aureus bacteremia. *Eur J Clin Microbiol Infect Dis* 2016;35(5):785-90. doi: 10.1007/s10096-016-2598-8 [published Online First: 20160212]
15. Mansur N, Hazzan R, Paul M, et al. Does sex affect 30-day mortality in Staphylococcus aureus bacteremia? *Gend Med* 2012;9(6):463-70. doi: 10.1016/j.genm.2012.10.009 [published Online First: 20121107]
16. Humphreys H, Fitzpatick F, Harvey BJ. Gender differences in rates of carriage and bloodstream infection caused by methicillin-resistant Staphylococcus aureus: are they real, do they matter and why? *Clin Infect Dis* 2015;61(11):1708-14. doi: 10.1093/cid/civ576 [published Online First: 20150722]
17. Holland TL, Cosgrove SE, Doernberg SB, et al. Ceftobiprole for Treatment of Complicated Staphylococcus aureus Bacteremia. *N Engl J Med* 2023 doi: 10.1056/NEJMoa2300220 [published Online First: 20230927]
18. Kuehl R, Morata L, Boeing C, et al. Defining persistent Staphylococcus aureus bacteraemia: secondary analysis of a prospective cohort study. *Lancet Infect Dis* 2020;20(12):1409-17. doi: 10.1016/S1473-3099(20)30447-3 [published Online First: 20200804]
19. Souli M, Ruffin F, Choi SH, et al. Changing Characteristics of Staphylococcus aureus Bacteremia: Results From a 21-Year, Prospective, Longitudinal Study. *Clin Infect Dis* 2019;69(11):1868-77. doi: 10.1093/cid/ciz112
20. Eichenberger EM, de Vries CR, Ruffin F, et al. Microbial Cell-Free DNA Identifies Etiology of Bloodstream Infections, Persists Longer Than Conventional Blood Cultures, and Its Duration of Detection Is Associated With Metastatic Infection in Patients With Staphylococcus aureus and Gram-Negative Bacteremia. *Clin*

Infect Dis 2022;74(11):2020-27. doi: 10.1093/cid/ciab742

21. Jacobsson G, Gustafsson E, Andersson R. Outcome for invasive Staphylococcus aureus infections. *Eur J Clin Microbiol Infect Dis* 2008;27(9):839-48. doi: 10.1007/s10096-008-0515-5 [published Online First: 20080501]
22. Vogel M, Schmitz RP, Hagel S, et al. Infectious disease consultation for Staphylococcus aureus bacteremia - A systematic review and meta-analysis. *J Infect* 2016;72(1):19-28. doi: 10.1016/j.jinf.2015.09.037 [published Online First: 20151009]
23. Thorlacius-Ussing L, Sandholdt H, Nissen J, et al. Comparable Outcomes of Short-Course and Prolonged-Course Therapy in Selected Cases of Methicillin-Susceptible Staphylococcus aureus Bacteremia: A Pooled Cohort Study. *Clin Infect Dis* 2021;73(5):866-72. doi: 10.1093/cid/ciab201
24. Tong SYC, Lye DC, Yahav D, et al. Effect of Vancomycin or Daptomycin With vs Without an Antistaphylococcal beta-Lactam on Mortality, Bacteremia, Relapse, or Treatment Failure in Patients With MRSA Bacteremia: A Randomized Clinical Trial. *JAMA* 2020;323(6):527-37. doi: 10.1001/jama.2020.0103
25. Tong SYC, Mora J, Bowen AC, et al. The Staphylococcus aureus Network Adaptive Platform Trial Protocol: New Tools for an Old Foe. *Clin Infect Dis* 2022;75(11):2027-34. doi: 10.1093/cid/ciac476
26. Holland TL, Arnold C, Fowler VG, Jr. Clinical management of Staphylococcus aureus bacteremia: a review. *JAMA* 2014;312(13):1330-41. doi: 10.1001/jama.2014.9743
27. Tong SY, Davis JS, Eichenberger E, et al. Staphylococcus aureus infections: epidemiology, pathophysiology, clinical manifestations, and management. *Clin Microbiol Rev* 2015;28(3):603-61. doi: 10.1128/CMR.00134-14
28. Hagel SK, A.; Weis, S.; Seifert, H.; Pletz, M.; Rieg, S. Staphylococcus-aureus-Blutstrominfektion – eine interdisziplinäre Herausforderung. *Anästhesiol Intensivmed Notfallmed Schmerzther* 2019;54:206-16.
29. Kimmig A, Hagel S, Weis S, et al. Management of Staphylococcus aureus Bloodstream Infections. *Front Med (Lausanne)* 2020;7:616524. doi: 10.3389/fmed.2020.616524 [published Online First: 20210305]
30. Grillo S, Puig-Asensio M, Schweizer ML, et al. The Effectiveness of Combination Therapy for Treating Methicillin-Susceptible Staphylococcus aureus Bacteremia: A Systematic Literature Review and a Meta-Analysis. *Microorganisms* 2022;10(5) doi: 10.3390/microorganisms10050848 [published Online First: 20220420]
31. Abrams B, Sklaver A, Hoffman T, et al. Single or combination therapy of staphylococcal endocarditis in intravenous drug abusers. *Ann Intern Med* 1979;90(5):789-91. doi: 10.7326/0003-4819-90-5-789
32. Korzeniowski O, Sande MA. Combination antimicrobial therapy for Staphylococcus aureus endocarditis in patients addicted to parenteral drugs and in nonaddicts: A prospective study. *Ann Intern Med* 1982;97(4):496-503. doi: 10.7326/0003-4819-97-4-496
33. Ribera E, Gomez-Jimenez J, Cortes E, et al. Effectiveness of cloxacillin with and without gentamicin in short-term therapy for right-sided Staphylococcus aureus endocarditis. A randomized, controlled trial. *Ann Intern Med* 1996;125(12):969-74. doi: 10.7326/0003-4819-125-12-199612150-00005
34. Van der Auwera P, Klastersky J, Thys JP, et al. Double-blind, placebo-controlled study of oxacillin combined with rifampin in the treatment of staphylococcal infections. *Antimicrob Agents Chemother* 1985;28(4):467-72. doi: 10.1128/AAC.28.4.467
35. Thwaites GE, Scarborough M, Szubert A, et al. Adjunctive rifampicin for Staphylococcus aureus bacteraemia (ARREST): a multicentre, randomised, double-blind, placebo-controlled trial. *Lancet* 2018;391(10121):668-78. doi: 10.1016/S0140-6736(17)32456-X [published Online First: 20171214]
36. Ruotsalainen E, Jarvinen A, Koivula I, et al. Levofloxacin does not decrease mortality in Staphylococcus aureus bacteraemia when added to the standard treatment: a prospective and randomized clinical trial of 381 patients. *J Intern Med* 2006;259(2):179-90. doi: 10.1111/j.1365-2796.2005.01598.x
37. Cheng MP, Lawandi A, Butler-Laporte G, et al. Adjunctive Daptomycin in the Treatment of Methicillin-susceptible Staphylococcus aureus Bacteremia: A Randomized, Controlled Trial. *Clin Infect Dis* 2021;72(9):e196-e203. doi: 10.1093/cid/ciaa1000
38. Grillo S, Pujol M, Miro JM, et al. Cloxacillin plus fosfomycin versus cloxacillin alone for methicillin-susceptible Staphylococcus aureus bacteremia: a randomized trial. *Nat Med* 2023 doi: 10.1038/s41591-023-02569-0 [published Online First: 20231002]
39. Kwiecinski JM, Horswill AR. Staphylococcus aureus bloodstream infections: pathogenesis and regulatory mechanisms. *Curr Opin Microbiol* 2020;53:51-60. doi: 10.1016/j.mib.2020.02.005 [published Online First: 20200312]
40. Howden BP, Giulieri SG, Wong Fok Lung T, et al. Staphylococcus aureus host interactions and adaptation. *Nat Rev Microbiol* 2023;21(6):380-95. doi: 10.1038/s41579-023-00852-y [published Online First: 20230127]
41. Ahmad-Mansour N, Loubet P, Pouget C, et al. Staphylococcus aureus Toxins: An Update on Their Pathogenic Properties and Potential Treatments. *Toxins (Basel)* 2021;13(10) doi: 10.3390/toxins13100677 [published Online First: 20210923]
42. Liu Y, Gao W, Yang J, et al. Contribution of Coagulase and Its Regulator SaeRS to Lethality of CA-MRSA 923

Bacteremia. *Pathogens* 2021;10(11) doi: 10.3390/pathogens10111396 [published Online First: 20211028]

43. van der Vaart TW, Prins JM, Soetekouw R, et al. All-Cause and Infection-Related Mortality in Staphylococcus aureus Bacteremia, a Multicenter Prospective Cohort Study. *Open Forum Infect Dis* 2022;9(12):ofac653. doi: 10.1093/ofid/ofac653 [published Online First: 20221130]
44. Quartin AA, Schein RM, Kett DH, et al. Magnitude and duration of the effect of sepsis on survival. Department of Veterans Affairs Systemic Sepsis Cooperative Studies Group. *JAMA* 1997;277(13):1058-63.
45. Prescott HC, Iwashyna TJ, Blackwood B, et al. Understanding and Enhancing Sepsis Survivorship. Priorities for Research and Practice. *Am J Respir Crit Care Med* 2019;200(8):972-81. doi: 10.1164/rccm.201812-2383CP
46. King HA, Doernberg SB, Miller J, et al. Patients' Experiences With Staphylococcus aureus and Gram-negative Bacterial Bloodstream Infections: A Qualitative Descriptive Study and Concept Elicitation Phase To Inform Measurement of Patient-reported Quality of Life. *Clin Infect Dis* 2021;73(2):237-47. doi: 10.1093/cid/ciaa611
47. Hodille E, Rose W, Diep BA, et al. The Role of Antibiotics in Modulating Virulence in Staphylococcus aureus. *Clin Microbiol Rev* 2017;30(4):887-917. doi: 10.1128/CMR.00120-16
48. Dotel R, Tong SYC, Bowen A, et al. CASSETTE-clindamycin adjunctive therapy for severe Staphylococcus aureus treatment evaluation: study protocol for a randomised controlled trial. *Trials* 2019;20(1):353. doi: 10.1186/s13063-019-3452-y [published Online First: 20190613]
49. Stevens DL, Bisno AL, Chambers HF, et al. Practice guidelines for the diagnosis and management of skin and soft tissue infections: 2014 update by the infectious diseases society of America. *Clin Infect Dis* 2014;59(2):147-59. doi: 10.1093/cid/ciu296 [published Online First: 20140618]
50. Liu C, Bayer A, Cosgrove SE, et al. Clinical practice guidelines by the infectious diseases society of america for the treatment of methicillin-resistant Staphylococcus aureus infections in adults and children: executive summary. *Clin Infect Dis* 2011;52(3):285-92. doi: 10.1093/cid/cir034
51. Gillet Y, Dumitrescu O, Tristan A, et al. Pragmatic management of Panton-Valentine leukocidin-associated staphylococcal diseases. *Int J Antimicrob Agents* 2011;38(6):457-64. doi: 10.1016/j.ijantimicag.2011.05.003 [published Online First: 20110705]
52. Brown NM, Goodman AL, Horner C, et al. Treatment of methicillin-resistant Staphylococcus aureus (MRSA): updated guidelines from the UK. *JAC Antimicrob Resist* 2021;3(1):dlaa114. doi: 10.1093/jacamr/dlaa114 [published Online First: 20210203]
53. STADAPHARM. Linezolid STADA® 600 mg SPC.
54. Chiang FY, Climo M. Efficacy of linezolid alone or in combination with vancomycin for treatment of experimental endocarditis due to methicillin-resistant Staphylococcus aureus. *Antimicrob Agents Chemother* 2003;47(9):3002-4. doi: 10.1128/AAC.47.9.3002-3004.2003
55. Jacqueline C, Navas D, Batard E, et al. In vitro and in vivo synergistic activities of linezolid combined with subinhibitory concentrations of imipenem against methicillin-resistant Staphylococcus aureus. *Antimicrob Agents Chemother* 2005;49(1):45-51. doi: 10.1128/AAC.49.1.45-51.2005
56. Pachon-Ibanez ME, Ribes S, Dominguez MA, et al. Efficacy of fosfomycin and its combination with linezolid, vancomycin and imipenem in an experimental peritonitis model caused by a Staphylococcus aureus strain with reduced susceptibility to vancomycin. *Eur J Clin Microbiol Infect Dis* 2011;30(1):89-95. doi: 10.1007/s10096-010-1058-0 [published Online First: 20100916]
57. Diep BA, Afasizheva A, Le HN, et al. Effects of linezolid on suppressing in vivo production of staphylococcal toxins and improving survival outcomes in a rabbit model of methicillin-resistant Staphylococcus aureus necrotizing pneumonia. *J Infect Dis* 2013;208(1):75-82. doi: 10.1093/infdis/jit129 [published Online First: 20130326]
58. Yanagihara K, Kihara R, Araki N, et al. Efficacy of linezolid against Panton-Valentine leukocidin (PVL)-positive meticillin-resistant Staphylococcus aureus (MRSA) in a mouse model of haematogenous pulmonary infection. *Int J Antimicrob Agents* 2009;34(5):477-81. doi: 10.1016/j.ijantimicag.2009.06.024 [published Online First: 20090807]
59. Wunderink RG, Rello J, Cammarata SK, et al. Linezolid vs vancomycin: analysis of two double-blind studies of patients with methicillin-resistant Staphylococcus aureus nosocomial pneumonia. *Chest* 2003;124(5):1789-97.
60. Zahedi Bialvaei A, Rahbar M, Yousefi M, et al. Linezolid: a promising option in the treatment of Gram-positives. *J Antimicrob Chemother* 2017;72(2):354-64. doi: 10.1093/jac/dkw450 [published Online First: 20161220]
61. Dagher M, Fowler VG, Jr., Wright PW, et al. A Narrative Review of Early Oral Stepdown Therapy for the Treatment of Uncomplicated Staphylococcus aureus Bacteremia: Yay or Nay? *Open Forum Infect Dis* 2020;7(6):ofaa151. doi: 10.1093/ofid/ofaa151 [published Online First: 20200505]
62. Shorr AF, Kunkel MJ, Kollef M. Linezolid versus vancomycin for Staphylococcus aureus bacteraemia: pooled analysis of randomized studies. *J Antimicrob Chemother* 2005;56(5):923-9. doi: 10.1093/jac/dki355 [published Online First: 20050929]

63. Falagas ME, Siempos, II, Vardakas KZ. Linezolid versus glycopeptide or beta-lactam for treatment of Gram-positive bacterial infections: meta-analysis of randomised controlled trials. *Lancet Infect Dis* 2008;8(1):53-66. doi: 10.1016/S1473-3099(07)70312-2

64. Park HJ, Kim SH, Kim MJ, et al. Efficacy of linezolid-based salvage therapy compared with glycopeptide-based therapy in patients with persistent methicillin-resistant Staphylococcus aureus bacteremia. *J Infect* 2012;65(6):505-12. doi: 10.1016/j.jinf.2012.08.007 [published Online First: 20120814]

65. Jang HC, Kim SH, Kim KH, et al. Salvage treatment for persistent methicillin-resistant Staphylococcus aureus bacteremia: efficacy of linezolid with or without carbapenem. *Clin Infect Dis* 2009;49(3):395-401. doi: 10.1086/600295

66. Kelesidis T, Humphries R, Ward K, et al. Combination therapy with daptomycin, linezolid, and rifampin as treatment option for MRSA meningitis and bacteremia. *Diagn Microbiol Infect Dis* 2011;71(3):286-90. doi: 10.1016/j.diagmicrobio.2011.07.001 [published Online First: 20110819]

67. Wilcox MH, Tack KJ, Bouza E, et al. Complicated skin and skin-structure infections and catheter-related bloodstream infections: noninferiority of linezolid in a phase 3 study. *Clin Infect Dis* 2009;48(2):203-12. doi: 10.1086/595686

68. Gomez J, Garcia-Vazquez E, Banos R, et al. Predictors of mortality in patients with methicillin-resistant Staphylococcus aureus (MRSA) bacteraemia: the role of empiric antibiotic therapy. *Eur J Clin Microbiol Infect Dis* 2007;26(4):239-45. doi: 10.1007/s10096-007-0272-x

69. Stevens DL, Wallace RJ, Hamilton SM, et al. Successful treatment of staphylococcal toxic shock syndrome with linezolid: a case report and in vitro evaluation of the production of toxic shock syndrome toxin type 1 in the presence of antibiotics. *Clin Infect Dis* 2006;42(5):729-30. doi: 10.1086/500265

70. Li HT, Zhang TT, Huang J, et al. Factors associated with the outcome of life-threatening necrotizing pneumonia due to community-acquired Staphylococcus aureus in adult and adolescent patients. *Respiration* 2011;81(6):448-60. doi: 10.1159/000319557 [published Online First: 20101106]

71. Pfizer AG Zürich. Zyvoxid Fachinformation Schweiz, 2023.

72. Bishop E, Melvani S, Howden BP, et al. Good clinical outcomes but high rates of adverse reactions during linezolid therapy for serious infections: a proposed protocol for monitoring therapy in complex patients. *Antimicrob Agents Chemother* 2006;50(4):1599-602. doi: 10.1128/AAC.50.4.1599-1602.2006

73. Veerman K, Goosen J, Spijkers K, et al. Prolonged use of linezolid in bone and joint infections: a retrospective analysis of adverse effects. *J Antimicrob Chemother* 2023 doi: 10.1093/jac/dkad276 [published Online First: 20230908]

74. Boak LM, Rayner CR, Grayson ML, et al. Clinical population pharmacokinetics and toxicodynamics of linezolid. *Antimicrob Agents Chemother* 2014;58(4):2334-43. doi: 10.1128/AAC.01885-13 [published Online First: 20140210]

75. Attassi K, Hershberger E, Alam R, et al. Thrombocytopenia associated with linezolid therapy. *Clin Infect Dis* 2002;34(5):695-8. doi: 10.1086/338403 [published Online First: 20020117]

76. Gerson SL, Kaplan SL, Bruss JB, et al. Hematologic effects of linezolid: summary of clinical experience. *Antimicrob Agents Chemother* 2002;46(8):2723-6. doi: 10.1128/AAC.46.8.2723-2726.2002

77. Lin YH, Wu VC, Tsai IJ, et al. High frequency of linezolid-associated thrombocytopenia among patients with renal insufficiency. *Int J Antimicrob Agents* 2006;28(4):345-51. doi: 10.1016/j.ijantimicag.2006.04.017 [published Online First: 20060828]

78. Nukui Y, Hatakeyama S, Okamoto K, et al. High plasma linezolid concentration and impaired renal function affect development of linezolid-induced thrombocytopenia. *J Antimicrob Chemother* 2013;68(9):2128-33. doi: 10.1093/jac/dkt133 [published Online First: 20130426]

79. Shaw KJ, Barbachyn MR. The oxazolidinones: past, present, and future. *Ann N Y Acad Sci* 2011;1241:48-70. doi: 10.1111/j.1749-6632.2011.06330.x

80. Ener RA, Meglathery SB, Van Decker WA, et al. Serotonin syndrome and other serotonergic disorders. *Pain Med* 2003;4(1):63-74. doi: 10.1046/j.1526-4637.2003.03005.x

81. Gillman PK. The spectrum concept of serotonin toxicity. *Pain Med* 2004;5(2):231-2; author reply 33. doi: 10.1111/j.1526-4637.2004.04033.x

82. Butterfield JM, Lawrence KR, Reisman A, et al. Comparison of serotonin toxicity with concomitant use of either linezolid or comparators and serotonergic agents: an analysis of Phase III and IV randomized clinical trial data. *J Antimicrob Chemother* 2012;67(2):494-502. doi: 10.1093/jac/dkr467 [published Online First: 20111202]

83. Bai AD, McKenna S, Wise H, et al. Association of Linezolid With Risk of Serotonin Syndrome in Patients Receiving Antidepressants. *JAMA Netw Open* 2022;5(12):e2247426. doi: 10.1001/jamanetworkopen.2022.47426 [published Online First: 20221201]

84. Apodaca AA, Rakita RM. Linezolid-induced lactic acidosis. *N Engl J Med* 2003;348(1):86-7. doi: 10.1056/NEJM200301023480123

85. Narita M, Tsuji BT, Yu VL. Linezolid-associated peripheral and optic neuropathy, lactic acidosis, and serotonin

syndrome. *Pharmacotherapy* 2007;27(8):1189-97. doi: 10.1592/phco.27.8.1189

86. Santini A, Ronchi D, Garbellini M, et al. Linezolid-induced lactic acidosis: the thin line between bacterial and mitochondrial ribosomes. *Expert Opin Drug Saf* 2017;16(7):833-43. doi: 10.1080/14740338.2017.1335305 [published Online First: 20170601]
87. Douros A, Grabowski K, Stahlmann R. Drug-drug interactions and safety of linezolid, tedizolid, and other oxazolidinones. *Expert Opin Drug Metab Toxicol* 2015;11(12):1849-59. doi: 10.1517/17425255.2015.1098617 [published Online First: 20151012]
88. Klein EY, Jiang W, Mojica N, et al. National Costs Associated With Methicillin-Susceptible and Methicillin-Resistant Staphylococcus aureus Hospitalizations in the United States, 2010-2014. *Clin Infect Dis* 2019;68(1):22-28. doi: 10.1093/cid/ciy399
89. Nelson RE, Lautenbach E, Chang N, et al. Attributable Cost of Healthcare-Associated Methicillin-Resistant Staphylococcus aureus Infection in a Long-term Care Center. *Clin Infect Dis* 2021;72(Suppl 1):S27-S33. doi: 10.1093/cid/ciaa1582
90. Cardenas-Comfort C, Kaplan SL, Vallejo JG, et al. Follow-up blood cultures in children with Staphylococcus aureus bacteremia. *Pediatrics* 2020;146(6)
91. Iwamoto M, Mu Y, Lynfield R, et al. Trends in invasive methicillin-resistant Staphylococcus aureus infections. *Pediatrics* 2013;132(4):e817-e24.
92. Friedland IR, Du Plessis J, Cilliers A. Cardiac complications in children with Staphylococcus aureus bacteremia. *The Journal of pediatrics* 1995;127(5):746-48.
93. Duguid RC, Al Reesi M, Bartlett AW, et al. Impact of infectious diseases consultation on management and outcome of Staphylococcus aureus bacteremia in children. *Journal of the Pediatric Infectious Diseases Society* 2021;10(5):569-75.
94. Swissethics. Forschung in Notfallsituationen [Available from: https://swissethics.ch/en/templates/forschung-in-notfallsituationen accessed 24.01.2025.
95. Navarro S, Keith K, Stafylis C, et al. Safety of Linezolid During Pregnancy. *Sexually Transmitted Diseases* 2023;50(11):e37-e40. doi: 10.1097/olq.0000000000001860
96. Rowe HE, Felkins K, Cooper SD, et al. Transfer of Linezolid into Breast Milk. *Journal of Human Lactation* 2014;30(4):410-12. doi: 10.1177/0890334414546045
97. Lim FH, Lovering AM, Currie A, et al. Linezolid and lactation: measurement of drug levels in breast milk and the nursing infant. *Journal of Antimicrobial Chemotherapy* 2017;72(9):2677-78. doi: 10.1093/jac/dkx159
98. Holland TL, Chambers HF, Boucher HW, et al. Considerations for Clinical Trials of Staphylococcus aureus Bloodstream Infection in Adults. *Clin Infect Dis* 2019;68(5):865-72. doi: 10.1093/cid/ciy774
99. Ong SWX, Petersiel N, Loewenthal MR, et al. Unlocking the DOOR-how to design, apply, analyse, and interpret desirability of outcome ranking endpoints in infectious diseases clinical trials. *Clin Microbiol Infect* 2023;29(8):1024-30. doi: 10.1016/j.cmi.2023.05.003 [published Online First: 20230512]
100. Doernberg SB, Tran TTT, Tong SYC, et al. Good Studies Evaluate the Disease While Great Studies Evaluate the Patient: Development and Application of a Desirability of Outcome Ranking Endpoint for Staphylococcus aureus Bloodstream Infection. *Clin Infect Dis* 2019;68(10):1691-98. doi: 10.1093/cid/ciy766
101. Evans SR, Follmann D. Using Outcomes to Analyze Patients Rather than Patients to Analyze Outcomes: A Step toward Pragmatism in Benefit:risk Evaluation. *Stat Biopharm Res* 2016;8(4):386-93. doi: 10.1080/19466315.2016.1207561 [published Online First: 20161206]
102. Hemkens LG, Contopoulos-Ioannidis DG, Ioannidis JP. Concordance of effects of medical interventions on hospital admission and readmission rates with effects on mortality. *CMAJ* 2013;185(18):E827-37. doi: 10.1503/cmaj.130430 [published Online First: 20131021]
103. trial S-SaNAP. Core Protocol, 2021.
104. McNamara JF, Harris PNA, Chatfield MD, et al. Measuring patient-centred long-term outcome following a bloodstream infection: a pilot study. *Clin Microbiol Infect* 2020;26(2):257 e1-57 e4. doi: 10.1016/j.cmi.2019.10.011 [published Online First: 20191023]
105. Ware JE, Jr., Sherbourne CD. The MOS 36-item short-form health survey (SF-36). I. Conceptual framework and item selection. *Med Care* 1992;30(6):473-83.
106. Lins L, Carvalho FM. SF-36 total score as a single measure of health-related quality of life: Scoping review. *SAGE Open Med* 2016;4:2050312116671725. doi: 10.1177/2050312116671725 [published Online First: 20161004]
107. Chitra S, Hinahara J, Goss TF, et al. Health-Related Quality of Life as Measured by the 36-Item Short Form Survey Among Adults With Acute Bacterial Skin and Skin Structure Infections who Received Either Omadacycline or Linezolid in a Phase 3 Double-Blind, Double-Dummy Clinical Trial. *Open Forum Infect Dis* 2021;8(10):ofab459. doi: 10.1093/ofid/ofab459 [published Online First: 20210908]
108. Opota O, Croxatto A, Prod'hom G, et al. Blood culture-based diagnosis of bacteraemia: state of the art. *Clin Microbiol Infect* 2015;21(4):313-22. doi: 10.1016/j.cmi.2015.01.003 [published Online First: 20150116]

109. Rieg S, Peyerl-Hoffmann G, de With K, et al. Mortality of S. aureus bacteremia and infectious diseases specialist consultation--a study of 521 patients in Germany. *J Infect* 2009;59(4):232-9. doi: 10.1016/j.jinf.2009.07.015 [published Online First: 20090803]

110. Honda H, Krauss MJ, Jones JC, et al. The value of infectious diseases consultation in Staphylococcus aureus bacteremia. *Am J Med* 2010;123(7):631-7. doi: 10.1016/j.amjmed.2010.01.015 [published Online First: 20100520]

111. Arensman K, Dela-Pena J, Miller JL, et al. Impact of Mandatory Infectious Diseases Consultation and Real-time Antimicrobial Stewardship Pharmacist Intervention on Staphylococcus aureus Bacteremia Bundle Adherence. *Open Forum Infect Dis* 2020;7(6):ofaa184. doi: 10.1093/ofid/ofaa184 [published Online First: 20200521]

112. Goto M, Jones MP, Schweizer ML, et al. Association of Infectious Diseases Consultation With Long-term Postdischarge Outcomes Among Patients With Staphylococcus aureus Bacteremia. *JAMA Netw Open* 2020;3(2):e1921048. doi: 10.1001/jamanetworkopen.2019.21048 [published Online First: 20200205]

113. Swissethics. Forschung in Notfallsituationen [Available from: https://swissethics.ch/templates/forschung-in-notfallsituationen.

114. Doern CD, Park JY, Gallegos M, et al. Investigation of Linezolid Resistance in Staphylococci and Enterococci. *Journal of Clinical Microbiology* 2016;54(5):1289-94. doi: doi:10.1128/jcm.01929-15

115. Human UDoHa, Services NIoH, National Cancer Institute. Common Terminology Criteria for Adverse Events (CTCAE): US Department of Health and Human

Services, National Institutes of Health, National Cancer Institute., 2017.

116. U.S. DEPARTMENT OF HEALTH AND HUMAN SERVICES, National Institutes of Health, National Cancer Institute. Common Terminology Criteria for Adverse Events (CTCAE). Version 5.0 ed, 2017.

117. Pocock SJ, Ariti CA, Collier TJ, et al. The win ratio: a new approach to the analysis of composite endpoints in clinical trials based on clinical priorities. *Eur Heart J* 2012;33(2):176-82. doi: 10.1093/eurheartj/ehr352 [published Online First: 20110906]

118. Soe YM, Bedoui S, Stinear TP, et al. Intracellular and host cell death pathways. *Cellular Microbiology* 2021;23(5):e13317. doi: https://doi.org/10.1111/cmi.13317

119. Thwaites GE, Gant V. Are bloodstream leukocytes Trojan Horses for the metastasis of Staphylococcus aureus? *Nature Reviews Microbiology* 2011;9(3):215-22. doi: 10.1038/nrmicro2508

120. Jantarug K, Tripathi V, Morin B, et al. A Far-Red Fluorescent Probe to Visualize Gram-Positive Bacteria in Patient Samples. *ACS Infectious Diseases* 2024;10(5):1545-51. doi: 10.1021/acsinfecdis.4c00060

121. Scotton WJ, Hill LJ, Williams AC, et al. Serotonin Syndrome: Pathophysiology, Clinical Features, Management, and Potential Future Directions. *International Journal of Tryptophan Research* 2019;12:1178646919873925. doi: 10.1177/1178646919873925

18. APPENDICES

Documents that are periodically updated and may be updated during the course of the study, are listed here and provided separately.

18.1 Linezolid Stada® Summary of Product Characteristics

Provided separately.

18.2 The Hunter Serotonin Toxicity Criteria: for diagnosing serotonin syndrome

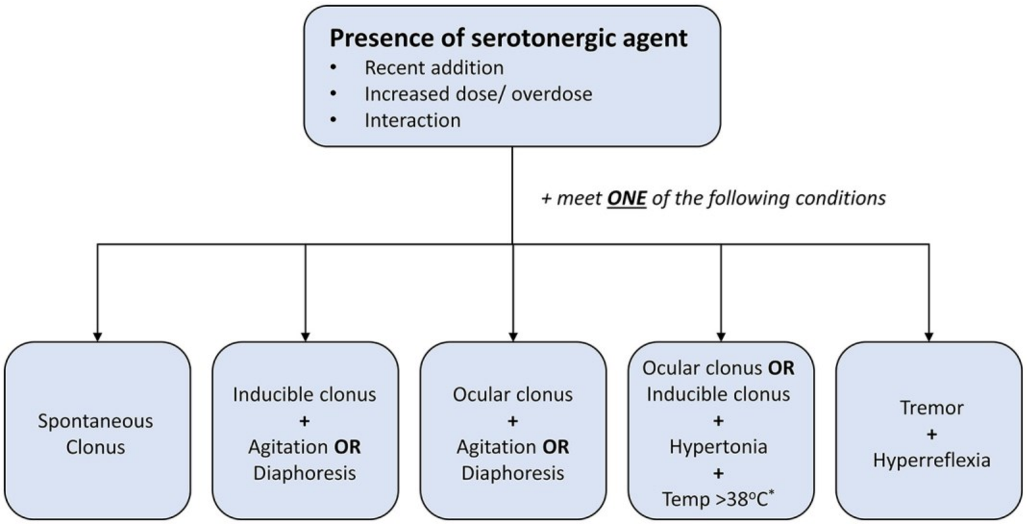

Figure 2: The Hunter Serotonin Toxicity Criteria

*Note the requirement for the presence of some form of neuromuscular excitation, the sine qua non for a diagnosis of serotonin syndrome. *The presence of temperature ⩾38.5°C and/or marked hypertonia or rigidity (especially truncal) indicates severe SS with a risk of progression with respiratory compromise.*

Taken from: Scotton, William J et al. 2019[121]
